# The Methodological Quality Scale (MQS) for intervention programs: validity evidence

**DOI:** 10.3389/fpsyg.2023.1217661

**Published:** 2023-07-06

**Authors:** Salvador Chacón-Moscoso, Susana Sanduvete-Chaves, José Antonio Lozano-Lozano, Francisco Pablo Holgado-Tello

**Affiliations:** ^1^Departamento de Psicología Experimental, Facultad de Psicología, Universidad de Sevilla, Sevilla, Spain; ^2^Departamento de Psicología, Universidad Autónoma de Chile, Santiago, Chile; ^3^Instituto de Ciencias Biomédicas, Universidad Autónoma de Chile, Santiago, Chile; ^4^Departamento de Metodología de las Ciencias del Comportamiento, Facultad de Psicología, Universidad Nacional de Educación a Distancia, Madrid, Spain

**Keywords:** methodological quality, scale, meta-analysis, program evaluation, reliability, validity

## Abstract

**Introduction:**

A wide variety of instruments are used when assessing the methodological quality (MQ) of intervention programs. Nevertheless, studies on their metric quality are often not available. In order to address this shortcoming, the methodological quality scale (MQS) is presented as a simple and useful tool with adequate reliability, validity evidence, and metric properties.

**Methods:**

Two coders independently applied the MQS to a set of primary studies. The number of MQ facets was determined in parallel analyses before performing factor analyses. For each facet of validity obtained, mean and standard deviation are presented jointly with reliability and average discrimination. Additionally, the validity facet scores are interpreted based on Shadish, Cook, and Campbell’s validity model.

**Results and discussion:**

An empirical validation of the three facets of the MQ (external, internal, and construct validity) and the interpretation of the scores were obtained based on a theoretical framework. Unlike other existing scales, MQS is easy to apply and presents adequate metric properties. In addition, MQ profiles can be obtained in different areas of intervention using different methodologies and proves useful for both researchers doing meta-analysis and for evaluators and professionals designing a new intervention.

## 1. Introduction

The concept methodological quality (MQ) can be defined as the degree to which a study can avoid systematic errors (bias), and the degree to which we are sure that such study can be believed (Reitsma et al., 2009). Measuring MQ is important to foster accumulative knowledge given the relationship between MQ and effect size, where effect size is higher when MQ is low; i.e., low MQ studies tend to overestimate the effectiveness of interventions (Hempel et al., 2011). Thus, when multiple interventions lack MQ, it becomes difficult to reach trustworthy conclusions (Chacón-Moscoso et al., 2014).

In meta-analytical research, the results of different primary studies on a specific issue or research question are quantitatively integrated (Cooper et al., 2009). Generally, the MQ of primary studies is measured with the intent of evaluating the credibility of the results obtained in the meta-analysis (Luhnen et al., 2019). On some occasions, low MQ is an exclusion criterion.

Measuring MQ is not just useful for integrating finished interventions in meta-analysis. In the context of program evaluation, it is also fundamental to increase the MQ of the design, the implementation, and the evaluation of ongoing and future intervention programs. Finally, when several different interventions are feasible, it allows the most adequate to be chosen based on the target population, the aims, and the context (Cano-García et al., 2017).

Thus, a wide variety of professionals need to measure MQ. In most cases, these professionals are not experts in methodological issues, and when they seek out an instrument to gauge MQ, they encounter a wide variety of them (Higgins et al., 2013). There are two reasons why experts in methodology are unable to offer a simple way to assess quality.

First, a plethora of strategies for assessing the MQ of primary sources can be found in the literature (see, for example, www.equator-network.org). At present, we can affirm that around 100 quality scales have been identified (Conn and Rantz, 2003) as well as more than 550 different strategies to measure MQ (Chacón-Moscoso et al., 2016).

In some settings, such as medicine, a certain consensus has been reached on the use of individual quality components or items not combined into scales (Herbison et al., 2006), and researchers in the social sciences also appear to be increasingly forgoing scales (Littell et al., 2008). However, no empirical tests have been conducted in other areas such as psychology, which is problematic given how this decision affects the replicability of study results (Anvari and Lakens, 2018).

Second, discrepant results were found when applying several measurement strategies in the same sample of primary studies (Losilla et al., 2018). Depending on the instrument chosen, the assessment of MQ may vary. As a result, the choice of scale can lead us to treat a study differently, rely on its results to varying degrees, or even include/exclude it from a meta-analysis.

The discrepancies between the quality scales may be attributed to different causes: the scales measure different aspects of quality, have been constructed from different research contexts (Albanese et al., 2020), or present metric deficiencies (Sterne et al., 2016). This is because the makers of these scales did not follow the standards for developing measuring instruments, and their metric properties are generally unexplored.

This paper considers MQ based on its existing use and applications in the literature. Our approach, which draws on consequential validity (Brussow, 2018) and is centered on the descriptive theory of valuation, aims to describe values without evaluating whether any one is better than others (Shadish et al., 1991). For this purpose, we developed the 12-item MQ Scale (MQS) (Chacón-Moscoso et al., 2016). We did an exhaustive review of the literature (Nunnally and Bernstein, 1994) and compiled 550 different strategies to assess MQ (the list of bibliographic references is available in Supplementary material S1). Subsequently, we selected the most frequent indicators of MQ, obtaining 23 items. A content validity study was then carried out. Thirty experts in meta-analysis and/or methodology participated voluntarily. All were methods group members of the Campbell Collaboration and/or the European Association of Methodology. Participants (12 women and 18 men, 20 from Europe and 10 from the United States) were contacted by e-mail or face-to-face in the biannual congresses of the associations. Their mean age was 42, with an average of 14 years of experience on these issues. Participants evaluated the representativeness, utility, and feasibility of each item with respect to a hypothetical global construct of MQ. Finally, the 12 items that passed the cutoff point were selected and refined after an intercoder reliability study (the final version of the instrument, used as a coding manual for this work, is available in Supplementary material S2).

An advantage to this approach is that we specify the origin and reasons for the selection of these final items and, additionally, these were not limited to any particular intervention, methodology, or context. Furthermore, to bypass the common handicap of presenting a proposal without a thorough study of its metric properties, the aim of this paper was to analyze the metric properties of the scores obtained with the MQS in terms of reliability and validity evidence, as well as its dimensional structure.

As the concept of MQ is multidimensional, we hypothesized that we were not going to find a general factor that explained the set of 12 items. Instead, we approached the empirical study of the possible different facets (profiles) of the validity evidence of these 12 items based on a conceptual validity framework (Shadish et al., 2002), and the structural dimensions that form the acronym UTOSTi; Units, Treatment, Output, Setting and Time (Chacón-Moscoso et al., 2014, 2021). Finally, we present an application in organizational training programs based on the interpretation of the scores obtained in such validity facets.

## 2. Methods

### 2.1. Participants

Studies on training programs for workers in organizations were selected as a topic that has attracted substantial research interest (Sanduvete-Chaves et al., 2009). A total of 299 full texts were selected. References from these studies are available in Supplementary material S3. Each study had to meet the following inclusion criteria: their research topic was training programs for workers at organizations; non-duplicated; written in English or Spanish; full text available; primary study; empirical study in which a training program was applied; and the training program was the aim of the study.

### 2.2. Instruments

MQS, available in Supplementary material S2, was applied. This scale presented 12 items, each with three alternatives (0, 0.5, and 1) representing, respectively, the null, medium, or total level of achievement of the criterion presented in the item.

### 2.3. Procedure

The search cut-off date for the primary studies was June 2020. The following databases were included because of the relevant issues they cover: Web of Science, SCOPUS, Springer, EBSCO Online, Medline, CINAHL, Econlit, MathSci Net, Current Contents, ERIC, and PsycINFO. The combined keywords were “evaluation” AND “work” AND “training programs,” with a search in title, abstract, keywords, and complete article. Additionally, authors who published most frequently on training programs for workers were contacted by e-mail to ask if they could share any other work, published or unpublished, on this topic.

During the initial screening, the inclusion criteria were applied to title, keywords, and abstract. The included studies were evaluated at a second stage, applying the inclusion criteria to the full texts. Two coders (SSC and FPHT) applied the criteria independently. In case of disagreements, a third coder (SCM) mediated to reach a consensus.

For the data extraction, the same two coders participated in 2 months of training sessions until the appropriate inter-coder reliability was met, with *κ* agreement greater than 0.7 in a pilot study. Subsequently, they coded the entire sample of selected studies. Finally, the discrepancies between coders were resolved by involving a third researcher (SCM) to reach a consensus.

### 2.4. Data analyses

Using SPSS v.26, we conducted an intercoder reliability analysis after the selection phase and data extraction phase. Kappa (*κ*) coefficient, a statistic specifically created to value inter-rater agreement which corrects the probability of concordance due to hazard (McHugh, 2012), was computed for each item and 95% confidence intervals. *κ* between 0.61 and 0.80 was considered substantial; and above 0.80, very good (Landis and Koch, 1977). We then performed descriptive analyses of item scores, calculating the mean, the standard deviation, skewness, and kurtosis coefficients.

To obtain the validity facets that were implicit in the tool, the FACTOR software (Ferrando and Lorenzo-Seva, 2017) was used. First, a parallel analysis was done using optimal implementation to determine the number of dimensions (Timmerman and Lorenzo-Seva, 2011; Yang and Xia, 2015); second, Exploratory Factor Analyses (EFA) were performed to extract the main dimensions (Ferrando and Lorenzo-Seva, 2018). The polychoric correlation matrix (Holgado-Tello et al., 2010) was used because of the ordinal metric of the variables and the non-normal data distribution. Unweighted least squares were applied as the estimation method and varimax rotation (Sanduvete-Chaves et al., 2013, 2018).

Using the JASP version 0.16 software (JASP Team, 2021), the reliability of the test scores was examined for each dimension obtained by calculating the McDonald’s omega (*ω*) coefficient. For item discrimination, we computed corrected item-total correlation coefficients.

In addition, the theoretical interpretation of each extracted dimension was analyzed according to its items. Thus, the correlation between items, the factor solution, the metric features of the dimensions, and the theoretical congruence were considered to obtain the different dimensions.

Once the validity facets were obtained, we presented their descriptive statistics (mean, standard deviation, reliability, and average discrimination). Finally, a theoretical interpretation was performed for the primary studies analyzed.

## 3. Results

### 3.1. Selection of the studies

**Figure 1:**
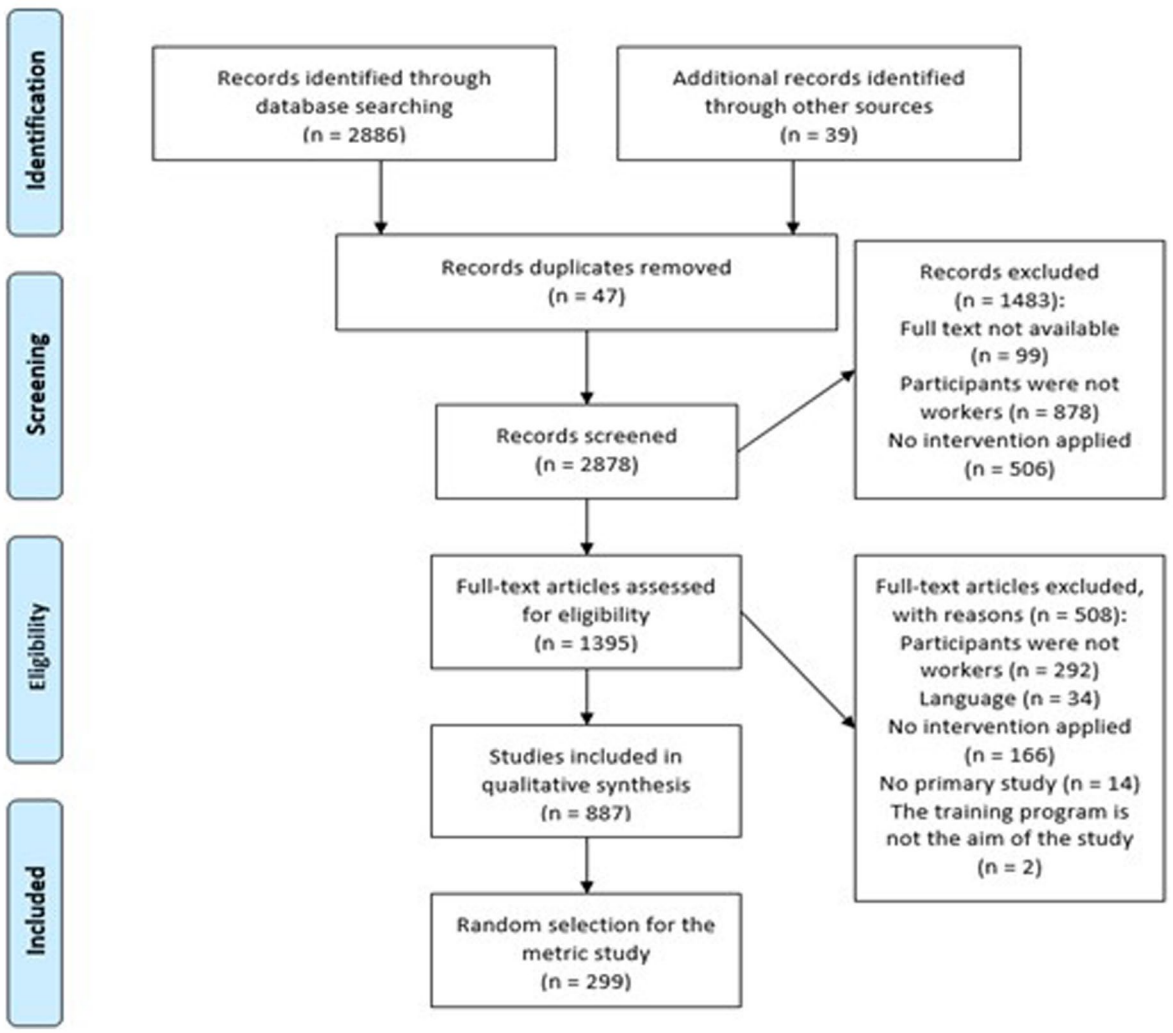
PRISMA Flow chart of the study selection process (Page et al., 2021).

Figure 1 summarizes the selection process. A total of 2,886 studies were found in database searches and 39 were sent by the authors contacted by e-mail. Of the 2,878 nonduplicated papers found, 887 met the inclusion criteria, 299 of which were selected at random.

### 3.2. Intercoder reliability

In the study search, intercoder reliability was *κ* = 0.705. *p* < 0.001, 95% CI [0.674, 0.736]. Table 1 presents *κ* values with their significance and confidence intervals that refer to the information extraction phase. *κ* in items varied between 0.651 and 0.949, with an average of *κ* = 0.910. *p* < 0.001, 95% CI (0.898, 0.922). All items obtained adequate results.

**Table 1:** Intercoder reliability and descriptive statistics of the items.

	Intercoder reliability			Descriptive statistics					
Item	Kappa	*LL*	*UL*	*M*	*Mdn*	*SD*	S	K	SW
1	0.651	0.543	0.759	0.89	1	0.21	−1.53	0.77	0.51
2	0.912	0.869	0.955	0.27	0	0.35	0.90	−0.45	0.72
3	0.783	0.679	0.887	0.89	1	0.30	−2.46	4.32	0.4
4	0.784	0.633	0.935	0.78	1	0.41	−1.35	−0.08	0.55
5	0.798	0.412	1.184	0.99	1	0.04	−12.14	145.97	0.05
6	0.938	0.899	0.977	0.24	0	0.38	1.26	−0.13	0.63
7	0.949	0.918	0.980	0.52	0.5	0.39	−0.05	−1.32	0.81
8	0.86	0.768	0.952	0.91	1	0.22	−2.309	4.78	0.46
9	0.86	0.811	0.909	0.51	0.5	0.44	−0.04	−1.73	0.75
10	0.775	0.673	0.877	0.57	0.5	0.18	1.85	2.24	0.44
11	0.809	0.735	0.883	0.87	1	0.23	−1.52	1.25	0.55
12	0.884	0.829	0.939	0.36	0	0.48	0.60	−1.65	0.61

S, skewness; K, kurtosis; SW, shapiro-wilk normality test. All Kappa and SW obtained *p* < 0.001.

### 3.3. Descriptive analysis

The database used in this article is available in Supplementary material S4. Table 1 presents descriptive statistics for the 12 items. The distributions obtained for each of the items highlighted their skewness. The median was 1 for most items. The means ranged between 0.24 and 0.99, the standard deviations were between 0.04 and 0.48, and there was no normal distribution of the items.

Items 5 and 8 presented means over 0.9, which implies that they lack the capacity to discriminate. Items 1, 5, 8, 10, and 11 obtained low variability, with SD below 0.25 (for example, in item 5, 99% of the studies fell into the category 1). Skewness was negative and less than −2.3 for items 3, 5 and 8. Finally, kurtosis exceeded 4.3 for items 3, 5, and 8.

To analyze the relationship between items, Table 2 presents the bivariate polychoric correlation matrix. Based on the associations between items, the highest positive bivariate correlations were between items 6 and 7 (*r* = 0.77) and between items 9 and 11 (*r* = 0.73). Additionally, items 5 and 8 were related (*r* = 0.47) and behaved differently than the remaining items, since their correlations with the others were negative and/or low. This may be related to the small discrimination capacity and variability that items 5 and 8 presented in Table 1.

**Table 2:** Polychoric correlation matrix.

Item	1	2	3	4	5	6	7	8	9	10	11
1	1										
2	0.30	1									
3	0.41	0.32	1								
4	−0.20	−0.73	0.03	1							
5	−0.07	−0.33	−0.95	0.38	1						
6	0.01	0.58	0.22	−0.14	−0.07	1					
7	0.08	0.64	0.33	−0.29	−0.62	0.77	1				
8	−0.04	−0.33	−0.11	0.09	0.47	−0.35	−0.67	1			
9	0.29	0.42	0.25	−0.47	−0.32	0.21	0.42	−0.21	1		
10	0.48	0.41	0.14	−0.29	0.07	0.09	0.14	−0.01	0.21	1	
11	0.33	0.51	0.29	−0.66	−0.55	0.23	0.36	−0.07	0.73	0.21	1
12	0.35	−0.07	0.52	0.08	−0.22	−0.16	−0.15	0.19	0.04	0.06	0.19

### 3.4. Study of dimensionality

A parallel analysis was conducted to obtain empirical evidence about the number of factors that the scale presented (see Table 3). The results suggested no unidimensionality.

**Table 3:** Parallel analysis.

Variable	% of *S*^2^ in real data	Mean random % of *S*^2^	95 *P* random % of *S*^2^
1	19.91*	17.66	20.38
2	15.98*	15.35	17.31
3	13.54*	13.48	14.85
4	12.94*	11.74	12.82
5	10.25*	10.18	11.37
6	7.69	8.72	9.82
7	6.55	7.34	8.39
8	5.45	6.03	7.29
9	4.16	4.62	5.76
10	2.27	3.15	4.45

*P*, percentile.

Next, we set out to identify the relevant factors from the twelve items, based on the chosen validity framework. Based on the previous parallel analysis, the first EFA conducted to extract dimensions was set to five factors. The rotated loadings (see Table 4), interpreted from a theoretical point of view, led us to form a dimension composed of items 1 (inclusion and exclusion criteria for the units), 3 (attrition), 4 (attrition between groups), and 12 (statistical methods for imputing missing data). This dimension (factor 1 -F1-) could be interpreted as a measure of external validity, as items are focused on the representativeness of participants from a delimited population, selection criteria, the possible problem of a loss of participants during the study, and the method used to compute any missing data.

**Table 4:** Rotated matrix (exploratory factor analysis) set to five factors.

Item	Factor 1	Factor 2	Factor 3	Factor 4	Factor 5
1	0.48		−0.44		0.34
2		0.22			0.57
3	0.58	0.31	−0.21		−0.24
4	0.98				
5			0.97		
6		0.53			
7		0.97			
8			0.33		
9		0.31	0.23	0.45	
10					0.99
11				0.97	
12	0.63				0.63

Loadings lower than absolute 0.20 were omitted.

To guarantee that the four items mentioned (items 1, 3, 4, and 12) could be interpreted as a single dimension, a parallel analysis was conducted, introducing these four items exclusively. According to the results, a single dimension was recommended. The reliability of this dimension was *ω* = 0.60, and the discrimination of the items was 0.21 for item 1, 0.45 for items 3 and 4, and 0.25 for item 12.

Once F1 was defined, the next step was to obtain empirical evidence that could statistically support other possible factors. Thus, a new EFA was conducted after omitting the items that formed F1 (items 1, 3, 4, and 12). The aim was to extract the next most relevant factor, avoiding redundant variability that could hamper its interpretation. Following this procedure, and after interpreting the results shown on Table 4, a second factor (F2) that could be interpreted as internal validity was obtained (Shadish et al., 2002). This second factor was formed by items 2 (methodology or design), 6 (follow-up period), 7 (measurement occasions for each dependent variable), and 10 (control techniques). These items focus on the level of manipulation, the number of groups, the measurements of relevant dependent variables to be measured, and the techniques applied to control for potential sources of error. As shown on Table 2, the bivariate correlations between items 2, 6 and 7 are high, between 0.58 and 0.77.

A parallel analysis conducted only with these items (items 2, 6, 7, and 10) yielded a single dimension. The reliability coefficient of F2 was *ω* = 0.70. The inclusion of item 10 negatively affected the reliability of F2 (*ω* = 0.77 without item 10); however, from a content validity perspective, it was considered that the information contained in item 10 was relevant to define this dimension, as it referred to control techniques directly related to internal validity. The discriminations of the items were 0.56 (item 2), 0.55 (item 6), 0.65 (item 7), and 0.17 (item 10).

Once F1 and F2 were defined, a third EFA was performed with the remaining items 5, 8, 9, and 11. The results, strongly supported by the theory and according to results obtained in Table 4, showed a dimension defined by items 9 and 11. Additionally, they presented a high correlation in Table 2 (*r* = 0.73). These items measured the standardization of the dependent variables (item 9), and the construct definition (item 11). Therefore, this dimension (factor 3 -F3-) was interpreted as construct validity (Shadish et al., 2002), because it is focused on explaining the concept, model, or schematic idea measured as a dependent variable, the way the theoretical dimensions are empirically defined, and the standardization of the tool used to measure the dependent variable.

Based on the parallel analysis conducted after including items 9 and 11, a single dimension was recommended. The reliability of F3 was *ω* = 0.65 and the discrimination of the two items was 0.48.

Based on the results obtained in Table 4, items 5 (exclusions after assignment) and 8 (measures in pretest appear in post-test) were difficult to integrate, though they appeared to be linked. If we hypothesize that, together, these items form a dimension, its metric indices would be very low, with a reliability coefficient of *ω* = 0.13 and discrimination indexes of 0.07. These items were predicted to present problems, since they had no variability, did not discriminate between studies, and presented excessive skewness, as shown on Table 1. We decided to exclude these two items from the defined F3 because they did not fit items 9 and 11, and presented low theoretical congruence in this dimension.

### 3.5. Interpretation of the study scores in each validity facet and acquisition of possible profiles

Table 5 shows the descriptive statistics for each of the theoretical validity facets obtained. To interpret the items in the study, each received a score of 0 (low), 0.5 (medium) or 1 (high).

**Table 5:** Descriptive statistics for each facet.

	F1	F2	F3
Possible range	0–4	0–4	0–2
Mean	3.98	1.59	1.38
Standard deviation	0.89	0.96	0.59
McDonald’s ω	0.60	0.70	0.65
Discrimination	0.34	0.48	0.48

F1, external validity facet; F2, internal validity facet; F3, construct validity facet.

Formed by items 1 (inclusion and exclusion criteria for units), 3 (attrition), 4 (attrition between groups), and 12 (statistical methods for imputing missing data), F1 assesses external validity. F1 answers the question: how accurately are the population and the selection criteria for units defined? Studies with high scores in F1 should be characterized by a well-defined reference population, explicit selection criteria for the units that form the sample, and the monitoring of possible unit losses over the course of the study that could compromise the representativeness of the results.

Formed by items 2 (methodology or design), 6 (follow-up period), 7 (measurement occasions for each dependent variable), and 10 (control techniques), F2 assesses internal validity. It answers the questions: What are the relevant variables of the study? How and when are they manipulated and measured? What is done to control for possible sources of error? Studies with a great capacity to manipulate variables and control for threats to validity (Holgado-Tello et al., 2016) would receive high scores in F2. Other factors that affect scores in F2 include clearly established criteria for assigning the units to study conditions and the quantity of measures before, during, and after the interventions.

Finally, F3 assesses construct validity. Formed by items 9 (standardization of the dependent variables) and 11 (construct definition of outcomes), it answers the question: How are dimensions empirically operationalized from their conceptual referents? Studies with high scores in F3 clearly present the referent or conceptual model, an empirical operationalization of its components, and standardized measurements.

Table 6 provides an example of how scores can be interpreted in each study based on the scores for each item, as well as an overall assessment of the study based on its mean per facet. The scores of the facets were obtained calculating the average of the items that comprised them. This average ranged from 0 to 1 (low if <0.5; medium if ranging from 0.5–75, both values included; and high for values >0.75). For example, study 3 had a score of 2 in F1 (average = 0.5; medium quality); in F2, 1.5 (average = 0.37; low quality); and in F3, 1.5 (average = 0.75, medium quality).

**Table 6:** Scores of four studies in each item, and average values in each facet.

	Validity facets										Global		
Studies	F1				F2				F3		Facets		
	I_1_	I _3_	I _4_	I _12_	I _2_	I _6_	I _7_	I _10_	I _9_	I _11_	F1	F2	F3
3	1	1	0	0	0.5	0	0.5	0.5	0.5	1	0.5	0.37	0.75
6	1	1	1	0	1	1	1	0.5	1	1	0.75	0.88	1
138	0.5	0.5	–	0	0	0	0	0.5	0	0.5	0.33	0.12	0.25
299	1	1	1	1	1	0	1	1	1	1	1	0.75	1

F1, external validity facet; F2, internal validity facet; F3, construct validity facet; I, item. Red, low level; yellow, medium level; green, high level of quality.

**Figure 2:**
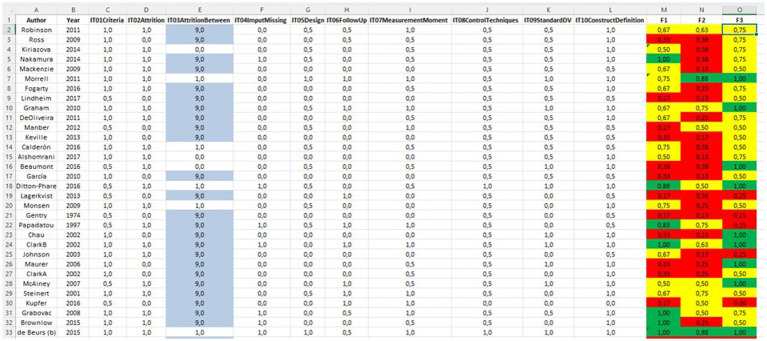
Example of primary study coding. 9, not applicable. IT, item; F1, external validity facet; F2, internal validity facet; F3, construct validity facet. Red, low level; yellow, medium level; green, high level of quality. The complete items are available in Table 8.

The evaluations of the 299 coded studies, by items and facets, are available in Supplementary material S4. Figure 2 represents part of this database, showing one line for each study, scores (0, 0.5 or 1) for each item, and the average score in each facet.

Table 7 presents the frequencies and percentages of studies of the sample that had a low, medium, and high level of quality in each facet. Based on the results obtained, most of the studies that comprised the sample presented medium levels of quality in external validity, low levels in internal validity, and high levels in construct validity.

**Table 7:** Distribution of studies by quality level in each facet (frequencies and %).

Level of quality	F1 External v.	F2 Internal v.	F3 Construct v.
Low	35 (11.7)	185 (61.9)	56 (18.7)
Medium	127 (42.5)	63 (21.1)	71 (23.8)
High	137 (45.8)	51 (17.0)	172 (57.5)
*Total*	*299 (100)*	*299 (100)*	*299 (100)*

F, facet; v., validity. Percentages are presented in brackets. For each facet (F1, F2, and F3), the most frequent level (low, medium, or high) is marked.

Table 8 presents the resulting MQS, ready to be used to measure the MQ in primary studies (MQS is also available in a printable version in Supplementary material S5).

**Table 8:** Methodological quality scale (final version).

**Facet 1. External validity**		
Item 1	**Inclusion and exclusion criteria for the units provided**: explicit reasons provided as to why certain units (usually people) were able to participate in the study and others were not: **0. No:** no explicit selection criteria for units AND with exceptions in their application; information unavailable. **0.5. Intermediate:** explicit selection criteria for units OR applied to all potential participants. **1. Yes (replicable):** explicit selection criteria for units AND applied to all potential participants.	
Item 2	**Attrition:** loss of units. In randomized experiments, this refers to loss that occurred after the random assignment, i.e., the number of participants from the initial sample that did not conclude the study (e.g., N pre minus N post). **0. Unspecified:** information is not available and cannot be calculated AND reasons for loss of units are not specified. **0.5. Intermediate:** number of units lost is specified or can be calculated OR reasons for loss of units are specified. **1. Specified:** no units are lost, or number of units lost is specified or can be calculated AND reasons for loss of units are specified.	
Item 3	**Attrition between groups:** this item evaluated the differences in attrition between two groups. **0. Unspecified:** information is not available and cannot be calculated AND reasons for attrition between groups are not specified. **0.5. Intermediate:** number of lost units is specified or can be calculated OR reasons for attrition between groups are specified. **1. Specified:** no units were lost, or number of lost units is specified or can be calculated AND reason/s for the attrition between groups is/are specified. **9. Not applicable:** no cross-group comparison.	
Item 4	**Statistical methods for imputing missing data**: to estimate what the study would have yielded had there been no attrition: **0. High risk:** it is not clear if there was attrition, or there was attrition and calculations to estimate effects were carried out without imputing missing data. **0.5. Medium risk:** values for the missing data points were imputed so they could be included in the analyses. The method used was specified, i.e., sample mean substitution, last value forward method for longitudinal data sets, hot deck imputation, single imputation (e.g., imputation, regression imputation), or multiple imputation (e.g., likelihood ratio test after multiple imputation). The reasons for choosing the specific method were not specified. **1. Low risk:** there was no attrition or values for the missing data points were imputed so they could be included in the analyses; and the specific method used AND the reasons for choosing the specific method were specified.	
Total facet 1	**External validity score:** Add the scores obtained in items 1–4 and divide by the number of items. If item 3 is not applicable, do not add a score for that item and divide the sum of items 1, 2 and 4 by 3.	
**Facet 2. Internal validity**		
Item 5	**Methodology or design:** something an experimenter could manipulate or control in an experiment to help address a threat to validity: **0. Pre-experimental/others** (questionnaires/observational/naturalistic): a study with only one group and a maximum of two measurement occasions for the same dependent variable (e.g., pre-post design); or when there are two groups and only one measure (e.g., control-experimental design). **0.5. Quasi-experimental** (two groups without randomized assignment) non-equivalent control groups with pre-test and post-test; or one group with three or more measures of the same dependent variable (even without pretest): an experiment (exploration of the effects of manipulating a variable) in which units are not randomly assigned to conditions. **1. Experimental; randomized:** an experiment (exploration of the effects of manipulating a variable) in which units are randomly assigned to conditions.	
Item 6	**Follow-up period**: the amount of time between the first post-intervention measurements and any additional measurements. When the study presented more than one follow-up period, the longest was considered. **0.** No follow-up or less than 2 months. **0.5.** Between two and 6 months (both included). **1.** More than 6 months.	
Item 7	**Measurement occasions for each dependent variable**: this item specified when the measurements were taken. **0. Post-intervention only:** all measurements were taken after the intervention. **0.5. Pre- and post-intervention:** some measurements were taken before and immediately after the intervention. **1. Pre-, post-intervention and follow-up period:** some measurements were taken before, immediately after the intervention, and again at a later date.	
Item 8	**Control techniques**: **0. None:** no control technique is specified or described. **0.5 Masking OR other/s:** masking, also known as double-blinding, refers to a procedure that prevented participants and/or experimenters from knowing the hypotheses; OR any other control technique was used (e.g., matching, stratifying, counterbalancing, constant, participant as own experimental control -longitudinal-). **1. Masking AND other:** masking AND at least one other control technique.	
Total facet 2	**Internal validity score:** Add the scores obtained in items 5–8 and divide by the number of items (4).	
**Facet 3. Construct validity**		
Item 9	**Standardization of the dependent variables:** level of normalization of the tool to measure the variable that varied in response to the independent variable (also called effect or outcome). **0. Low standardization (self-reports and *post hoc* records)**: all measurements were taken using *ad hoc* tools, developed in a specific situation, and without any study of their psychometric properties. **0.5. Medium standardization**: at least one measurement was taken using structured tools with ONE study of their psychometric properties (reliability or one form of validity evidence). **1. High standardization**: at least one measurement was taken using structured tools. At least TWO studies of their psychometric properties (reliability, validity, construction of scaling) were carried out.	
Item 10	**Construct definition of outcome**: explanation of the concept, model, or schematic idea measured as a dependent variable: **0. No definition:** no concept treated as a dependent variable was measured in a conceptual or empirical way. **0.5. Vague definition:** at least one concept treated as a dependent variable was defined in a conceptual and/or empirical way. **1. Replicable by reader in own setting:** all concepts treated as dependent variables were defined in a conceptual and empirical way.	
Total facet 3	**Construct validity score:** Add the scores obtained in items 9 and 10 and divide by the number of items (2).	
**INTERPRETATION for each type of validity (facet):**		
<0.5 Low	[0.5–0.75] Medium	>0.75 High

## 4. Discussion

This work offers a practical approach to solve an existing problem, i.e., how to measure the varying quality levels of primary studies. It does so by analyzing the metric properties of a scale based on the standards for the constructions of measuring instruments that guarantee validity and reliability; not only analyzing content validity and intercoder reliability, but also including validity evidence based on the internal structure of the scale and metric properties of the tool (reliability based on internal coherence and discrimination). The proposed tool is available for researchers who are planning to carry out a meta-analysis. Additionally, it presents the basic elements to assess MQ of intervention programs, so professionals who are not experts in methodology can use the tool to design a new intervention or to evaluate an ongoing or completed intervention. Thus, this tool represents a first step toward guaranteeing that meta-analyses and interventions respond to replicability criteria.

In terms of other advantages, it is important to highlight that the inclusion criteria for the initial items of the MQS were specified. It not only considers the risk of bias associated with internal validity, but more broadly, that associated with external and construct validity. This yielded profiles with three facets, thus facilitating interpretation. It is a tool that can be applied to any type of intervention study (i.e., not only experimental methodology or randomized control trials). It is applicable in different areas of interest (not only in a specific setting). Moreover, it is easy to apply, as it is formed by ten items with three-point Likert scales.

A potential limitation of this study is that the MQS has only been applied to a set of studies in a specific field of intervention. However, regardless on the field of intervention, MQ varies between studies. For example, randomized studies with a high manipulation of variables can be found in in the field of health and in the social sciences too. MQ in itself is not related to the field of intervention. For this reason, we consider that MQ indicators can be studied in any context.

Additionally, the fact that the construct validity is comprised of only two items could be considered another limitation. However, one of the main ideas was to reduce the number of items in the scale as much as possible without lower its metric properties; this facet presents adequate validity and reliability indexes. Additionally, other tools (e.g., Valentine and Cooper, 2008), contain only one item on construct validity (i.e., face validity).

In relation to items 5 and 8, it was not possible to justify a single factor with adequate metric indexes, such as statistical conclusion validity, based on the results obtained. Nonetheless, the content of item 5 (exclusions after assignment) can be considered that is included in items referred to attrition (items 3 and 4 -external validity-). Moreover, the content of item 8 (measures in pretest appear in posttest) can be considered that is included in items referred to methodology and follow-up (items 2 and 6, respectively -internal validity-).

For further research, the same sample of primary studies will be coded using other tools available in the literature to compare the results. The Risk of Bias version 2 (RoB 2) (Sterne et al., 2019) will be applied for experimental designs; and for quasi-experimental designs, the Risk Of Bias In Non-randomised Studies (ROBINS-I) (Sterne et al., 2016). Additionally, a cross-disciplinary guide will be drafted to inform practitioners of the design, implementation, and evaluation of intervention programs.

## Data availability statement

The datasets presented in this study can be found in online repositories. The names of the repository/repositories and accession number(s) can be found in the article/Supplementary material.

## Author contributions

SC**-**M conceived of and designed the study, analyzed and interpreted the data, and wrote the first draft and revised it. SS**-**C collaborated in the acquisition of data and critically reviewed the drafting for important intellectual content. JL**-**L contributed to the data acquisition, analysis, and interpretation and reviewed the paper. FH**-**T analyzed and interpreted the data and collaborated on the writing of the article. All authors contributed to the article and approved the submitted version.

## Funding

This work was supported by the Chilean national projects FONDECYT Regular 2019, Agencia Nacional de Investigación y Desarrollo (ANID)–, Government of Chile (1190945); the grant PID2020-115486GB-I00 funded by the Ministerio de Ciencia e Innovación, MCIN/AEI/10.13039/501100011033, Government of Spain; and the grant PID2020-114538RB-I00, funded by the Ministerio de Ciencia e Innovación, Government of Spain.

## Conflict of interest

The authors declare that the research was conducted in the absence of any commercial or financial relationships that could be construed as a potential conflict of interest.

## Publisher’s note

All claims expressed in this article are solely those of the authors and do not necessarily represent those of their affiliated organizations, or those of the publisher, the editors and the reviewers. Any product that may be evaluated in this article, or claim that may be made by its manufacturer, is not guaranteed or endorsed by the publisher.
