# Supplementary material for: The Methodological Quality Scale (MQS) for intervention programs: validity evidence
Source: Front Psychol. 2023 Jul 6;14:1217661. doi: 10.3389/fpsyg.2023.1217661 (PMC10358327; doi:10.3389/fpsyg.2023.1217661)
Supplement: Supplementary file 1 [file Data_Sheet_1.DOCX]

Supplementary Material

The Methodological Quality Scale (MQS) for Intervention Programs: Validity Evidence

**Salvador Chacón-Moscoso*, Susana Sanduvete-Chaves, José Antonio Lozano-Lozano, Francisco Pablo Holgado-Tello**

*** Correspondence:** Salvador Chacón Moscoso: schacon@us.es

# Supplementary Data S1

This content is also available at https://www.frontiersin.org/articles/10.3389/fpsyg.2016.01811/full#supplementary-material as Supplementary Data Sheet 1

Papers that contain measurement of quality in primary studies:

Advance Bladder Cancer (ABC) Meta-analysis Collaboration. (2005). Adjuvant chemotherapy in invasive bladder cancer: A systematic review and meta-analysis of individual patient data advanced bladder cancer (ABC) meta-analysis collaboration. *European Urology, 48*(2), 189-201.

Albrecht, L., Archibald, M., Arseneau, D., & Scott, S. D. (2013). Development of a checklist to assess the quality of reporting of knowledge translation interventions using the Workgroup for Intervention Development and Evaluation Research (WIDER) recommendations. *Implementation Science, 8*. Retrieved from <http://www.implementationscience.com/content/8/1/52>

Aletaha, D., Landewe, R., Karonitsch, T., Bathon, J., Boers, M., Bombardier, C., . . . Felson, D. (2008). Reporting disease activity in clinical trials of patients with rheumatoid arthritis: EULAR/ACR collaborative recommendations. *Annals of the Rheumatic Diseases, 67*(10), 1360-1364.

Alexander, D. D., & Cabana, M. D. (2010). Partially Hydrolyzed 100% Whey Protein Infant Formula and Reduced Risk of Atopic Dermatitis: A Meta-analysis. *Hepatology and Nutrition, 50*, 422–430.

Alexander, D. D., Mink, P. J., Cushing, C. A., & Sceurman, B. (2010). A review and meta-analysis of prospective studies of red and processed meat intake and prostate cancer. *Nutrition Journal, 2*, 9. Retrieved from <http://www.nutritionj.com/content/9/1/50>

Altman, D. G., Schulz, K. F., Moher, D., Egger, M., Davidoff, F., Elbourne, D., . . . Lang, T. (2001). The revised CONSORT statement for reporting randomized trials: explanation and elaboration. *Annals of Internal Medicine, 134*(8), 663-694.

American Educational Research Association (2006). Standards for reporting on empirical social science research in AERA publications. *Educational Researcher, 35*(6), 33–40.

American Psychological Association (2010). *Publication manual of the American Psychological Association* (6th ed.). Washington, DC: Author.

# American Psychological Association Publications and Communications Board Working Group on Journal Article Reporting Standards (2008). Reporting standards for research in psychology. Why do we need them? What might they be? *American Psychologist, 63*(9), 839-851.

Angelillo, I. F., & Villari, P. (1999). Residential exposure to electromagnetic fields and childhood leukaemia: A meta-analysis. *Bulletin of the World Health Organization, 77*, 906-915.

Anonychuk, A. M., Tricco, A. C., Bauch, C. T., Pham, B., Gilca, V., Duval, B., . . . Krahn, M. (2008). Cost-Effectiveness analyses of hepatitis A vaccine: A systematic review to explore the effect of methodological quality on the economic attractiveness of vaccination strategies. *Pharmacoeconomics, 26*(1), 17-32.

Ariens, G. A., van Mechelen, W., Bongers, P. M., Bouter, L. M., & van der Wal, G. (2000). Physical risk factors for neck pain. *Scandinavian Journal of Work, Environment & Health, 26*, 7-19.

Armstrong, R., Waters, E., Jackson, N., Oliver, S., Popay, J., Shepherd, J., … Thomas H. (2007). *Guidelines for Systematic reviews of health promotion and public health interventions. Version 2.* Melbourne University, Australia. Retrieved from http://ph.cochrane.org/sites/ph.cochrane.org/files/uploads/Guidelines%20HP_PH%20reviews.pdf.

Arroll, B., Schechter, M. T., & Sheps, S. B. (1988). The assessment of diagnostic tests: A comparison of medical literature in 1982 and 1985. *Journal of General Internal Medicine, 3*, 443-447.

Ashrafian, H., & Athanasiou, T. (2010). How to read a paper. In T. Athanasiou (Ed.), *Key topics in surgical research and methodology* (pp. 545-555). Berlin-Heidelberg, Germany: Springer-Verlag.

Auperin, A., Pignon, J. P., & Poynard, T. (1997). Review article: Critical review of meta-analyses of randomized clinical trials in hepatogastroenterology. *Alimentary Pharmacology & Therapeutics, 11*, 215-225.

Baker, T. B., Gustafson, D. H., Shaw, B., Hawkins, R., Pingree, S., Roberts, L., & Strecher, V. (2010). Relevance of CONSORT reporting criteria for research on eHealth interventions. *Patient Education and Counseling, 81*(Suppl.), 77-86.

Balshem, H., Helfand, M., Schünemann, H. J., Oxman, A. D., Kunz, R., Brozek, J., . . . Guyatt, G. H. (2011). GRADE guidelines: 3. Rating the quality of evidence. *Journal of Clinical Epidemiology, 64*, 401-406.

Banovac, F., Buckley, D. C., Kuo, W. T., Lough, D. M., Martin, L. G., Millward, S. F., . . . Technology Assessment Committee of the Society of Interventional Radiology. (2010). Reporting standards for endovascular treatment of pulmonary embolism. *Journal of Vascular and Interventional Radiology, 21*(1), 44-53.

Baranowsky, J., Klose, P., Musial, F., Haeuser, W., Dobos, G., & Langhorst, J. (2009). Qualitative systemic review of randomized controlled trials on complementary and alternative medicine treatments in fibromyalgia. *Rheumatology International, 30*(1), 1-21.

Barnes, D. E., & Bero, L. A. (1998). Why review articles on the health effects of passive smoking reach different conclusions. *JAMA, 279*, 1566-1570.

Barratt, A., Irwig, L., Glasziou, P., Cumming, R. G., Raffle, A., Hicks, N., . . . Guyatt, G. H. (1999). Users'guides to the medical literature: XVII. How to use guidelines and recommendations about screening. *JAMA, 281*(21), 2029-2034.

Bartlett, J. G., Breiman, R. F., Mandell, L. A., & File, T. M. J. (1998). Community-acquired pneumonia in adults: Guidelines for management. *Clinical Infectious Diseases, 26*, 811-838.

Beccari, M. N., & Oliveira, T. L. (2011). A philosophical approach about user experience methodology. In A. Marcus (Ed.), *Design, user experience, and usability. Theory, methods, tools and practice lecture notes in computer science, Vol. 6769* (pp. 13-22). Orlando, FL: First International Conference, DUXU 2011, Held as Part of HCI International 2011, July 9-14, 2011, Proceedings, Part I.

Begg, C., Cho, M., Eastwood, S., Horton, R., Moher, D., Olkin, I., . . . Stroup, D. F. (1996). Improving the quality of reporting of randomized controlled trials. The CONSORT statement. *JAMA, 276*(8), 637-639.

Beller, E. M., Glasziou, P. P., Altman, D. G., Hopewell, S., Bastian, H., Chalmers, I., . . . Tovey, D. (2013). PRISMA for abstracts: Reporting systematic reviews in journal and conference abstracts. *PLOS Medicine, 10*(4). doi:10.1371/journal.pmed.1001419

Benchimol, E. I., Manuel, D. G., To, T., Griffiths, A. M., Rabeneck, L., & Guttmann, A. (2011). Development and use of reporting guidelines for assessing the quality of validation studies of health administrative data. *Journal of Clinical Epidemiology, 64*(8), 821-829.

Berger, A. M., Wielgus, K. K., Young-McCaughan, S., Fischer, P., Farr, L., & Lee, K. A. (2008). Methodological challenges when using actigraphy in research. *Journal of Pain and Symptom Management, 36*(2), 191-199.

Berger, M. L., Mamdani, M., Atkins, D., & Johnson, M. L. (2009). Good research practices for comparative effectiveness research: Defining, reporting and interpreting nonrandomized studies of treatment effects using secondary data sources: The ISPOR good research practices for retrospective database analysis task force report - Part I. *Value in Health, 12*(8), 1044-1052.

Berwick, D. M. (2008). The science of improvement. *JAMA, 299*(10), 1182-1184.

Bezerra, M. A., Lemos, A., Lira, K. D., Silveira, P. V., Coutinho, M. P., & E. Moraes, S. R. (2012). Does aerobic exercise training promote changes in structural and biomechanical properties of the tendons in experimental animals? A systematic review. *Biology of Sport, 29*(4), 249-254.

Blignault, I., & Ritchie, J. (2009). Revealing the wood and the trees: Reporting qualitative research. *Health promotion journal of Australia, 20*(2), 140-145.

Boccia, S., De Feo, E., Gallí, P., Gianfagna, F., Amore, R., & Ricciardi, G. (2010). A systematic review evaluating the methodological aspects of meta-analyses of genetic association studies in cancer research. *European Journal of Epidemiology, 25*, 765–775.

Bookman, E., Langehorne, A., Eckfeldt, J., Glass, K., Jarvik, G., Klag, M., . . . Luepker, R. V. (2006). Reporting genetic results in research studies: summary and recommendations of an NHLBI working group. *American Journal of Medical Genetics Part A, 140*(10), 1033-1040.

Booth, A. (2006). "Brimful of STARLITE": Toward standards for reporting literature searches. *Journal of the Medical Library Association, 94*(4), 421-429.

Bosch, F., Guardiola, E., & Esteve Foundation Workshop Group 2002 (2003). Lista de comprobación (checklist) abreviada para la evaluación de artículos de investigación biomédica básica [Abridged checklist to evaluate papers about basic biomedical research]). *Medicina Clínica, 121*(6), 228-230.

Bossuyt, P. M., Reitsma, J. B., Bruns, D. E., Gatsonis, C. A., Glasziou, P. P., Irwig, L. M., . . . de Vet, H. C. W. (2003). Towards complete and accurate reporting of studies of diagnostic accuracy: The STARD initiative. *Radiology, 226*(1), 24-28.

Bousquet, P. J., Brozek, J., Bachert, C., Bieber, T., Bonini, S., Burney, P., . . . Bousquet, J. (2009). The CONSORT statement checklist in allergen-specific immunotherapy: A GA2LEN paper. *Allergy, 64*(12), 1737-1745.

Boutron, I., Moher, D., Altman, D. G., Schulz, K., & Ravaud, P. (2008). Methods and processes of the CONSORT Group: Example of an extension for trials assessing nonpharmacologic treatments. *Annals of Internal Medicine, 148*(4), 60-67.

Bow, S., Klassen, J., Chisholm, A., Tjosvold, L., Thomson, D., Klassen, T. P., . . . Hartling, L. (2010). A descriptive analysis of child-relevant systematic reviews in the Cochrane Database of Systematic Reviews. *BMC Pediatrics, 10*. Retrieved from <http://www.biomedcentral.com/1471-2431/10/34>

Braden, J. B., Feng, X., & Won, D. H. (2011). Waste sites and property values: A meta-analysis. *Environmental and Resource Economics, 50*, 175–201.

Braithwaite, D., Emery, J., Walter, F., Prevost, A. T., & Sutton, S. (2004). Psychological impact of genetic counseling for familial cancer: A systematic review and meta-analysis. *Journal of the National Cancer Institute, 96*(2), 122-133.

Brazma, A., Hingamp, P., Quackenbush, J., Sherlock, G., Spellman, P., Stoeckert, C., . . . , Vingron, M. (2001). Minimum information about a microarray experiment (MIAME)-toward standards for microarray data. *Nature Genetics, 29*(4), 365-371.

Bril, V., Allenby, K., Midroni, G., O'Connor, P. W., & Vajsar, J. (1999). IGIV in neurology––evidence and recommendations. *Canadian Journal of Neurological Sciences, 26*, 139-152.

Briss, P. A., Zaza, S., Pappaioanou, M., Fielding, J., Wright-De Agüero, L., Truman, B. I., . . . The Task Force on community Preventive Services (2000). Developing an evidence-based guide to community preventive services––methods. *American Journal of Preventive Medicine, 18*(1), 35-43.

Brown, D. B., Gould, J. E., Gervais, D. A., Goldberg, S. N., Murthy, R., Millward, S. F., . . . Society of Interventional Radiology Technology Assessment Committee and the International Working Group on Image-Guided Tumor Ablation (2009). Transcatheter therapy for hepatic malignancy: Standardization of terminology and reporting criteria. *Journal of Vascular and Interventional Radiology, 20*(Suppl. 7), 425-434.

Brown, P., Brunnhuber, K., Chalkidou, K., Chalmers, I., Clarke, M., Fenton, M., . . . Young, P. (2006). How to formulate research recommendations. *BMJ, 333*(7572), 804-806.

Brown, S. A. (1991). Measurement of quality of primary studies for meta-analysis. *Nursing Research, 40*(6), 352-355.

Brozek, J., Oxman, A., & Schünemann, H. (2008). GRADEpro. Version 3.2 for Windows.

Brunetti, M., Shemilt, I., Pregno, S., Vale, L., Oxman, A. D., Lord, J., . . . Schünemann, H. J. (2013). GRADE guidelines: 10. Considering resource use and rating the quality of economic evidence. *Journal of Clinical Epidemiology, 66*, 140-150.

Bruns, D. E. (1997). Reporting diagnostic accuracy. *Clinical Chemistry, 43*(11), 2211-2212.

Bucher, H. C., Guyatt, G. H., Cook, D. J., Holbrook, A., & McAlister, F. A. (1999). Users' guides to the medical literature: XIX. Applying clinical trial results. A. How to use an article measuring the effect of an intervention on surrogate end points. *JAMA,* *282*(8), 771-778.

Burns, M. J., & O’Connor, A. M. (2008). Assessment of methodological quality and sources of variation in the magnitude of vaccine efficacy: A systematic review of studies from 1960 to 2005 reporting immunization with Moraxella bovis vaccines in young cattle. *Vaccine, 26*, 144–152.

Burton, A., & Altman, D. G. (2004). Missing covariate data within cancer prognostic studies: A review of current reporting and proposed guidelines. *British Journal of Cancer, 91*(1), 4-8.

Burton, J., Darbes, L. A., & Operario, D. (2010). Couples-Focused Behavioral Interventions for Prevention of HIV: Systematic Review of the State of Evidence. *AIDS and Behavior, 14*(1), 1-10.

Callstrom, M. R., York, J. D., Gaba, R. C., Gemmete, J. J., Gervais, D. A., Millward, S. F., . . . Cardella, J. F. (2009). Research reporting standards for image-guided ablation of bone and soft tissue tumors. *Journal of Vascular and Interventional Radiology, 20*(12), 1527-1540.

Calvert, M., Blazeby, J., Altman, D. G., Revicki, D. A., Moher, D., Brundage, M. D., & CONSORT PRO Group (2013). Reporting of patient-reported outcomes in randomized trials: The CONSORT PRO extension. *JAMA, 309*(8), 814-822.

Campbell, M. K., Elbourne, D. R., & Altman, D. G. (2004). CONSORT statement: Extension to cluster randomised trials. *BMJ, 328*, 702-708.

Campbell, M. K., Piaggio, G., Elbourne, D. R., Altman, D. G., & for the Consort Group. (2012). Consort 2010 statement: Extension to cluster randomised trials. *BMJ, 345*. doi:[10.1136/bmj.e5661](http://dx.doi.org/10.1136/bmj.e5661)

Canadian Task Force on the Periodic Health Examination (1979). The periodic health examination. *Canadian Medical Association Journal, 121*, 1193-1254.

Carayol, M., Grosclaude, P., & Delpierre, C. (2010). Prospective studies of dietary alpha-linolenic acid intake and prostate cancer risk: a meta-analysis. *Cancer Causes Control, 21*(3), 347-355.

Carlson, D. (2011). Trends and innovations in public policy analysis. *Policy Studies Journal, 39*(1), 13-25.

Carruthers, S. G., Larochelle, P., Haynes, R. B., Petrasovits, A., & Schiffrin, E. L. (1993). Report of the Canadian Hypertension Society Consensus Conference: 1. Introduction. *Canadian Medical Assocition Journal, 149*, 289-293.

Cecile, A., Janssens, J. W., Ioannidis, J. P. A., van Duijn, C. M., Little, J., Khoury, M. J., & Group, R. T. G. (2011). Strengthening the reporting of genetic risk prediction studies: The GRIPS Statement. *PLoS Medicine 8*(3). doi:10.1371/journal.pmed.1000420

Cerin, E., Barnett, A., & Baranowski, T. (2009). Testing theories of dietary behavior change in youth using the mediating variable model with intervention programs. *Journal of Nutrition Education and Behavior, 41*, 309-318.

Chacón, S., Sanduvete, S., Portell, M., & Anguera, M. T. (2013). Reporting a program evaluation: Needs, program plan, intervention, and decisions. *International Journal of Clinical and Health Psychology, 13*(1), 58-66*.*

Chalmers, T. C., Smith, H. J., Blackburn, B., Silverman, B., Schroeder, B., Reitman, D., & Ambroz, A. (1981). A method for assessing the quality of a randomized control trial. *Controlled Clinical Trials, 2*(1), 31-49.

Chan, A.-W., Tetzlaff, J. M., Altman, D. G., Laupacis, A., Gøtzsche, P. C., Krleža-Jerić, K., . . . Moher, D. (2013). SPIRIT 2013 Statement: Defining standard protocol items for clinical trials. *Annals of Internal Medicine, 158*(3), 200-207.

Chang, S., Vogelbaum, M., Lang, F. F., Haines, S., Kunwar, S., Chiocca, E. A., . . . American Association of Neurological Surgeons and Congress of Neurological Surgeons (AANS/CNS) (2007). GNOSIS: Guidelines for neuro-oncology: Standards for investigational studies - reporting of surgically based therapeutic clinical trials. *Journal of Neuro-Oncology, 82*(2), 211-220.

Chang, S. M., Reynolds, S. L., Butowski, N., Lamborn, K. R., Buckner, J. C., Kaplan, R. S., & Bigner, D. D. (2005). GNOSIS: Guidelines for neuro-oncology: Standards for investigational studies-reporting of phase 1 and phase 2 clinical trials. *Neuro-Oncology, 7*(4), 425-434.

Chávez, N. (2011). Supplemental nutrition programs during pregnancy and the early postnatal period. In A. Handler, J. Kennelly, & N. Peacock (Eds.), *Reducing racial/ethnic disparities in reproductive and perinatal outcomes. The evidence from population-based interventions* (pp. 329-367). New York-Dordrecht-Heidelberg-London: Springer.

Cheng, T., Pan, X. Y., Mao, X., Zhang, G. Y., & Zhang, X. L. (2012). Little clinical advantage of computer-assisted navigation over conventional instrumentation in primary total knee arthroplasty at early follow-up. *The Knee, 19*, 237–245.

Cheson, B. D., Bennett, J. M., Kopecky, K. J., Buchner, T., Willman, C. L., Estey, E. H., . . . International Working Group for Diagnosis, Standardization of Response Criteria, Treatment Outcomes, and Reporting Standards for Therapeutic Trials in Acute Myeloid Leukemia. (2003). Revised recommendations of the International Working Group for Diagnosis, Standardization of Response Criteria, Treatment Outcomes, and Reporting Standards for Therapeutic Trials in Acute Myeloid Leukemia. *Journal of Clinical Oncology, 21*(24), 4642-4649.

Chesson, A. L. J., Wise, M., Davila, D., Johnson, S., Littner, M., Anderson, W. M., . . . Rafecas, J. (1999). Practice parameters for the treatment of restless legs syndrome and periodic limb movement disorder. *Sleep, 22*(7), 961-968.

Chiou, C. H., Hay, J. W., Wallace, J. F., Bloom, B. S., Neumann, P. J., Sullivan, S. D., . . . Ofman, J. J. (2003). Development and validation of a grading system for the quality of cost-effectiveness studies. *Medical Care, 41*, 32-44.

Chokshi, D. A., Avorn, J., & Kesselheim, A. S. (2010). Designing Comparative Effectiveness Research On Prescription Drugs: Lesson from the clinical trial literature. *Health Affairs, 29*(10), 1842-1848.

Clark, J. P. (2003). How to peer review a qualitative manuscript. In F. Godlee, & T. Jefferson (Eds.), *Peer Review in Health, 2^nd^* *ed.*, (pp. 219-235). London, UK: BMJ Books.

Clark, T. W., Millward, S. F., Gervais, D. A., Goldberg, S. N., Grassi, C. J., Kinney, T. B., . . . Cardella, J. F. (2009). Reporting standards for percutaneous thermal ablation of renal cell carcinoma. *Journal of Vascular and Interventional Radiology, 20*(Suppl. 7), 409-416.

Clarke, M. J., & Stewart, L. A. (1994). Obtaining data from randomised controlled trials: How much do we need for reliable and informative meta-analyses? *BMJ, 309*, 1007-10.

Classen, S., Winter, S., Awadzi, K. D., Garvan, C. W., Lopez, E. D. S., & Sundaram, S. (2008). Psychometric testing of SPIDER: Data capture tool for systematic literature reviews. *The American Journal of Occupational Therapy, 62*(3), 335–348.

Cluzeau, F. A., Burgers, J., Brouwers, M., Grol, R., Makela, M., Littlejohns, P., . . . for the AGREE Collaboration (2003). Development and validation of an international appraisal instrument for assessing the quality of clinical practice guidelines: The AGREE project. *Quality and Safety in Health Care, 12*, 18-23.

Cluzeau, F. A., Littlejohns, P., Grimshaw, J. M., Feder, G., & E., M. S. (1999). Development and application of a generic methodology to assess the quality of clinical guidelines. *International Journal for Quality in Health Care, 21*(1), 21-28.

Cobo, J., Sendino, R., Fabregate, M., Cimarra, I., Martínez, P., & Deglané, R. (2010). Predictors of outcome after decompressive lumbar surgery and instrumented posterolateral fusion. E*uropean Spine Journal, 19*(11), 1841-1848.

Colbert, A. P., Spaulding, K., Larsen, A., Ahn, A. C., & Cutro, J. A. (2011). Electrodermal activity at acupoints: Literature review and recommendations for reporting clinical trials. *Journal of Acupuncte Meridian Studies, 4*(1), 5-13.

Coleman, B. D., Khan, K. M., Maffulli, N., Cook, J. L., & Wark, J. D. (2000). Studies of surgical outcome after patellar tendinopathy: clinical significance of methodological deficiencies and guidelines for future studies. *Scandinavian Journal of Medicine & Science in Sports, 10*(1), 2-11.

Coleman, S., Gorecki, C., Nelson, E. A., Closs, S. J., Defloor, T., Halfens, R., . . . Nixon, J. (2013). Patient risk factors for pressure ulcer development: Systematic review. *International Journal of Nursing Studies, 50*, 974–1003.

Comenzo, R. L., Reece, D., Palladini, G., Seldin, D., Sanchorawala, V., Landau, H., . . . Merlini, G. (2012). Consensus guidelines for the conduct and reporting of clinical trials in systemic light-chain amyloidosis. *Leukemia, 26*(11), 2317-2325.

Conn, V. S., Hafdahl, A. R., & Mehr, D. R. (2011). Interventions to increase physical activity among healthy adults: Meta-analysis of outcomes. *American Journal of Public Health, 101*(4), 751-758.

Conway, J. M., & Lance, C. E. (2010). What reviewers should expect from authors regarding common method bias in organizational research. *Journal of Business and Psychology, 25*(3), 325-334

Cook, D., Beckman, T., & Bordage, G. (2007). Quality of reporting of experimental studies in medical education: A systematic review. *Medical Education, 41*, 737-745.

Cook, D. J., Guyatt, G. H., Laupacis, A., & Sackett, D. L. (1992). Rules of evidence and clinical recommendations on the use of antithrombotic agents. *Chest, 102*, 305-311.

Cook, D. J., Sackett, D. L., & Spitzer, W. O. (1995). Methodologic guidelines for systematic reviews of randomized control trials in health care from the Potsdam Consultation on Meta-Analysis. *Journal of Clinical Epidemiology, 48*, 167-171.

Cornelius, L. R., van der Klink, J. J. L., Groothoff, J. W., & Brouwer, S. (2011). Prognostic factors of long term disability due to mental disorders: A systematic review. *Journal of Occupational Rehabilitation, 21*, 259–274.

Cornelius, V. R., Perrio, M. J., Shakir, S. A. W., & Smith, L. A. (2009). Systematic reviews of adverse effects of drug interventions: A survey of their conduct and reporting quality. *Pharmacoepidemiology and Drug Safety, 18*(12), 1223-1231.

Corrao, G., Bagnardi, V., Zambon, A., & Arico, S. (1999). Exploring the dose-response relationship

between alcohol consumption and the risk of several alcohol-related conditions: A metaanalysis. *Addiction, 94*, 1551-1573.

Coull, G., & Morris, P. G. (2011). The clinical effectiveness of CBT-based guided self-help interventions for anxiety and depressive disorders: A systematic review. *Psychological Medicine, 41*, 2239–2252.

Courtney, J. (2008). Do monitoring and evaluation tools, designed to measure the improvement in the quality of primary education, constrain or enhance educational development?  *International Journal of Educational Development, 28*(5), 546–559.

Craig, P., Dieppe, P., Macintyre, S., Michie, S., Nazareth, I., & Petticrew, M. (2013). Developing and evaluating complex interventions: New guidance. *International Journal of Nursing Studies, 50*(5), 587-592. Retrieved from <http://www.mrc.ac.uk/documents/pdf/complex-interventions-guidance/>

Crawford, F., Anandan, C., Chappell, F. M., Murray, G. D., Price, J. F., Sheikh, A., . . . Tierney, J. (2013). Protocol for a systematic review and individual patient data meta-analysis of prognostic factors of foot ulceration in people with diabetes: the international research collaboration for the prediction of diabetic foot ulcerations (PODUS). *BMC Medical Research Methodology, 13*. Retrieved from <http://www.biomedcentral.com/1471-2288/13/22>

Critical appraisal skills programme (CASP, 2013). Making sense of evidence. Oxford: Cohort Studies Critical Appraisal Skills Public Health Resource Unit. Retrieved from <http://www.caspuk.net/>.

Currow, D. C., Tieman, J. J., Greene, A., Zafar, S. Y., Wheeler, J. L., & Abernethy, A. P. (2012). Refining a checklist for reporting patient populations and service characteristics in hospice and palliative care research. *Journal of Pain and Symptom Management, 43*(5), 902-910.

Dans, A. L., Dans, L. F., Guyatt, G. H., & Richard, S. (1998). Users' guides to the medical literature: XIV. How to decide on the applicability of clinical trial results to your patient. *JAMA, 279*, 545-549.

Darcourt, J., Booij, J., Tatsch, K., Varrone, A., Vander Borght, T., Kapucu, O. L., . . . Van Laere, K. (2010). EANM procedure guidelines for brain neurotransmission SPECT using I-labelled dopamine transporter ligands, version 2. *European Journal of Nuclear Medicine and Molecular Imaging, 37*(2), 443-450.

Davidoff, F., Batalden, P., Stevens, D., Ogrinc, G., & Mooney, S. (2008). Publication guidelines for quality improvement in health care: Evolution of the SQUIRE project. *Quality & Safety in Health Care, 17*(Suppl. 1), 3-9.

Davidson, K. W., Goldstein, M., Kaplan, R. M., Kaufmann, P. G., Knatterud, G. L., Orleans, C. T., . . . Whitlock, E. P. (2003). Evidence-based behavioral medicine: What is it and how do we achieve it? *Annals of Behavioral Medicine, 26*(3), 161-171.

Davis, J. C., Robertson, M. C., Comans, T., & Scuffham, P. A. (2011). Guidelines for conducting and reporting economic evaluation of fall prevention strategies. *Osteoporosis International, 22*(9), 2449-2459.

De Keizer, N. F., Talmon, J., Ammenwerth, E., Brender, J., Rigby, M., & Nykanen, P. (2012). Systematic prioritization of the STARE-HI reporting items. An application to short conference papers on health informatics evaluation. *Methods of Information in Medicine, 51*(2), 104-111.

De Vet, H. C. W., de Bie, R. A., van der Heijden, G. J. M. G., Verhagen, A. P., Sijpkes, P., & Kipschild, P. G. (1997). Systematic reviews on the basis of methodological criteria. *Physiotherapy, 83*(6), 284-289.

De Vries, J. S., Krips, R., Sierevelt, I. N., & Blankevoort, L. (2006). Interventions for treating chronic ankle instability. *Cochrane Database of Systematic Reviews, 4*. doi: 10.1002/14651858

Dean, M. E., Coulter, M. K., Fisher, P., Jobst, K., & Walach, H. (2006). Reporting data on homeopathic treatments (RedHot): A supplement to CONSORT. *Forsch Komplementmed, 13*(6), 368-371.

Dechartres, A., Boutron, I., Roy, C., & Ravaud, P. (2009). Inadequate planning and reporting of adjudication committees in clinical trials: Recommendation proposal. *Journal of Clinical Epidemiology, 62*(7), 695-702.

Declercq, E., Barger, M., & Weiss, J. (2011). Contemporary childbirth in the United States: Interventions and disparities. In A. Handler, J. Kennelly, & N. Peacock (Eds.), *Reducing Racial/Ethnic Disparities in Reproductive and Perinatal Outcomes: The Evidence from Population-Based Interventions* (pp. 401-427). New York, NY: Springer Science.

Delgado-Bolton, R. C., Fernández-Pérez, C., González-Maté, A., & Carreras, J. L. (2003). Meta-analysis of the performance of 18F-FDG PET in primary tumor detection in unknown primary tumors. *The Journal of Nuclear Medicine, 44*, 1301–1314.

Delgado-Rodrigues, M. (2006). Systematic reviews of meta-analyses: Applications and limitations. *Journal of Epidemiology and Community Health, 60*, 90-92.

Dennis, C. -L., & Dowswell, T. (2013). Psychosocial and psychological interventions for preventing postpartum depression. *Cochrane Database of Systematic Reviews, 2*. doi: 10.1002/14651858.CD001134.pub3.

Department of Clinical Epidemiology and Biostatistics, M. U. H. S. C. (1981). How to read clinical journals: IV. To determine etiology or causation. *Canadian Medical Association Journal, 124*, 985-990.

Des Jarlais, D. C., Lyles, C., Crepaz, N., & the TREND group (2004). Improving the reporting quality of nonrandomized evaluations of behavioral and public health interventions: The TREND statement. *American Journal of Public Health, 94*(3), 361-366.

Desmidt, S., Prinzie, A., & Decramer, A. (2011). Looking for the value of misión statements: A meta-analysis of 20 years of research. *Management Decision, 49*(3), 468-483.

Desplenter, F. A. M., Simoens, S., & Laekeman, G. (2006). The impact of informing psychiatric patients about their medication: a systematic review. *Pharmacy World & Science, 28*(6), 329-341.

Detsky, A. S., Naylor, D., O’rourke, K., Mcgeer, A. J., & L’abbé, K. A. (1992). Incorporating variations in the quality of individual randomized trials into meta-analysis. *Journal of Clinical Epidemiology, 45*(3), 255-265.

DeVivo, M. J., Biering-Sørensen, F., New, P., & Chen, Y. (2011). Standardization of data analysis and reporting of results from the International Spinal Cord Injury Core Data Set. *Spinal Cord, 49*(5), 596-599.

Dickersin, K., Scherer, R., & Lefebvre, C. (1994). Identifying relevant studies for systematic reviews. *BMJ, 309*, 1286-1291.

Dixon, W. G., Carmona, L., Finckh, A., Hetland, M. L., Kvien, T. K., Landewe, R., . . . Askling, J. (2010). EULAR points to consider when establishing, analysing and reporting safety data of biologics registers in rheumatology. *Annals of the Rheumatic Diseases, 69*(9), 1596-1602.

Dobbins, M., DeCorby, K., & Twiddy, T. (2004). A knowledge transfer strategy for public health decision makers. *Worldviews on Evidence-Based Nursing, 1*, 120-128.

Docherty, M., & Smith, R. (1999). The case for structuring the discussion of scientific papers. *BMJ, 318*(7193), 1224-1225.

Donahue, S. P., Arnold, R. W., & Ruben, J. B. (2003). Preschool vision screening: What should we be detecting and how should we report it? Uniform guidelines for reporting results of preschool vision screening studies. *Journal of AAPOS, 7*(5), 314-316.

Donegan, S., Williamson, P., Gamble, C., & Tudur-Smith, C. (2010). Indirect comparisons: A review of reporting and methodological quality. *PLoS ONE, 5*(11). doi:10.1371/journal.pone.0011054

Downing, A., Rudge, G., Cheng, Y., Tu, Y., Keen, J., & Gilthorpe, M. S. (2007). Do the UK government’s new Quality and Outcomes Framework (QOF) scores adequately measure primary care performance? A cross-sectional survey of routine healthcare data. *BMC Health Services Research, 7*:166. doi:10.1186/1472-6963-7-166

Downs, S. H., & Black, N. (1998). The feasibility of creating a checklist for the assessment of the methodological quality both of randomised and non-randomised studies of health care interventions.*Journal of Epidemiology and Community Health, 52*(6), 377-384.

Drummond, M. F., Jefferson, T. O., & The BMJ Economic Evaluation Working Party Economic Evaluation Working Party (1996). Guidelines for authors and peer reviewers of economic submissions to the BMJ. *BMJ, 313*(7052), 275-283.

Drummond, M. F., Manca, A., & Sculpher, M. (2005). Increasing the generalizability of economic evaluations: recommendations for the design, analysis, and reporting of studies. *International Journal of Technology Assessment in Health Care, 21*(2), 165-171.

Drummond, M. F., Richardson, W. S., O'Brien, B. J., Levine, M., & Heyland, D. (1997). Users' guides to the medical literature. XIII. How to use an article on economic analysis of clinical practice. A. Are the results of the study valid? *JAMA, 277*(19), 1552-1557.

Dupuy, A., & Simon, R. M. (2007). Critical review of published microarray studies for cancer outcome and guidelines on statistical analysis and reporting. *Journal of the National Cancer Institute, 99*, 147-157.

Dyer, M. T. D., Goldsmith, K. A., Sharples, L. S., & Buxton, M. J. (2010). A review of health utilities using the EQ-5D in studies of cardiovascular disease. *Health and Quality of Life Outcomes, 8*. Retrieved from <http://www.hqlo.com/content/8/1/13>

Dzewaltowski, D. A., Estabrooks, P. A., Klesges, L. M., & Glasgow, R. E. (2004). TREND: An important step, but not enough. *American Journal of Public Health, 94*(9), 1474.

Easterbrook, P. J., Berlin, J. A., Gopalan, R., & Matthews, D. R. (1991). Publication bias in clinical research. *Lancet, 337*, 867-872.

Education Group for Guidelines on Evaluation (1999). Guidelines for evaluating papers on educational interventions. *BMJ, 318,* 1265-1267.

Effective Public Health Practice Project (1998). *Quality assessment tool for quantitative studies*. Retrieved from <http://www.ephpp.ca/Tools.html>

Efficace, F., Bottomley, A., Osoba, D., Gotay, C., Flechtner, H., D'haese, S., & Zurlo, A. (2003). Beyond the development of health-related quality-of-life (HRQOL) measures: a checklist for evaluating HRQOL outcomes in cancer clinical trials--does HRQOL evaluation in prostate cancer research inform clinical decision making? *Journal of Clinical Oncology, 21*(18), 3502-3511.

Ehliasson, K. (2008). Futures studies as social science: An analytic scheme and a case study. *Futures, 40*(5), 489- 502.

Eken, C. (2015). Critical reappraisal of intravenous metoclopramide in migraine attack: A systematic review and meta-analysis. *American Journal of Emergency Medicine, 33*(3), 331-337.

El Baz, N., Middel, B., van Dijk, J. P., Oosterhof, A., Boonstra, P. W., & Reijneveld, S. A. (2007). Are the outcomes of clinical pathways evidencebased? A critical appraisal of clinical pathway evaluation research. *Journal of Evaluation in Clinical Practice, 13*, 920–929.

Elliott, R., Fischer, C. T., & Rennie, D. L. (1999). Evolving guidelines for publication of qualitative research studies in psychology and related fields. *British Journal of Clinical Psychology, 38*(3), 215-229.

Erford, B. T., Erford, B. M., Lattanzi, G., Weller, J., Schein, H., Wolf, E., . . . Peacock, E. (2011). Counseling outcomes from 1990 to 2008 for school-age youth with depression: A meta-Analysis. *Journal of Counseling and Development, 89*(4), 439-457.

Esmonde, L., McDonnell, A., Ball, C., Waskett, C., Morgan, R., Rashidian, A., . . . Harvey, S. (2006). Investigating the effectiveness of critical care outreach services: A systematic review. *Intensive Care Medicine, 32*(11), 1713-1721.

Espada, J. P., Gonzálvez, M. T., Orgilés, M., Lloret, D., & Guillén-Riquelme, A. (2015). Meta-analysis of the effectiveness of school substance abuse prevention programs in Spain. *Psicothema, 27*(1), 5-12.

Evaluation and Public Health Technologies Agency (1999). *Guía para la elaboración de informes de evaluación de tecnologías sanitarias [Guide to the development of evaluation reports for public health technologies].* Madrid: AETS – Health Institute “Carlos III”. Ministry of Health and Consumption.

Evans, W. K., Newman, T., Graham, I., Rusthoven, J. J., Logan, D., Shepherd, F. A., & Chamberlain, D. (1997). Lung cancer practice guidelines: Lessons learned and issues addressed by the Ontario Lung Cancer Disease Site Group. *Journal of Clinical Oncology, 15*(9), 3049-3059.

Eysenbach, G. (2004). Improving the quality of Web surveys: the Checklist for Reporting Results of Internet E-Surveys (CHERRIES). *Journal of Medical Internet Research, 6*(3). doi:10.2196/jmir.6.3.e34

Eysenbach, G. (2011). CONSORT-EHEALTH: improving and standardizing evaluation reports of Web-based and mobile health interventions. *Journal of Medical Internet Research, 13*(4). doi: 10.2196/jmir.1923

Faggion, C. M., Listl, S., & Alarcón, M. A. (2015). Is the evaluation of risk of bias in periodontology and implantdentistry comprehensive? A systematic review. *Journal of Clinical Periodontology, 42*, 488–494. doi:10.1111/jcpe.12394

Falkingham, M., Abdelhamid, A., Curtis, P., Fairweather-Tait, S., Dye, L., & Hooper, L. (2010). The effects of oral iron supplementation on cognition in older children and adults: a systematic review and meta-analysis. *Nutrition Journal, 9*. doi: 10.1186/1475-2891-9-4.

Farrar, A. (2009). *The application of research synthesis methods for evaluating primary research on Salmonella in broiler chickens*. University of Guelph, Guelph, Canada. Retrieved from http://www.collectionscanada.gc.ca/obj/thesescanada/vol2/002/MR56718.PDF

Farrington, D. P. (2003). Methodological quality standards for evaluation research. *The Annals of the American Academy, 587*, 49-58.

Feneck, R., Kneeshaw, J., Fox, K., Bettex, D., Erb, J., Flaschkampf, F., . . . European Association of Echocardiography (EAE) (2010). Recommendations for reporting perioperative transoesophageal echo studies. *European Journal Echocardiography, 11*(5), 387-393.

Fernandes, R. M., van der Lee, J. H., & Offringa, M. (2009). A systematic review of the reporting of Data Monitoring Committees' roles, interim analysis and early termination in pediatric clinical trials. *BMC Pediatrics, 9*. Retrieved from <http://www.biomedcentral.com/1471-2431/9/77>

Fernandez-de-las-Peñas, C., Alonso-Blanco, C., San-Roman, J., & Miangolarra-Page, J. C. (2006). Methodological quality of randomized controlled trials of spinal manipulation and mobilization in tension-type headache, migraine, and cervicogenic headache. *Journal of Orthopaedic & Sports Physical Therapy, 36*, 160–169.

Field, N., Cohen, T., Struelens, M. J., Palm, D., Cookson, B., Glynn, J. R., . . . Abubakar, I. (2014). Strengthening the Reporting of Molecular Epidemiology for Infectious Diseases (STROME-ID): An extension of the STROBE statement. *The Lancet Infectious Diseases, 14*, 341–352.

Fisher, J., Cabral de Mello, M., Patel, V., Rahman, A., Tran, T., Holton, S., & Holmes, W. (2012). Prevalence and determinants of common perinatal mental disorders in women in low- and lower-middle-income countries: A systematic review. *Bulletin of the World Health Organization, 90*, 139–149.

Fisher, M., Feuerstein, G., Howells, D. W., Hurn, P. D., Kent, T. A., Savitz, S. I., . . . Group, S. (2009). Update of the stroke therapy academic industry roundtable preclinical recommendations. *Stroke, 40*(6), 2244-2250.

Flores, S. A., & Crepaz, N. (2004). Quality of study methods in individual- and group-level HIV intervention research: Critical reporting elements. *AIDS Education and Prevention, 16*(4), 341-352.

Ford, A. C., & Moayyedi, P. (2009). Redundant data in the meta-analysis on helicobacter pylori eradication. *Annals of Internal Medicine, 151*(7), 513-514.

Fox, C., Mignini, L., & Khan, K. S. (2006). Systematic reviews of research to assess causation: A guide to methods and application. *European Clinics in Obstetrics and Gynecology, 1*(4), 251-256.

Franche, R.-L., Cullen, K., Clarke, J., Irvin, E., Sinclair, S., Frank, J., & The Institute forWork & Health (IWH) Workplace-Based RTW Intervention Literature Review Research Team (2005). Workplace-based return-to-work interventions: A systematic review of the quantitative literature. *Journal of Occupational Rehabilitation, 15*(4), 607-631.

Friedenreich, C. M. (1993). Methods for pooled analyses of epidemiologic studies. *Epidemiology, 4*, 295-302.

Furlan, A. D., Pennick, V., Bombardier, C., van Tulder, M., EditorialBoard, & Cochrane Back ReviewGroup (2009). 2009 Updated method guidelines for systematic reviews in the Cochrane back review group. *Spine, 34*(18), 1929–1941.

Gagnier, J. J., Boon, H., Rochon, P., Moher, D., Barnes, J., Bombardier, C., & for the CONSORT Group. (2006). Reporting Randomized, Controlled Trials of Herbal Interventions: An Elaborated CONSORT Statement. *Annals of Internal Medicine, 144*(5), 364-367.

Gagnier, J. J., Kienle, G., Altman, D. A., Moher, D., Sox, H., Riley, D., & the CARE Group. (2013). The CARE guidelines: Consensus-based clinical case reporting guideline development. *BMJ Case Rep, 110*(37), 603-608.

Gallo, V., Egger, M., McCormack, V., Farmer, P. B., Ioannidis, J. P., Kirsch-Volders, M., . . . Vineis, P. (2012). STrengthening the Reporting of OBservational studies in Epidemiology - Molecular Epidemiology (STROBE-ME): An extension of the STROBE statement. *European Journal of Clinical Investigation, 42*(1), 1-16.

Gao, X.-L., & McGrath, C. (2011). A review on the oral health impacts of acculturation. *Journal of Immigrant and Minority Health, 13*, 202–213.

Garbutt, J. C., West, S. L., Carey, T. S., Lohr, K. N., & Crews, F. T. (1999). Pharmacological treatment of alcohol dependence: A review of the evidence. *JAMA, 281*, 1318-1325.

Gardner, I. A., Nielsen, S. S., Whittington, R. J., Collins, M. T., Bakker, D., Harris, B., . . . S., Eda. (2011). Consensus-based reporting standards for diagnostic test accuracy studies for paratuberculosis in ruminants. *Preventive Veterinary Medicine, 101*(1-2), 18-34.

Geerts, W. H., Pineo, G. F., Heit, J. A., Bergqvist, D., Lassen, M. R., Colwell, C. W., & Ray, J. G. (2008). Prevention of venous thromboembolism: The Seventh ACCP Conference on Antithrombotic and Thrombolytic Therapy. *Chest, 126*(Suppl. 3), 338–400.

Gehling, M., Hermann, B., & Tryba, M. (2011). Meta-analysis of dropout rates in randomized controlled clinical trials. *Der Schmerz, 25*, 296–305.

Gerber, A. J., Kocsis, J. H., Milrod, B. L., Roose, S. P., Barber, J. P., Thase, M. E., . . . Leon, A. C. (2011). A quality-based review of randomized controlled trials of psychodynamic psychotherapy. *American Journal of Psychiatry, 168*, 19-28.

Giacomini, M. K., & Cook, D. J. (2000a). Users' guides to the medical literature: XXIII. Qualitative research in health care A. Are the results of the study valid? Evidence-Based Medicine Working Group. *JAMA, 284*(3), 3357-3362.

Giacomini, M. K., & Cook, D. J. (2000b). Users' guides to the medical literature: XXIII. Qualitative research in health care B. What are the results and how do they help me care for my patients? Evidence-Based Medicine Working Group. *JAMA, 284*(4), 478-482.

Gilbody, S., Richards, D., Brealey, S., & Hewitt, C. (2007). Screening for depression in medical settings with the Patient Health Questionnaire (PHQ): A diagnostic meta-analysis. *Journal of General Internal Medicine, 22*(11), 1596-1602.

Glasgow, R. E., Klesges, L. M., Dzewaltowski, D. A., Bull, S. S., & Estabrooks, P. A. (2004). The future of health behavior change research: What is needed to improve translation of research into health promotion practice? *Annals of Behavioral Medicine, 27*(1), 3-12.

Goldgruber, J., & Ahrens, D. (2010). Effectiveness of workplace health promotion and primary prevention interventions: A review. *Journal of Public Health, 18*(1), 75-88.

Goodman, S. N., Berlin, J., Fletcher, S. W., & Fletcher, R. H. (1994). Manuscript quality before and after peer review and editing at Annals of Internal Medicine. *Annals of Internal Medicine, 121*, 11-21.

Goossens, G. A., Stas, M., & Jérôme, M. (2011). Systematic review: Malfunction of totally implantable venous access devices in cancer patients. *Supportive Care in Cancer, 19*(7), 883-898.

Gotzsche, P. C. (1989). Methodology and overt and hidden bias in reports of 196 double-blind trials of nonsteroidal antiinflammatory drugs in rheumatoid arthritis. *Controlled Clinical Trials, 10*, 31-56.

Goudas, L., Carr., D. B., Bloch, R., Balk, E., Ioannidis, J. P. A., Terrin, … Lau, J. (2001). *Management of cancer pain* (Evidence Report/Technology Assessment, 35). Rockville, MD: Agency for Health Care Policy and Research.

Gould, M. K., Maclean, C. C., Kuschner, W. G., Rydzak, C. E., & Owens, D. K. (2001). Accuracy of positron emission tomography for diagnosis of pulmonary nodules and mass lesions: A meta-analysis. *JAMA, 285*, 914–924.

Green, L. W., & Glasgow, R. E. (2006). Evaluating the relevance, generalization, and applicability of research: Issues in external validation and translation methodology. *Evaluation & the Health Professions, 29*, 126-153.

Greenhalgh, T. (1997). How to read a paper: Assessing the methodological quality of published papers. *BMJ, 315*, 305-308.

Greenland, S. (1994). Quality scores are useless and potentially misleading. *American Journal of Epidemiology, 140*(3), 300-301.

Grimes, D. A., & Schulz, K. F. (1996). Methodology citations and the quality of randomized controlled trials in obstetrics and gynecology. *American Journal of Obstetrics & Gynecology, 174*, 1312-1315.

Grimshaw, J., Eccles, M., Thomas, R., MacLennan, G., Ramsay, C. R., Fraser, C., & Vale, L. (2006). Toward evidence-based quality improvement. Evidence (and its limitations) of the effectiveness of guideline dissemination and implementation strategies 1966-1998. *The Journal of General Internal Medicine, 21*(Suppl. 2), 14-20.

Grimshaw, J., Thomas, R., MacLennan, G., Fraser, C., Ramsay, C. R., Vale, L., . . . Donaldson, C. (2004). Effectiveness and efficiency of guideline dissemination and implementation strategies. *Health Technology Assessment, 8*(6), 1-72.

Groenwold, R. H., Van Deursen, A. M., Hoes, A. W., & Hak, E. (2008). Poor quality of reporting confounding bias in observational intervention studies: A systematic review. *Annals of Epidemiology, 18*, 746–751.

Gross, P. A., Barrett, T. L., Dellinger, E. P., Krause, P. J., Martone, W. J., McGowan, J. E. J., . . . Wenzel, R. P. (1994). Purpose of quality standards for infectious diseases. *Clinical Infectious Diseases, 3*, 422-427.

Guo, R., Pittler, M. H., & Ernst, E. (2007). complementary medicine for treating or preventing influenza or influenza-like illness. *The American Journal of Medicine, 120*, 923-929.

Guyatt, G. H., Cook, D. J., Jaeschke, R., Pauker, S. J., & Schunemann, H. J. (2008). Grades of recommendation for antithrombotic agents. *Chest, 133*(6), 123-131.

Guyatt, G. H., Cook, D. J., Sackett, D. L., Eckman, M., & Pauker, S. (1998). Grades of recommendation for antithrombotic agents. *Chest, 114*(Suppl.), 441-444.

Guyatt, G. H., Haynes, R. B., Jaeschke, R. Z., Cook, D. J., Green, L., Naylor, C. D., . . . Richardson, W. S. (2000). Users' guides to the medical literature: XXV. Evidence-based medicine: Principles for applying the users' guides to patient care. *JAMA, 284*, 1290-1296.

Guyatt, G. H., Naylor, C. D., Juniper, E., Heyland, D. K., Jaeschke, R., & Cook, D. J. (1997). Users' guides to the medical literature. XII. How to use articles about health-related quality of life. *JAMA, 277*(15), 1232-1237.

Guyatt, G. H., Oxman, A. D., Kunz, R., Brozek, J., Alonso-Coello, P., Rind, D., . . . Schünemann, H. J. (2011). GRADE guidelines 6. Rating the quality of evidence – imprecision. *Journal of Clinical Epidemiology, 64*, 1283-1293.

Guyatt, G. H., Oxman, A. D., Kunz, R., Woodcock, J., Brozek, J., Helfand, M., Alonso-Coelloh, P., Falck-Ytter,Y., Jaeschke, R., Vist, G., Akl, E. A., Post, P. N., Norris, S., Meerpohl, J., Shukla, V. K., Nasser, M., & Schünemann, H. J. (2011). GRADE guidelines: 8. Rating the quality of evidenced in directness. *Journal of Clinical Epidemiology, 64*, 1303-1310.

Guyatt, G. H., Oxman, A. D., Kunz, R., Woodcock, J., Brozek, J., Helfand, M., Alonso-Coello, P., Glasziou, P., Jaeschke, R., Akl, E. A., Norris, S., Vist, G., Dahm, P., Shukla, V. K., Higgins, J., Falck-Ytter, Y., & Schünemann, H. J. (2011). GRADE guidelines: 7. Rating the quality of evidence – inconsistency. *Journal of Clinical Epidemiology, 64*, 1294-1302.

Guyatt, G. H., Oxman, A. D., Montori, V., Vist, G., Kunz, R., Brozek, J., Alonso-Coello, P., Djulbegovic, B., Atkins, D., Falck-Ytter, Y., Williams Jr, J. W., Meerpohl, J., Norris, S. L., Akl, E. A., & Schünemann, H. J. (2011). GRADE guidelines: 5. Rating the quality of evidence - publication bias. *Journal of Clinical Epidemiology, 64*, 1277-1282.

Guyatt, G. H., Oxman, A. D., Santesso, N., Helfand, M., Vist, G., Kunz, R., Brozek, J., Norris, S., Meerpohl, J., Djulbegovic, B., Alonso-Coello, P., Post, P. N., Busse, J. W., Glasziou, P., Christensen, R., & Schünemann, H. J. (2013). GRADE guidelines: 12. Preparing summary of findings tables - binary outcomes. *Journal of Clinical Epidemiology, 66*, 158-172.

Guyatt, G. H., Oxman, A. D., Sultan, S, Brozek, J., Glasziou, P., Alonso-Coello, P., Atkins, D., Kunz, R., Montori, V., Jaeschke, R., Rind, D., Dahm, P., Akl, E. A., Meerpohl, J., Vist, G., Berliner, E., Norris, S., Falck-Ytterr, Y., & Schünemann, H. J. (2013). GRADE guidelines: 11. Making an overall rating of confidence in effect estimates for a single outcome and for all outcomes. *Journal of Clinical Epidemiology, 66,* 151-157.

Guyatt, G. H., Oxman, A. D., Sultan, S., Glasziou, P., Akl, E. A., Alonso-Coello, P., Atkins, D., Kunz, R., Brozek, J., Montori, V., Jaeschke, R., Rind, D., Dahm, P., Meerpohl, J., Vist, G., Berliner, E., Norris, S., Falck-Ytter, Y., Murad, M. H., & Schünemann, H. J. (2011). GRADE guidelines: 9. Rating up the quality of evidence. *Journal of Clinical Epidemiology, 64*, 1311-1316.

Guyatt, G. H., Oxman, A. D., Vist, G., Kunz, R., Brozek, J., Alonso-Coello, P., Montori, V., Akl, E. A., Djulbegovic, B., Falck-Ytter, Y., Norris, S. L., Williams Jr., J. W., Atkins, D., Meerpohl, J., & Schünemann, H. J. (2011). GRADE guidelines: 4. Rating the quality of evidence - study limitations (risk of bias). *Journal of Clinical Epidemiology, 64*, 407-415.

Guyatt, G. H., Oxman, A. D., Vist, G. E., Kunz, R., Falck-Ytter, Y., Alonso-Coello, P., & Schünemann, H. J. (2008). Rating quality of evidence and strength of recommendations: GRADE: An emerging consensus on rating quality of evidence and strength of recommendations. *British Medical Journal, 336*(7650), 924-926.

Guyatt, G. H., & Rennie, D. (1993). Users' guides to the medical literature. *JAMA, 270*(17), 2096-2097.

Guyatt, G. H., Sackett, D. L., & Cook, D. J. (1994). Users' guides to the medical literature. II. How to use an article about therapy or prevention. B. What were the results and will they help me in caring for my patients? *JAMA, 271*(1), 59-63.

Guyatt, G. H., Sackett, D. L., Sinclair, J. C., Hayward, R., Cook, D. J., & Cook, R. J. (1995). Users' guides to the medical literature. IX. A method for grading health care recommendations.  *JAMA, 274*, 1800-1804.

Guyatt, G. H., Sinclair, J., Cook, D. J., & Glasziou, P. (1999). Users' guides to the medical literature: XVI. How to use a treatment recommendation. *JAMA, 281*(19), 1836-1843.

Guyatt, G. H., Thorlund, K., Oxman, A. D., Walter, S. D., Patrick, D., Furukawa, T. A., Johnston, B. C., Karanicolas, P., Akl, E. A., Vist, G., Kunz, R., Brozek, J., Kupper, L. L., Martin, S. L., Meerpohl, J. J., Alonso-Coello, P., Christensen, R., & Schünemann, H. J. (2013). GRADE guidelines: 13. Preparing Summary of Findings tables and evidence profiles - continuous outcomes. *Journal of Clinical Epidemiology, 66*, 173-183.

Haidet, P., Levine, R. E., Parmelee, D. X., Crow, S., Kennedy, F., Kelly, P. A., Perkowski, L., Michaelsen, L., & Richards, B. F. (2012). Perspective: Guidelines for reporting team-based learning activities in the medical and health sciences education literature. *Academic Medicine, 87*(3), 292-299.

Hans, E., & Hiller, W. (2013). A meta-analysis of nonrandomized effectiveness studies on outpatient cognitive behavioral therapy for adult anxiety disorders. *Clinical Psychology Review, 33*, 954–964.

Harbour, R., Lowe, G., & Twaddle, S. (2011). Scottish intercollegiate guidelines network: The first 15 years (1993–2008).  *The Journal of the Royal College of Physicians of Edinburgh, 41*, 163–168.

Harbour, R., & Miller, J. (2001). A new system for grading recommendations in evidence based guidelines. *BMJ, 323*(7308), 334-336.

Harrington, N. G., & Noar, S. M. (2012). Reporting standards for studies of tailored interventions. *Health Education Research, 27*(2), 331-342.

Harris, R. P., Helfand, M., Woolf, S. H., Lohr, K. N., Mulrow, C. D., Teutsch, S. M., . . . Atkins, D. (2001). Current methods of the U.S. Preventive Services Task Force. *American Journal of Preventive Medicine, 20*(3), 22-35.

Hayden, J. A., Coté, P., & Bombardier, C. (2006). evaluation of the quality of prognosis studies in systematic reviews. *Annals of Internal Medicine, 114*, 427-437.

Haynes, R. B., Wilczynski, N. L., & the Computerized Clinical Decision Support System (CCDSS) Systematic Review Team (2010). Effects of computerized clinical decision support systems on practitioner performance and patient outcomes: Methods of a decision-makerresearcher partnership systematic review. *Implementation Science, 5*. Retrieved from <http://www.implementationscience.com/content/5/1/12>

Hayward, R. S., Wilson, M. C., Tunis, S. R., Bass, E. B., & Guyatt, G. (1995). Users' guides to the medical literature. VIII. How to use clinical practice guidelines. A. Are the recommendations valid? *JAMA, 274*(7), 570-574.

Heidenreich, P. A., McDonald, K. M., Hastie, T., Fadel, B., Hagan, V., Lee, B. K., & Hlatky, M. A. (1999). *An evaluation of beta-blockers, calcium antagonists, nitrates, and alternative therapies for stable angina*(AHRQ Publication No. 00-E003). Rockville, MD: Agency for Healthcare Research and Quality.

Heinsman, D. T., & Shadish, W. R. (1996). Assignment methods in experimentation: When do nonrandomized experiments approximate answers from randomized experiments? *Psychological Methods, 1*(2), 154-169.

Heitz, C. A. M., Hilfiker, R., Bachmann, L. M., Joronen, H., Lorenz, T., Uebelhart, D., . . . Brunner, F. (2009). Comparison of risk factors predicting return to work between patients with subacute and chronic non-specific low back pain: Systematic review. *European Spine Journal, 18*(12), 1829-1835.

Helmhout, P. H., Staal, J. B., Maher, C. G., Petersen, T., Rainville, J., & Shaw, W. S. (2008). Exercise therapy and low back pain: Insights and proposals to improve the design, conduct, and reporting of clinical trials. *Spine, 33*(16), 1782-1788.

Hemminki, E. (1981). Quality of reports of clinical trials submitted by the drug industry to the Finnish and Swedish control authorities. *European Journal of Clinical Pharmacology, 19*, 157-165.

Higashida, R. T., Meyers, P. M., Phatouros, C. C., Connors, J. J., Barr, J. D., & Sacks, D. (2004). Reporting standards for carotid artery angioplasty and stent placement. *Stroke, 35*(5), 112-134.

Higgins, J. P. T., & Altman, D. G. (2008). Assessing risk of bias in included studies. In J. P. T. Higgins & S. Green (Eds.), *Cochrane handbook for systematic reviews of interventions, version 5.0.2*. Retrieved from http://www.cochrane-handbook.org.

Higgins, J. P. T., Altman, D. G., Gøtzsche, P. C., Jüni, P., Moher, D., Oxman, A. D., . . . Cochrane Statistical Methods Group (2011). The Cochrane Collaboration’s tool for assessing risk of bias in randomised trials. *BMJ, 343*. doi:10.1136/bmj.d5928

Higgins, J. P. T., & Thompson, S. G. (2004). Controlling the risk of spurious findings from meta-regression. *Statistics in Medicine, 23*, 1663–1682.

Hillberg, T., Hamilton-Giachritsis, C., & Dixon, L. (2011). Review of meta-analyses on the association between child sexual abuse and adult mental health difficulties: A systematic approach**.***Trauma Violence & Abuse, 12*(1), 38-49.

Hlatky, M. A., Greenland, P., Arnett, D. K., Ballantyne, C. M., Criqui, M. H., Elkind, M. S., . . . Wilson, W. F. (2009). Criteria for evaluation of novel markers of cardiovascular risk: A scientific statement from the American Heart Association. *Circulation, 119*(17), 2408-2416.

Hoffmann, T. C., Glasziou, P. P., Boutron, I., Milne, R., Perera, R., Moher, D., . . . Michie, S. (2014). Better reporting of interventions: Template for intervention description and replication (TIDieR) checklist and guide. *BMJ, 348*. doi:10.1136/bmj.g1687

Hollander, J. E., Blomkalns, A. L., Brogan, G. X., Diercks, D. B., Field, J. M., Garvey, J. L., . . . Jackson, R. E. (2004). Standardized reporting guidelines for studies evaluating risk stratification of ED patients with potential acute coronary syndromes. *Academic Emergency Medicine, 11*(12), 1331-1340.

Hollenbach, J. A., Mack, S. J., Gourraud, P. A., Single, R. M., Maiers, M., Middleton, D., . . . the Immunogenomics Data Analysis Working Group (2011). A community standard for immunogenomic data reporting and analysis: Proposal for a STrengthening the REporting of Immunogenomic Studies (STREIS) statement. *Tissue Antigens, 78*(5), 333-344.

Holt, T. A., Thorogood, M., & Griffiths, F. (2012). Changing clinical practice through patient specific reminders available at the time of the clinical encounter: Systematic review and meta-analysis. *Journal of General Internal Medicine, 27*(8), 974-984.

Holwerda, A., van der Klink, J. J. L., Groothoff, J. W., & Brouwer, S. (2012). predictors for work participation in individuals with an autism spectrum disorder: A systematic review. *Journal of Occupational Rehabilitation, 22*, 333–352.

Hooijmans, C. R., Leenaars, M., & Ritskes-Hoitinga, M. (2010). A gold standard publication checklist to improve the quality of animal studies, to fully integrate the Three Rs, and to make systematic reviews more feasible. *Alternatives to Laboratory Animals, 38*(2), 167-182.

Hopewell, S., Clarke, M., & Askie, L. (2006). Reporting of trials presented in conference abstracts needs to be improved. *Journal of Clinical Epidemiology, 59*(7), 681–684.

Hopewell, S., Clarke, M., Moher, D., Wager, E., Middleton, P., Altman, D. G., . . . the CONSORT Group. (2008). CONSORT for reporting randomised trials in journal and conference abstracts. *The Lancet, 371*(9609), 281-283.

Hopley, C., Stengel, D., Ekkernkamp, A., & Wich, M. (2010). Primary total hip arthroplasty versus hemiarthroplasty for displaced intracapsular hip fractures in older patients:
 Systematic review. *BMJ, 340*. doi:10.1136/bmj.c2332.

Howick, J., Chalmers, I., Glasziou, P., Greenhalgh, T., Heneghan, C., Liberati, A., . . . Hodgkinson, M. (2011). *The Oxford 2011 levels of evidence*. Retrieved from http://www.cebm.net/index.aspx?o=5653

Huebner, R. H., Park, K. C., Shepherd, J. E., Schwimmer, J., Czernin, J., Phelps, M. E., & Gambhir, S. S. (2000). A meta-analysis of the literature for whole-body FDG PET detection of recurrent colorectal cancer. *The Journal of Nuclear Medicine, 41*, 1177–1189.

Hundley, W. G., Bluemke, D., Bogaert, J. G., Friedrich, M. G., Higgins, C. B., Lawson, M. A., . . . Neubauer, S. (2009). Society for Cardiovascular Magnetic Resonance guidelines for reporting cardiovascular magnetic resonance examinations. *Journal of Cardiovascular Magnetic Resonance, 11*. Retrieved from <http://www.jcmr-online.com/content/11/1/5>

Hunt, D. L., Jaeschke, R., & McKibbon, K. A. (2000). Users' guides to the medical literature: XXI. Using electronic health information resources in evidence-based practice. *JAMA, 283*(14), 1875-1879.

Husereau, D., Drummond, M., Petrou, S., Carswell, C., Moher, D., Greenberg, D., . . . Loder, E. (2013). Consolidated Health Economic Evaluation Reporting Standards (CHEERS) statement. *European Journal of Health Economics, 14*(3), 367-372.

Hyde, M. L. (2000). Reasonable psychometric standards for self-report outcome measures in audiological rehabilitation. *Ear and Hearing, 21*(Suppl. 4), 24-36.

Idris, A. H., Becker, L. B., Ornato, J. P., Hedges, J. R., Bircher, N. G., Chandra, N. C., . . . Weil, M. H. (1996). Utstein-style guidelines for uniform reporting of laboratory CPR research. A statement for healthcare professionals from a task force of the American Heart Association, the American College of Emergency Physicians, the American College of Cardiology, the European Resuscitation Council, the Heart and Stroke Foundation of Canada, the Institute of Critical Care Medicine, the Safar Center for Resuscitation Research, and the Society for Academic Emergency Medicine. *Circulation, 94*(9), 2324-2336.

Ioannidis, J. P. A., Evans, S. J. W., Gøtzsche, P. C., O’Neill, R. T., Altman, D. G., Schulz, K., & Moher, D. (2004). Better reporting of harms in randomized trials: An extension of the CONSORT statement. *Annals of Internal Medicine, 141*, 781-788.

Jabs, D. A. (2005). Improving the reporting of clinical case series. *American Journal of Ophthalmology, 139*(5), 900-905.

Jackson, D. L. (2010). Reporting results of latent growth modeling and multilevel modeling analyses: Some recommendations for rehabilitation psychology. *Rehabilitation Psychology, 55*(3), 272-285.

Jadad, A. R., Moore, R. A., Carroll, D., Jenkinson, C., Reynolds, D. J. M., Gavaghan, D. J., & McQuay, H. J. (1996). Assessing the quality of reports of randomized clinical trials: Is blinding necessary? *Controlled Clinical Trials, 17*(1), 1-12.

Jaeschke, R., Guyatt, G. H., & Sackett, D. L. (1994). Users' guides to the medical literature. III. How to use an article about a diagnostic test. A. Are the results of the study valid? *JAMA, 271*(5), 389-391.

Janssens, A. C., Ioannidis, J. P., van Duijn, C. M., Little, J., Khoury, M. J., & Group, G. (2011). Strengthening the reporting of Genetic RIsk Prediction Studies: the GRIPS statement. *PLoS Medicine, 8*(3). doi:10.1371/journal.pmed.1000420

Jarde, A., Losilla, J.-M., Vives, J., & Rodrigo, M. F. (2013). Q-Coh: A tool to screen the methodological quality of cohort studies in systematic reviews and meta-analyses. *International Journal of Clinical and Health Psychology, 13*(2), 138-146.

Jefferson, T., Di Pietrantonj, C., Debalini, M. G., Rivetti, A., & Demicheli, V. (2009). Relation of study quality, concordance, take home message, funding, and impact in studies of influenza vaccines: Systematic review. *BMJ, 338*. doi:10.1136/bmj.b354

Jiménez-Requena, F., Delgado-Bolton, R. C., Fernández-Pérez, C., Gambhir, S. S., Schwimmer, J., Pérez-Vázquez, J. M., & Carreras-Delgado, J. L. (2009). Meta-analysis of the performance of F-FDG PET in cutaneous melanoma. *European Journal of Nuclear Medicine and Molecular Imaging, 37*(2), 284-300.

Johnson, B. T., MacDonald, H. V., Bruneau Jr., M. L., Goldsby, T. U., Brown, J. C., Huedo–Medina, T. B., & Pescatello, L. S. (2014). Methodological quality of meta-analyses on the blood pressure response to exercise: A review. *Journal of Hypertension, 32*, 706–723.

Jorgensen, A. L., & Williamson, P. R. (2008). Methodological quality of pharmacogenetic studies: Issues of concern. *Statistics in Medicine, 27*, 6547–6569.

Joubert, B. R., Reif, D. M., Edwards, S. W., Leiner, K. E., Hudgens, E. E., Egeghy, P., . . . Hubal, E. C. (2011). Evaluation of genetic susceptibility to childhood allergy and asthma in an African American urban population. *BMC Medical Genetics, 12*. doi:10.1186/1471-2350-12-25

Jüni, P., Altman, D. G., & Egger, M. (2001). Assessing the quality of randomised controlled trials. In M. Egger, G. D. Smith, & D. G. Altman (Eds.), *Systematic reviews in health care* (pp. 87-108). London, UK: BMJ.

Jüni, P., Witschi, A., Bloch, R., & Egger, M. (1999). The hazards of scoring the quality of clinical trials for meta-analysis. *JAMA, 282*(11), 1054-1060.

Karmy-Jones, R., Ferrigno, L., Teso, D., Long, W. B., & Shackford, S. (2011). Endovascular repair compared with operative repair of traumatic rupture of the thoracic aorta: A nonsystematic review and a plea for trauma-specific reporting guidelines. *The Journal of Trauma, 71*(4), 1059-1072.

Kausch, I., Sommerauer, M., Montorsi, F., Stenzl, A., Jacqmin, D., Jichlinski, P., . . . Vonthein, R. (2010). Photodynamic diagnosis in non–muscle-invasive bladder cancer: A systematic review and cumulative analysis of prospective studies. *European Urology, 57*, 595–606.

Kawai, K., Spiegelman, D., Shankarb, A. H., & Fawzi, W. W. (2011). Maternal multiple micronutrient supplementation and pregnancy outcomes in developing countries: Meta-analysis and meta-regression. *Bulletin of the World Health Organization, 89*, 402–411.

Kearon, C., Iorio, A., & Palareti, G. (2010). Risk of recurrent venous thromboembolism after stopping treatment in cohort studies: recommendation for acceptable rates and standardized reporting. *Journal of Thrombosis and Haemostasis, 8*(10), 2313-2315.

Kelley, K., Clark, B., Brown, V., & Sitzia, J. (2003). Good practice in the conduct and reporting of survey research. *International Journal for Quality in Health Care, 15*(3), 261-266.

Kelly, W. N., Arellano, F. M., Barnes, J., Bergman, U., Edwards, R. I., Fernandez, A. M., . . . Wise, R. P. (2007). Guidelines for submitting adverse event reports for publication. *Pharmacoepidemiology and Drug Safety, 16*(5), 581-587.

Kempen, J. H. (2011). Appropriate use and reporting of uncontrolled case series in the medical literature. *American Journal of Ophthalmology, 151*(1), 7-10.

Kennedy, C. A., Amick, B. C., Dennerlein, J. T., Brewer, S., Catli, S., Williams, R., . . . Rempel, D. (2010). Systematic review of the role of occupational health and safety interventions in the prevention of upper extremity musculoskeletal symptoms, signs, disorders, injuries, claims and lost time. *Journal of Occupational Rehabilitation, 20*, 127–162.

Kennedy, C. E., Medley, A. M., Sweat, M. D., & O’Reilly, K. R. (2010). Behavioural interventions for HIV positive prevention in developing countries: A systematic review and meta-analysis. *Bulletin of the World Health Organization, 88*, 615–623.

Kent, D. L., Haynor, D. R., Larson, E. B., & Deyo, R. A. (1992). Diagnosis of lumbar spinal stenosis in adults: a metaanalysis of the accuracy of CT, MR, and myelography. *American Journal of Roentgenology, 158*, 1135-1144.

Khan, K. S., Ter Riet, G., Glanville, J., Sowden, A. J., & Kleijnen, J. (2000). *Undertaking systematic reviews of research on effectiveness: CRD's guidance for those carrying out or commissioning reviews*. Retrieved from http://www.york.ac.uk/inst/crd/pdf/Systematic_Reviews.pdf.

Kienle, G. S., Hamre, H. J., Portalupi, E., & Kiene, H. (2004). Improving the quality of therapeutic reports of single cases and case series in oncology – criteria and checklist. *Alternative Therapies in Health and Medicine, 10*, 68–72.

Kilkenny, C., Browne, W. J., Cuthill, I. C., Emerson, M., & Altman, D. G. (2010). Improving bioscience research reporting: The ARRIVE guidelines for reporting animal research. *PLoS Biolology 8*(6). doi:10.1371/journal.pbio.1000412

Kleijnen, J., Knipschild, P., & ter Riet, G. (1991). Clinical trials of homoeopathy. *BMJ, 302*, 316-323.

Kmet, L., Lee, R., & Cook, L. (2004). *Standard quality assessment criteria for evaluating primary research papers from a variety of fields*. Edmonton, Canada: Alberta Heritage Foundation for Medical Research.

Koch, S., Kunz, T., Lykou, S., & Cruz, R. (2014). Effects of dance movement therapy and dance on health-related psychological outcomes: A meta-analysis. *The Arts in Psychotherapy, 41*(1), 46-64.

Kohrt, B. A., Rasmussen, A., Kaiser, B. N., Haroz, E. E., Maharjan, S. M., Mutamba, B. B., . . . Hinton, D. E. (2015). Cultural concepts of distress and psychiatric disorders: Literature review and research recommendations for global mental health epidemiology. *International Journal of Epidemiology, 43*, 365–406.

Koricheva, J., & Gurevitch, J. (2014). Uses and misuses of meta-analysis in plant ecology. *Journal of Ecology, 102*, 828–844.

Kottner, J., Audigéb, L., Brorsonc, S., Donnerd, A., Gajewskie, B. J., Hróbjartssonf, A., . . . Streiner, D. L. (2011). Guidelines for Reporting Reliability and Agreement Studies (GRRAS) were proposed. *Journal of Clinical Epidemiology, 64*, 96-106.

Kung, J., Chiappelli, F., Cajulis, O. O., Avezova, R., Kossan, G., Chew, L., & Maida, C. A. (2010). From systematic reviews to clinical recommendations for evidence-based health care: Validation of Revised Assessment of Multiple Systematic Reviews (R-AMSTAR) for Grading of Clinical Relevance. *The Open Dentistry Journal, 4*, 84-91.

Kunz, R., & Oxman, A. D. (1998). The unpredictability paradox: Review of empirical comparisons of randomised and non-randomised clinical trials. *British Medical Journal, 317*(7167), 1185-1190.

Lagerveld, S. E., Bültmann, U., Franche, R. L., van Dijk, F. J. H., Vlasveld, M. C., van der Feltz-Cornelis, C. M., . . . Nieuwenhuijsen, K. (2010). Factors associated with work participation and work functioning in depressed workers: A systematic review. *Journal of Occupational Rehabilitation, 20*, 275–292.

Langhelle, A., Nolan, J., Herlitz, J., Castren, M., Wenzel, V., Soreide, E., . . . 2003 Utstein Consensus Symposium (2005). Recommended guidelines for reviewing, reporting, and conducting research on post-resuscitation care: The Utstein style. *Resuscitation, 66*(3), 271-283.

Lapane, K. L., Yang, S., Brown, M. J., Jawahar, R., Pagliasotti, C., & Rajpathak, S. (2013). Sulfonylureas and risk of falls and fractures: A systematic review. *Drugs Aging, 30*, 527–547.

Lassmann, M., Chiesa, C., Flux, G., & Bardiès, M. (2011). EANM Dosimetry Committee guidance document: Good practice of clinical dosimetry reporting. *European Journal of Nuclear Medicine and Molecular Imaging, 38*(1), 192-200.

Lau, J., Ioannidis, J. P., Balk, E., Milch, C., Chew, P., Terrin, N., . . . Wong, J. B. (2000). *Evaluation of technologies for identifying acute cardiac ischemia in emergency departments: Summary*. Rockville, MD: Agency for Healthcare Research and Quality. Retrieved from http://www.ahrq.gov/clinic/cardsum.htm

Laupacis, A., Sekar, N., & Steill, I. G. (1997). Clinical prediction rules: A review and suggested modifications of methodological standards. *JAMA, 277*, 488–494.

Laupacis, A., Wells, G., Richardson, W. S., & Tugwell, P. (1994). Users' guides to the medical literature. V. How to use an article about prognosis. *JAMA, 272*, 234-237.

Law, M., Stewart, D., Letts, L., Pollock, N., Bosch, J., & Westmoreland, M. (1998). *Critical review form–quantitative studies*. Retrieved from http:// www.srs-mcmaster.ca/Portals/20/pdf/ebp/quanreview_form1.doc

Lee, C. W., & Chi, K. N. (2000). The standard of reporting of health-related quality of life in clinical cancer trials. *Journal of Clinical Epidemiology, 53*(5), 451-458.

Leech, N. L., & Onwuegbuzie, A. J. (2010). Guidelines for conducting and reporting mixed research in the field of counseling and beyond. *Journal of Counseling and Development, 88*(1), 61-69.

Leonardi, M. (2006). Public health support to policies in the fields of headache. Different ways of producing data and modalities of reading them with the aid of the meta-analytic approach. *The Journal of Headache and Pain, 7*(3), 157-159.

Levine, M., Walter, S., Lee, H., Haines, T., Holbrook, A., & Moyer, V. (1994). Users' guides to the medical literature. IV. How to use an article about harm. *JAMA, 271*, 1615-1619.

Li, L. C., Moja, L., Romero, A., Sayre, E. C., & Grimshaw, J. M. (2009). Nonrandomized quality improvement intervention trials might overstate the strength of causal inference of their findings. *Journal of Clinical Epidemiology, 62*(9), 959-966.

Li, T., Puhan, M. A., Vedula, S. S., Singh, S., & Dickersin, K. (2011). Network meta-analysis-highly attractive but more methodological research is needed. *BMC Medicine, 9*. Retrieved from <http://www.biomedcentral.com/1741-7015/9/79>

Liberati, A., Altman, D. G., Tetzlaff, J., Mulrow, C., Gøtzsche, P. C., Ioannidis, J. P. A., . . . Moher, D. (2009). The PRISMA statement for reporting systematic reviews and meta-analyses of studies that evaluate healthcare interventions: explanation and elaboration. *BMJ, 339*. doi:10.1136/bmj.b2700

Lijmer, J. G., Mol, B. W., Heisterkamp, S., Bonsel, G. J., Prins, M. H., van der Meulen, J. H., & Bossuyt, P. M. (1999). Empirical evidence of design-related bias in studies of diagnostic tests. *JAMA, 282*, 1061-1066.

Linde, K. (2009). Can you trust systematic reviews of complementary and alternative therapies? *European Journal of Integrative Medicine, 1*(3), 117-123.

Linde, K., Niemann, K., Schneider, A., & Meissner, K. (2010). How large are the nonspecific effects of acupuncture? A meta-analysis of randomized controlled trials. *BMC Medicine, 8*, 75. doi:[10.1186/1741-7015-8-75](http://dx.doi.org/10.1186%2F1741-7015-8-75)

Linde, K., Ramirez, G., Mulrow, C. D., Pauls, A., Weidenhammer, W., & Melchart, D. (1996). St John’s wort for depression—an overview and metaanalysis of randomised clinical trials. *BMJ, 313*, 253–258.

Lipsey, M., & Wilson, D. (2001). *Practical meta-analysis*. Thousand Oaks, CA: Sage publications.

List, T., & Axelsson, S. (2010). Management of TMD: Evidence from systematic reviews and meta-analyses. *Journal of Oral Rehabilitation, 37*, 430–451.

Little, J. (2006). *HuGE review handbook, version 1.0*. Ottawa, Ontario, Canada: HuGENet Canada Coordinating Centre. Retrieved from http://www.med.uottawa.ca/public-health-genomics/web/assets/documents/HuGE_Review_Handbook_V1_0.pdf.

Little, J., Higgins, J. P., Ioannidis, J. P., Moher, D., Gagnon, F., von Elm, E., . . . Birkett, N. (2009). STrengthening the REporting of Genetic Association studies (STREGA): An extension of the STROBE Statement. *Annals of Internal Medicine, 150*, 206–215.

Lu, Y. Y., Chen, J. H., Liang, J. A., Wang, H. Y., Lin, C. C., Lin, W. Y., & Kao, C. H. (2012). Clinical value of FDG PET or PET/CT in urinary bladder cancer: A systemic review and meta-analysis. *European Journal of Radiology, 81*(9), 2411-2416.

Lubans, D. R., Foster, C., & Biddle, S. J. H. (2008). A review of mediators of behavior in interventions to promote physical activity among children and adolescents. *Preventive Medicine, 47*, 463-470.

MacDermid, J. C. (2004). An introduction to evidence-based practice for hand therapists. *Journal of Hand Therapy, 17*(2), 105-117.

Macdonald, L. E., Brett, J., Kelton, D., Majowicz, S. E., Snedeker, K., & Sargeant, J. M. (2011). A systematic review and meta-analysis of the effects of pasteurization on milk vitamins, and evidence for raw milk consumption and other health-related outcomes. *Journal of Food Protection, 74*(11), 1814–1832.

Macleod, M. R., Fisher, M., O'Collins, V., Sena, E. S., Dirnagl, U., Bath, P. M., . . . Howells, D. W. (2009). Good laboratory practice: Preventing introduction of bias at the bench. *Stroke, 40*(3), 50-52.

MacPherson, H., Altman, D. G., Hammerschlag, R., Youping, L., Taixiang, W., White, A., & Moher, D. (2010). Revised STandards for Reporting Interventions in Clinical Trials of Acupuncture (STRICTA): Extending the CONSORT statement. *PLoS Medicine, 7*(6). doi:10.1371/journal.pmed.1000261

MacPherson, H., White, A., Cummings, M., Jobst, K., Rose, K., & Niemtzow, R. (2002). Standards for reporting interventions in controlled trials of acupuncture: The STRICTA recommendations. *Acupuncture in Medicine, 20*(1), 22-25.

Maharaj, R., & Metaxa, V. (2011). Levosimendan and mortality after coronary revascularisation: A meta-analysis of randomised controlled trials. *Critical Care, 15*(3). doi:10.1186/cc10263

Malterud, K. (2001). Qualitative research: Standards, challenges, and guidelines. *The Lancet, 358*(9280), 483-488.

Manterola, C., Vial, M., Pineda, V., & Sanhueza, A. (2009). Revisión sistemática de la literatura con diferentes tipos de diseños. *International Journal of Morphology, 27*, 1179-1186.

Marchevsky, A. M., & Gupta, R. (2011). Development of evidence-based diagnostic criteria and prognostic/predictive models: Experience at Cedars-Sinai Medical center. In A. M. Marchevsky, & M. Wick (Eds.), *Evidence based pathology and laboratory medicine* (pp. 245-260). Los Angeles, CA: Springer.

Matthiessen, P. F., & Bornhöft, G. (2011). General problems with clinical trials in research. In G. Bornhöft, & P. F. Matthiessen (Eds.), *Homeopathy in healthcare. Effectiveness, appropriateness, safety, costs* (pp. 27-46). Verlag-Berlin-Heidelberg: Springer.

Maxwell, L., Santesso, N., Tugwell, P. S., Wells, G. A., Judd, M., & Buchbinder, R. (2006). Method guidelines for Cochrane Musculoskeletal Group systematic reviews. *Journal of Rheumatology, 33*, 2304-2311.

Mayer, E., Darzi, L. A., & Athanasiou, T. (2010). The role of volume–outcome relationship in surgery. In T. Athanasiou (Ed.), *Key topics in surgical research and methodology*(pp. 195-206). Verlag-Berlin-Heidelberg: Springer.

McAlister, F. A., Clark, H. D., van Walraven, C., Straus, S. E., Lawson, F. M. E., Moher, D., & Mulrow, C. D. (1999). The medical review article revisited: Has the science improved? *Annals of Internal Medicine, 131*(12), 947-951.

McAlister, F. A., Laupacis, A., Wells, G. A., & Sackett, D. L. (2007). Users’ guides to the medical literatura XIX. Applying clinical trial results B. Guidelines for determining whether a drug is exerting (more than) a class effect. *JAMA, 282*(14), 1371-1377.

McAlister, F. A., Straus, S. E., Guyatt, G. H., & Haynes, R. B. (2000). Users' guides to the medical literature: XX. Integrating research evidence with the care of the individual patient. *JAMA, 283*(21), 2829-2836.

McCrory, D. C., Matchar, D. B., Bastian, L., Datta, S., Hasselblad, V., Hickey, J., . . . Nanda, K. (1999). *Evaluation of cervical cytology* (AHCPR Publication No.99-E010). Rockville, MD: Agency for Health Care Policy and Research.

McGilloway, A., Hall, R. E., Lee, T., & Bhui, K. S. (2010). A systematic review of personality disorder, race and ethnicity: Prevalence, aetiology and treatment. *BMC Psychiatry, 10*, 33. Retrieved from <http://www.biomedcentral.com/1471-244X/10/33>

McGinn, T. G., Guyatt, G. H., Wyer, P. C., Naylor, C. D., & Richardson, W. S. (2000). Users’ guides to the medical literature: XXII. How to use articles about clinical decision rules. *JAMA, 284*(1), 79-84.

McGrath, D., L. (2009). Cannabis: A clue or a distraction in the search for ‘causes’ of psychosis? In W.F. Gattaz, & G. Busatto (Eds.), *Advances in schizophrenia research* (pp. 367-376). New York-Dordrecht-Heidelberg-London: Springer.

McGuire, J., Bates, G. W., Dretzke, B. J., McGivern, J. E., Rembold, K. L., Seabold, D. R., . . . Levin, J. R. (1985). Methodological quality as a component of meta-analysis. *Educational Psychologist, 20*(1), 1-5.

McNeely, M. L., Campbell, K. L., Rowe, B. H., Klassen, T. P., Mackey, J. R., & Courneya, K. S. (2006). Effects of exercise on breast cancer patients and survivors: A systematic review and meta-analysis. *Canadian Medical Association Journal, 175*, 34–41.

McShane, L. M., Altman, D. G., Sauerbrei, W., Taube, S. E., Gion, M., & Clark, G. M. (2005). REporting recommendations for tumour MARKer prognostic studies (REMARK). *British Journal of Cancer, 93*(4), 387-391.

Meads, C. A., & Davenport, C. F. (2009). Quality assessment of diagnostic before-after studies: Development of methodology in the context of a systematic review. *BMC Medical Research Methodology, 9*, 3. Retrieved from <http://www.biomedcentral.com/1471-2288/9/3>

Melby, M. K., Sievert, L. L., Anderson, D., & Obermeyer, C. M. (2011). Overview of methods used in cross-cultural comparisons of menopausal symptoms and their determinants: Guidelines for Strengthening the Reporting of Menopause and Aging (STROMA) studies. *Maturitas, 70*(2), 99-109.

Mello, L. F. D., Nóbrega, L. F., & Lemos, A. (2011). Transcutaneous electrical stimulation for pain relief during labor: A systematic review and meta-analysis. *Revista Brasileira de Fisioterapia, 15*(3), 175-184.

Metcalf, B., Henley, W., & Wilkin, T. (2012). Effectiveness of intervention on physical activity of children: Systematic review and meta-analysis of controlled trials with objectively measured outcomes. *BMJ, 345*. doi:10.1136/bmj.e5888

Meyer, E., Delaney, M., Lin, Y., Morris, A., Pavenski, K., Tinmouth, A., . . . Dumont, L. J. (2012). A reporting guideline for clinical platelet transfusion studies from the BEST Collaborative. *Transfusion, 53*, 1328-1334.

Mijnhout, G. S., Alkhalaf, A., Kleefstra, N., & Bibo, H. J. G. (2010). Alpha lipoic acid: A new treatment for neuropathic pain in patients with diabetes? *Netherlands Journal of Medicine, 68*(4), 158-162.

Minelli, C., Thompson, J. R., Abrams, K. R., Thakkinstian, A., & Attia, J. (2007). How should we use information about HWE in the meta-analyses of genetic association studies? *International Journal of Epidemiology, 37*(1), 136–146.

Mirza, I., & Jenkins, R. (2004). Risk factors, prevalence, and treatment of anxiety and depressive disorders in Pakistan: Systematic review. *BMJ, 328*, 1-5.

Mistiaen, P., & van Halm-Walters, M. (2010). Prevention and treatment of intertrigo in large skin folds of adults: A systematic review. *BMC Nursing, 9*. Retrieved from <http://www.biomedcentral.com/1472-6955/9/12>

Moberg-Mogren, E., & Nelson, D. (2006). Evaluating the quality of reporting occupational therapy randomized controlled trials by expanding the CONSORT criteria. *American Journal of Occupational Therapy, 60*(2), 226-235.

Moher, D., Cook, D., Eastwood, S., Olkin, I., Rennie, D., & Stroup, D. (1999). Improving the quality of reports of meta-analyses of randomised controlled trials: the QUOROM statement. *Lancet, 354*(9193), 1896-1900.

Moher, D., Fortin, P., Jadad, A. R., Jüni, P., Klassen, T., Le Lorier, J., . . . Penna, A. (1996). Completeness of reporting of trials published in languages other than English: Implications for conduct and reporting of systematic reviews. *Lancet, 347*, 363-366.

Moher, D., Jadad, A. R., Nichol, G., Penman, M., Tugwell, P., & Walsh, S. (1995). Assessing the quality of randomized controlled trials: An annotated bibliography of scales and checklists. *Controlled Clinical Trials, 16*(1), 62-73.

Moher, D., Jadad, A. R., & Tugwell, P. (1996). Assessing the quality of randomized controlled trials: Current issues and future directions. *International Journal of Technology Assessment in Health Care, 12*(2), 195-208.

Moher, D., Liberati, A., Tetzlaff, J., & Altman, D. G. (2009). Preferred reporting items for systematic review and meta-analyses: the PRISMA statement. *BMJ, 339*, 332-336.

Moher, D., Pham, B., Jones, A., Cook, D. J., Jadad, A. R., Moher, M., . . . Klassen, T. P. (1998). Does quality of reports of randomised trials affect estimates of intervention efficacy reported in meta-analyses? *The Lancet, 352*, 609-613.

Moher, D., Schulz, K. F., & Altman, D. G. (2001). The CONSORT statement: Revised recommendations for improving the quality of reports of parallel-group randomised trials. *The Lancet, 357*, 1191-1194.

Möhler, R., Bartoszek, G., Köpke, S., & Meyer, G. (2012). Proposed criteria for reporting the development and evaluation of complex interventions in healthcare (CReDECI): Guideline development. *International Journal of* *Nursing Studies, 49*(1), 40-6.

Moja, L. P., Telaro, E., D’Amico, R., Moschetti, I., Coe, L., & Liberati, A. (2005). Assessment of methodological quality of primary studies by systematic reviews: Results of the metaquality cross sectional study. *BMJ, 330*, 1053-1058.

Mokkink, L. B., Terwee, C. B., Stratford, P. W., Alonso, J., Patrick, D. L., Riphagen, I., . . . de Vet, H. C. W. (2009). Evaluation of the methodological quality of systematic reviews of health status measurement instruments. *Quality of Life Research, 18*(3), 313–333.

## Möller, H. J., & Maier, W. (2010). Evidence-based medicine in psychopharmacotherapy: Possibilities, problems and limitations. *European Archives of Psychiatry and Clinic Neuroscience, 260*(1), 25-39. doi:10.1007/s00406-009-0070-9

Moncrieff, J., Churchill, R., Drummond, D. C., & Mcguire, H. (2001). Development of a quality assessment instrument for trials of treatments for depression and neurosis. *International Journal of Methods in Psychiatric Research, 10*(3), *126-133*.

Moore, E. R., Anderson, G. C., & Bergman, N. (2007). Early skin-to-skin contact for mothers and their healthy newborn infants. *Cochrane Database of Systematic Reviews, 3*.

Moore, H. M., Kelly, A. B., Jewell, S. D., McShane, L. M., Clark, D. P., Greenspan, R., . . . Vaught, J. (2011). Biospecimen reporting for improved study quality (BRISQ). *Cancer Cytopathology, 119*(2), 92-101.

Moore, R. A., Derry, S., Wiffen, P. J., Straube, S., & Bendtsen, L. (2014). Evidence for efficacy of acute treatment of episodic tension-type headache: Methodological critique of randomised trials for oral treatments. *Pain, 155*(11), 2220-2228.

Moss, F., & Thompson, R. (1999). A new structure for quality improvement reports. *Quality in Health Care, 8*(2), 76.

Muche-Borowski, C., Kopp, M., Reese, I., Sitter, H., Werfel, T., & Schafer, T. (2010). Allergy prevention. *Journal der Deutschen Dermatologischen Gesellschaft, 8*(9), 718-724.

Müller-Riemenschneider, F., Roll, S., Friedrich, M., Zieren, J., Reinhold, T., Von Der Schulenburg, J. M. G., . . . Willich, S. N. (2007). Medical effectiveness and safety of conventional compared to laparoscopic incisional hernia repair: A systematic review. *Surgical Endoscopy, 21*(12), 2127-2136.

Muller-Stich, B. P., Senft, J. D., Warschkow, R., Kenngott, H. G., Billeter, A. T., Vit, G., . . . Nawroth, P. (2015). Surgical versus medical treatment of type 2 diabetes mellitus in nonseverely obese patients: A systematic review and meta-analysis. *Annals of Surgery, 261*(3), 421-429.

Munday, J., Hines, S., Wallace, K., Chang, A. M., Gibbons, K., & Yates, P. (2014). A systematic review of the effectiveness of warming interventions for women undergoing cesarean section. *Worldviews on Evidence-Based Nursing, 11*(6), 383-393.

National Health and Medical Research Council (2000a). *How to review the evidence: Systematic identification and review of the scientific literature.* Canberra, Australia: National Health and Medical Research Council.

National Health and Medical Research Council (2000b). *How to use the evidence: Assessment and application of scientific evidence.* Canberra, Australia: National Health and Medical Research Council.

National Institute for Health and Clinical Excellence (2012). *The guidelines manual*. London, UK: National Institute for Health and Clinical Excellence.

Naylor, C. D., & Guyatt, G. H. (1996a). Users' guides to the medical literature. X. How to use an article reporting variations in the outcomes of health services. *JAMA, 275*(7), 554-558.

Naylor, C. D., & Guyatt, G. H. (1996b). Users' guides to the medical literature. XI. How to use an article about a clinical utilization review. *JAMA, 275*(18), 1435-1439.

Nedeltchev, K., Pattynama, P. M., Biaminoo, G., Diehm, N., Jaff, M. R., Hopkins, L. N., . . . Group, D. (2010). Standardized definitions and clinical endpoints in carotid artery and supra-aortic trunk revascularization trials. *Catheterization and Cardiovascular Interventions, 76*(3), 333-344.

Nellensteijn, J., Ostelo, R., Bartels, R., Peul, W., van Royen, B., & van Tulder, M. (2009). Transforaminal endoscopic surgery for symptomatic lumbar disc herniations: A systematic review of the literature. *European Spine Journal, 19*(2), 181-204.

Newhouse, R. P., Stanik-Hutt, J., White, K. M., Johantgen, M., B., B. E., Zangaro, G., . . . Weiner, J. P. (2011). Advanced practice nurse outcomes 1990-2008: A systematic review. *Nursing Economics, 29*(5), 230-250.

Newman, M., & Elbourne, D. R. (2004). Guidelines for the REPOrting of primary empirical research Studies in Education (The REPOSE guidelines). *Evaluation & Research in Education, 18*(4), 201-212.

Newton, P. N., Lee, S. J., Goodman, C., Fernandez, F. M., Yeung, S., Phanouvong, S., . . . White, N. J. (2009). Guidelines for field surveys of the quality of medicines: A proposal. *PLoS Medicine, 6*(3). doi:10.1371/journal.pmed.1000052

Nicholson, A., Berger, K., Bohn, R., Carcao, M., Fischer, K., Gringeri, A., . . . Feldman, B. M. (2008). Recommendations for reporting economic evaluations of haemophilia prophylaxis: A nominal groups consensus statement on behalf of the Economics Expert Working Group of the International Prophylaxis Study Group. *Haemophilia, 14*(1), 127-132.

O'Brien, B. J., Heyland, D., Richardson, W. S., Levine, M., & Drummond, M. F. (1997). Users' guides to the medical literature: XIII. How to use an article on economic analysis of clinical practice. B. What are the results and will they help me in caring for my patients? *JAMA, 277*(22), 1802-1806.

O'Cathain, A., Murphy, E., & Nicholl, J. (2008). The quality of mixed methods studies in health services research. *Journal of Health Services Research & Policy, 13*(2), 92-98.

O'Connor, A. M., Sargeant, J. M., Gardner, I. A., Dickson, J. S., Torrence, M. E., Participants, C. M., . . . Wills, R. (2010). The REFLECT statement: Methods and processes of creating reporting guidelines for randomized controlled trials for livestock and food safety by modifying the CONSORT statement. *Zoonoses and Public Health, 57*(2), 95-104.

O’Rourke, K., & Detsky, A. S. (1989). Meta-analysis in medical research: Strong encouragement for higher quality in individual research efforts. *Journal of Clinical Epidemiology, 42*(10), 1021-1024.

Olivares, J., Rosa, A. I., & Sánchez-Meca, J. (2000). Meta-análisis de la eficacia de las habilidades de afrontamiento en problemas clínicos y de salud en España [Meta-analysis of the effectiveness of coping skills with respect to clinical and health problems in Spain]. *Anuario de Psicología, 31*(1), 43-61.

Oliver, S., Nicholas, A., & Oakley, A. (1996). Promoting health after sifting the evidence (PHASE): 12 questions to help you make sense of a process evaluation. Retrieved from http://eppi.ioe.ac.uk/eppi/Evidence/EPPI_reviews/Health_promotion/Review3/promo_health_after_sift_evid.pdf.

Olkin, I. (1995). Statistical and theoretical considerations in meta-analysis. [*Journal of Clinical Epidemiology*](http://www.sciencedirect.com/science/journal/08954356)*, 48*(1), 133-146.

Olson, S. H., Voigt, L. F., Begg, C. B., & Weiss, N. S. (2002). Reporting participation in case-control studies. *Epidemiology, 13*(2), 123-126.

Ottawa Hospital Research Institute (2013). *Suggested risk of bias criteria for EPOC reviews*. . Ottawa, Canada: OHRI. Retrieved from http://epoc.cochrane.org/search/google-appliance/Suggested%20risk%20of%20bias%20criteria

Oxford Centre for Evidence-Based Medicine (2009). *Levels of evidence*. Retrieved from http://www.cebm.net/index.aspx?o=1025

Oxman, A. D., Cook, D. J., & Guyatt, G. H. (1994). Users’ guides to the medical literature. VI. How to use an overview. *The Journal of the American Medical Association, 272*, 1367-1371.

Oxman, A. D., & Guyatt, G. H. (1991). Validation of an index of the quality of review articles. *Journal of Clinical Epidemiology, 44*, 1271-1278.

Oxman, A. D., Guyatt, G. H., Singer, J., Goldsmith, C. H., Hutchison, B. G., Milner, R. A., & Streiner, D. L. (1991). Agreement among reviewers of review articles. *Journal of Clinical Epidemiology, 44*, 91-98.

Oxman, A. D., Sackett, D. L., & Guyatt, G. H. (1993). Users' guides to the medical literature. I. How to get started. *JAMA, 270*(17), 2093-2095.

Palermo, T. M., Eccleston, C., Lewandowski, A. S., Williams, A. C. C., & Morley, S. (2010). Randomized controlled trials of psychological therapies for management of chronic pain in children and adolescents: An updated meta-analytic review. *Pain, 148*(3), 387–397.

Peterson, A. M., Nau, D. P., Cramer, J. A., Benner, J., Gwadry-Sridhar, F., & Nichol, M. (2007). A checklist for medication compliance and persistence studies using retrospective databases. *Value Health, 10*(1), 3-12.

Petrou, S., & Gray, A. (2011). Economic evaluation alongside randomised controlled trials: Design, conduct, analysis, and reporting. *BMJ, 342*. doi:10.1136/bmj.d1548

Piaggio, G., Elbourne, D. R., Pocock, S. J., Evans, S. J., Altman, D. G., & Group, C. (2012). Reporting of noninferiority and equivalence randomized trials: Extension of the CONSORT 2010 statement. *JAMA, 308*(24), 2594-2604.

Pijls, B. G., Dekkers, O. M., Middeldorp, S., Valstar, E. R., van der Heide, H. J. L., Van der Linden-Van der Zwaag, H. M. J., & Nelissen, R. G. H. H. (2011). AQUILA: Assessment of QUality in lower Limb Arthroplasty. An expert Delphi consensus for total knee and total hip arthroplasty. *BMC Musculoskelet Disord 12*, 173. Retrieved from <http://www.biomedcentral.com/1471-2474/12/173>

Pinson, A. G., Becker, D. M., Philbrick, J. T., & Parekh, J. S. (1991). Technetium-99m-RBC venography in the diagnosis of deep venous thrombosis of the lower extremity: A systematic review of the literature. *The Journal of Nuclear Medicine, 32*(12), 2324-2328.

Plonsky, L. (2014). Study quality in quantitative L2 research (1990–2010): A methodological synthesis and call for reform. *The Modern Language Journal, 98*(1), 450-470.

Plonsky , L., & Gass, S. (2011). Quantitative research methods, study quality, and outcomes: The case of interaction research. *Language Learning, 61*, 325–366.

Plonsky, L., & Gonulal, T. (2015). Methodological synthesis in quantitative L2 research: A review of reviews and a case study of Exploratory Factor Analysis [Supplemental material]. *Language Learning, 65*, 9-36. doi:10.1111/lang.12111

Pluye, P., Gagnon, M. P., Griffiths, F., & Johnson-Lafleur, J. (2009). A scoring system for appraising mixed methods research, and concomitantly appraising qualitative, quantitative and mixed methods primary studies in mixed studies reviews. *International Journal of Nursing Studies, 46*(4), 529–546.

Poldrack, R. A., Fletcher, P. C., Henson, R. N., Worsley, K. J., Brett, M., & Nichols, T. E. (2008). Guidelines for reporting an fMRI study. *Neuroimage, 40*(2), 409-414.

Popelut, A., Valet, F., Fromentin, O., Thomas, A., & Bouchard, P. (2010). Relationship between sponsorship and failure rate of dental implants: A systematic approach. *PLOS ONE, 5*(4). doi: 10.1371/journal.pone.0010274

Portell, M., Anguera, M. T., Chacón-Moscoso, S., & Sanduvete-Chaves, S. (2015). Guidelines for reporting evaluations based on observational methodology. *Psicothema, 27*(3), 283-289. doi: 10.7334/psicothema2014.276

Pretlove, S. J., Radley, S., Toozs-Hobson, P. M., Thompson, P. J., Coomarasamy, A., & Khan, K. S. (2006). Prevalence of anal incontinence according to age and gender: A systematic review and meta-regression analysis. *International Urogynecology Journal, 17*(4), 407-417.

Priebe, S., Richardson, M., Cooney., M., Adedeji, O., & McCabe, R. (2011). Does the therapeutic relationship predict outcomes of psychiatric treatment in patients with psychosis? A systematic review. *Psychotherapy and Psychosomatics, 80*, 70–77.

Ramsey, S., Willke, R., Briggs, A., Brown, R., Buxton, M., Chawla, A., . . . Reed, S. (2005). Good research practices for cost-effectiveness analysis alongside clinical trials: the ISPOR RCT-CEA Task Force report. *Value in Health, 8*(5), 521-533.

Randolph, A. G., Haynes, R. B., Wyatt, J. C., Cook, D. J., & Guyatt, G. H. (1999). Users' guides to the medical literature: XVIII. How to use an article evaluating the clinical impact of a computer-based clinical decision support system. *JAMA, 282*(1), 67-74.

Rangel, S. J., Kelsey, J., Colby, C. E., Anderson, J., & Moss, R. L. (2003). Development of a quality assessment scale for retrospective clinical studies in pediatric surgery. *Journal of Pediatric Surgery, 38*(3), 390–396.

Rao, W., Zhang, X., Zhang, J., Yan, R., Hu, Z., & Wang, Q. (2011). The role of nasogastric tube in decompression after elective colon and rectum surgery: A meta-analysis. *International Journal of Colorectal Disease, 26*(4), 423-429.

Rauch, F., Sievanen, H., Boonen, S., Cardinale, M., Degens, H., Felsenberg, D., . . . Rittweger, J. (2010). Reporting whole-body vibration intervention studies: Recommendations of the International Society of Musculoskeletal and Neuronal Interactions. *Journal of Musculoskeletal and Neuronal Interactions, 10*(3), 193-198.

Reveiz, L., Chan, A.-W., Krleza-Jeric, K., Granados, C. E., Pinart, M., Etxeandia, I., . . . Cardona, A. F. (2010). Reporting of methodologic information on trial registries for quality assessment: A study of trial records retrieved from the WHO search portal. *PLoS ONE, 5*(8). doi:10.1371/journal.pone.0012484

Richardson, T., Stallard, P., & Velleman, S. (2010). Computerised cognitive behavioural therapy for the prevention and treatment of depression and anxiety in children and adolescents: A systematic review. *Clinical Child and Family Psychology Review, 13*, 275–290.

Richardson, W. S., & Detsky, A. S. (1995a). Users' guides to the medical literature. VII. How to use a clinical decision analysis. A. Are the results of the study valid? *JAMA, 273*(16), 1292-1295.

Richardson, W. S., & Detsky, A. S. (1995b). Users' guides to the medical literature. VII. How to use a clinical decision analysis. B. What are the results and will they help me in caring for my patients? *JAMA, 273*(20), 1610-1613.

Richardson, W. S., Wilson, M. C., Guyatt, G. H., Cook, D. J., & Nishikawa, J. (1999). Users' guides to the medical literature: XV. How to use an article about disease probability for differential diagnosis. *JAMA, 281*(13), 1214-1219.

Richardson, W. S., Wilson, M. C., Williams, J. W. J., Moyer, V. A., & Naylor, C. D. (2010). Users' guides to the medical literature: XXIV. How to use an article on the clinical manifestations of disease. *JAMA, 284*(7), 869-875.

Ricós, C., Biosca, C., Ibarz, M., Minchinela, J., LLopis, M. A., Perich, C., . . . Vilanova, C. (2008). Quality indicators and specifications for strategic and support process in laboratory medicine. *Clinical Chemistry and Laboratory Medicine, 46*(8), 1189-1194.

Ridgewell, E., Dobson, F., Bach, T., & Baker, R. (2010). A systematic review to determine best practice reporting guidelines for AFO interventions in studies involving children with cerebral palsy. *Prosthetics and Orthotics International, 34*(2), 129-145.

Riley, R. D., Lambert, P. C., & Abo-Zaid, G. (2010). Meta-analysis of individual participant data: Rationale, conduct, and reporting. *BMJ, 340*. doi:10.1136/bmj.c221

Robb, S. L., Carpenter, J. S., & Burns, D. S. (2011). Reporting guidelines for music-based interventions. *Journal of Health Psychology, 16*(2), 342-352.

Ross, L. E., Grigoriadis, S., Mamisashvili, L., Koren, G., Steiner, M., Dennis, C. L., . . . Mousmanis, P. (2011). Quality assessment of observational studies in psychiatry: An example from perinatal psychiatric research. *International Journal of Methods in Psychiatric Research, 20*(4), 224–234.

Rowan, M., & Huston, P. (1997). Qualitative research articles: Information for authors and peer reviewers. *Canadian Medical Association Journal, 157*(10), 1442-1446.

Rozin, P. (2009). What kind of empirical research should we publish, fund and reward? A different perspective. *Perspectives on Psychological Science, 4*, 435-439. doi:10.1111/j.1745-6924.2009.01146.x

Rubino, M., & Pragnell, M. V. C. (1999). Guidelines for reporting case series of tumours of the colon and rectum. *Techniques in Coloproctology, 3*(2), 93-97.

Rubinstein, S. M., Pool, J. J. M., van Tulder, M. W., Riphagen, I. I., & De Vet, H. C. W. (2007). A systematic review of the diagnostic accuracy of provocative tests of the neck for diagnosing cervical radiculopathy. *European Spine Journal, 16*(3), 307-319.

Rud, B., Hilden, J., Hyldstrup, L., & Hróbjartsson, A. (2009). The Osteoporosis Self-assessment Tool versus alternative tests for selecting postmenopausal women for bone mineral density assessment: A comparative systematic review of accuracy. *Osteoporosis International, 20*(4), 599-607.

Rutherford, G. W., McFarland, W., Spindler, H., White, K., Patel, S. V., Aberle-Grasse, J., Sabin, K., Smith, N., Taché, S., Calleja-Garcia, J. M., & Stoneburner, R. L. (2010). Public health triangulation: Approach and application to synthesizing data to understand national and local HIV epidemics. *BMC Public Health, 10*. Retrieved from <http://www.biomedcentral.com/1471-2458/10/447>

Rutjes, A. W. S., Reitsma, J. B., Di Nisio, M., Smidt, N., van Rijn, J. C., & Bossuyt, P. M. M. (2006). Evidence of bias and variation in diagnostic accuracy studies. *Canadian Medical Association Journal, 174*(4), 469-476.

Sackett, D. L. (1989). Rules of evidence and clinical recommendations on the use of antithrombotic agents. *Chest, 95*, 2-4.

Saint-Raymond, A., Hill, S., Martines, J., Bahl, R., Fontaine, O., & Bero, L. (2010). CONSORT 2010. *The Lancet, 376*(9737), 229-230.

Salem, R., Lewandowski, R. J., Gates, V. L., Nutting, C. W., Murthy, R., Rose, S. C., . . . Interventional Oncology Task Force of the Society of Interventional Radiology (2011). Research reporting standards for radioembolization of hepatic malignancies. *Journal of Vascular and Interventional Radiology, 22*(3), 265-278.

Sánchez-Meca, J. (1997). Methodological issues in the meta-evaluation of correctional treatment. In S. Redondo, V. Garrido, J. Pérez, & R. Barberet (Eds.), *Advances in Psychology and Law. International contributions* (pp. 486-498). Berlin & New York: Walter de Gruyter.

Sánchez-Meca, J. & Ato, M. (1989). Meta-análisis: una alternativa metodológica a las revisiones tradicionales de la investigación [Meta-analysis: A methodological alternative to traditional reviews of research]. In J. Arnau, & H. Carpintero (Eds.), Tratado de psicología general. *Historia, teoría y método (Vol. 1)*. [Treatise of general psychology. History, theory and method] (pp. 617-669). Madrid, Spain: Alhambra.

Sanderson, S., Tatt, I. D., & Higgins, J. P. T. (2007). Tools for assessing quality and susceptibility to bias in observational studies in epidemiology: A systematic review and annotated bibliography. *International Journal of Epidemiology, 36*(3), 666-676.

Sargeant, J. M., Elgie, R., Valcour, J., Saint-Onge, J., Thompson, A., Marcynuk, P., & Snedeker, K. (2009). Methodological quality and completeness of reporting in clinical trials conducted in livestock species. *Preventive Veterinary Medicine, 91*, 107–115.

Sargeant, J. M., & O'Connor, A. M. (2014). Introduction to systematic reviews in animal agriculture and veterinary medicine. *Zoonoses and Public Health, 61*(Suppl. 1), 3–9.

Sargeant, J. M., Thompson, A., Valcour, J., Elgie, R., Saint-Onge, J., Marcynuk, P., & Snedeker, K. (2010). Quality of reporting of clinical trials of dogs and cats and associations with treatment effects. *Journal of Veterinary Internal Medicine, 24*, 44–50.

Sargeant, J. M., Torrence, M. E., Rajic, A., O’Connor, A. M., & Williams, J. (2006). Methodological quality assessment of review articles evaluating interventions to improve microbial food safety. *Foodborne Pathogens and Disease, 3*(4), 447-456.

Sarikaya, H., da Costa, B. R., Baumgartner, R. W., Duclos, K., Touzé, E., de Bray, J. M., . . . Jüni, P. (2013). Antiplatelets versus anticoagulants for the treatment of cervical artery dissection: Bayesian meta-analysis. *PLoS ONE, 8*(9). doi:10.1371/journal.pone.0072697

Scher, H. I., Eisenberger, M., D'Amico, A. V., Halabi, S., Small, E. J., Morris, M., . . . Soule, H. R. (2004). Eligibility and outcomes reporting guidelines for clinical trials for patients in the state of a rising prostate-specific antigen: Recommendations from the Prostate-Specific Antigen Working Group. *Journal of Clinical Oncology, 22*(3), 537-556.

Scherr, A. J. O., Lima, J. P. S. N., Sasse, E. C., Lima, C. S. P., & Sasse, A. D. (2011). Adjuvant therapy for locally advanced renal cell cancer: A systematic review with meta-analysis. *BMC Cancer, 11*, 115. doi:10.1186/1471-2407-11-115

Schriger, D. L. (2005). Suggestions for improving the reporting of clinical research: The role of narrative. *Annals of Emergency Medicine, 45*(4), 437-443.

Schulz, K. F., Altman, D. G., & Moher, D. (2010). CONSORT 2010 statement: updated guidelines for reporting parallel group randomised trials. *BMJ, 340*, 698-702.

Schulz, K. F., Chalmers, I., Hayes, R. J., & Altman, D. G. (1995). Empirical evidence of bias. Dimensions of methodological quality associated with estimates of treatment effects in controlled trials. *JAMA, 273*(5), 408-412.

Schünemann, H. J., Oxman, A. D., & Fretheim, A. (2006). Improving the use of research evidence in guideline development: 6. Determining which outcomes are important. *Health Research Policy and Systems, 4*(18). doi:10.1186/1478-4505-4-18

Segal, J., Eng, J., Jenckes, M. W., Tamariz, L. J., Bolger, D. T., Krishnan, J. A., . . . Bass, E. B. (2003). *Diagnosis and treatment of deep venous thrombosis and pulmonary embolism* (evidence report/technology assessment number 68). Rockville, MD: Agency for Healthcare Research and Quality.

Shadish, W. R. (2002). Revisiting field experimentation: Field notes for the future. *Psychological Methods, 7*(1), 3-18.

Shadish, W. R., & Heinsman, D. T. (1997). Experiments versus quasi-experiments: Do they yield the same answer? In W. J. Bukoski (Ed.), *Meta-analysis of drug abuse prevention programs* (pp. 147-164). Washington DC: Superintendent of Documents.

Shadish, W. R., & Ragsdale, K. (1996). Random versus nonrandom assignment in controlled experiments: Do you get the same answer? *Journal of Consulting and Clinical Psychology, 64*(6), 1290-1305.

Shaneyfelt, T. M., Mayo-Smith, M. F., & Rothwangl, J. (1999). Are guidelines following guidelines? The methodological quality of clinical practice guidelines in the peer-reviewed medical literature. *JAMA, 281*(20), 1900-1905.

Shea, B. J., Grimshaw, J. M., Wells, G. A., Boers, M., Andersson, N., Hamel, C., & Bouter, L. M. (2007). Development of AMSTAR: A measurement tool to assess the methodological quality of systematic reviews. *BMC Medical Research Methodology, 7*, 10. Retrieved from <http://www.biomedcentral.com/1471-2288/7/10>

Shea, B. J., Hamel, C., Wells, G. A., Bouter, L. M., Kristjansson, E., Grimshaw, J., . . . Boers, M. (2009). AMSTAR is a reliable and valid measurement tool to assess the methodological quality of systematic reviews. *Journal of Clinical Epidemiology, 62*, 1013-1020.

Shekelle, P. G., Woolf, S. H., Eccles, M., & Grimshaw, J. (1999). Clinical guidelines: Developing guidelines. *BMJ, 318*, 593-596.

Sherman, L. W., Gottfredson, D., MacKenzie, D., Eck, J., Reuter, P., & Bushway, S. (1998). *Preventing crime: What works, what doesn’t, what’s promising: A report to the United States Congress, prepared for the National Institute of Justice*. Baltimore, MD: University of Maryland, Department of Criminology and Criminal Justice.

Sherrington, C., Herbert, R. D., Maher, C. G., & Moseley, A. M. (2000). PEDro. A database of randomized trials and systematic reviews in physiotherapy. *Manual Therapy, 5*, 223–226.

Shiffman, R. N., Dixon, J., Brandt, C., Essaihi, A., Hsiao, A., Michel, G., & O'Connell, R. (2005). The GuideLine Implementability Appraisal (GLIA): Development of an instrument to identify obstacles to guideline implementation. *BMC Medical Informatics and Decision Making, 5*, 23. Retrieved from <http://www.biomedcentral.com/1472-6947/5/23>

Siddiqui, M. R., [Sajid, M. S](http://www.ncbi.nlm.nih.gov/pubmed/?term=Sajid%20MS%5BAuthor%5D&cauthor=true&cauthor_uid=20714895)., [Khatri, K](http://www.ncbi.nlm.nih.gov/pubmed/?term=Khatri%20K%5BAuthor%5D&cauthor=true&cauthor_uid=20714895)., [Cheek, E](http://www.ncbi.nlm.nih.gov/pubmed/?term=Cheek%20E%5BAuthor%5D&cauthor=true&cauthor_uid=20714895)., & [Baig, M. K](http://www.ncbi.nlm.nih.gov/pubmed/?term=Baig%20MK%5BAuthor%5D&cauthor=true&cauthor_uid=20714895). (2010). Elective open versus laparoscopic sigmoid colectomy for diverticular disease: A meta-analysis with the sigma trial. *World Journal of Surgery, 34*(12), 2883-2901.

Simel, D. L., Rennie, D., & Bossuyt, P. M. (2008). The STARD statement for reporting diagnostic accuracy studies: Application to the history and physical examination. *Journal of General Internal Medicine, 23*(6), 768-774.

Simon, L., & Lewis, S. E. M. (2011). Basic and clinical aspects of sperm comet assay. In A. Zini, & A. Agarwal (Eds.), *Sperm chromatin. Biological and clinical applications in male infertility and assisted reproduction* (pp. 217-232). New York, NY: Springer.

Sindhu, F., Carpenter, L., & Seers, K. (1997). Development of a tool to rate the quality assessment of randomized controlled trials using a Delphi technique. *Journal of Advanced Nursing, 25*, 1262-1268.

Siontis, G. C. M., Patsopoulos, N. A., Vlahos, A. P., & Ioannidis, J. P. A. (2010). Selection and presentation of imaging figures in the medical literature. *PLoS ONE, 5*(5). doi:10.1371/journal.pone.001088

Skapinakis, P., & Athanasiou, T. (2010). Study design, statistical inference and literature search in surgical research. In T. Athanasiou (Ed.), *Key topics in surgical research and methodology*(pp. 33-53). Verlag-Berlin-Heidelberg: Springer.

Skoetz, N., Trelle, S., Rancea, M., Haverkamp, H., Diehl, V., Engert, A., & Borchmann, P. (2013). Effect of initial treatment strategy on survival of patients with advanced-stage Hodgkin’s lymphoma: A systematic review and network meta-analysis. *The Lancet Oncology, 14*, 943–952.

Slater, N. J., Hansson, B. M. E., Hendriks, T., & Bleichrodt, P. (2011). Repair of parastomal hernias with biologic grafts: A systematic review. *Journal of Gastrointestinal Surgery, 15*(7), 1252-1258.

Slatkovska, L., Alibhai, S. M. H., Beyene, J., & Cheung, A. M. (2010). Effect of whole-body vibration on BMD: A systematic review and meta-analysis. *Osteoporosis International, 21*(12), 1969-1980.

Slim, K., Nini, E., Forestier, D., Kwiatkowski, F., Panis, Y., & Chipponi, J. (2003). Methodological index for non-randomized studies (MINORS): Development and validation of a new instrument. *ANZ Journal of Surgery, 73*, 712–716.

Soares, M. O., Welton, N. J., Harrison, D. A., Peura, P., Shankar-Hari, M., Harvey, S. E., . . . Rowan, K. M. (2012). An evaluation of the feasibility, cost and value of information of a multicentre randomised controlled trial of intravenous immunoglobulin for sepsis (severe sepsis and septic shock): Incorporating a systematic review, meta-analysis and value of information analysis. *Health Technology Assessment, 16*(7), 1-186.

Sockol, L. E. (2015). A systematic review of the efficacy of cognitive behavioral therapy for treating and preventing perinatal depression. *Journal of Affective Disorders, 177*, 7-21.

Sorinola, O., Olufowobi, O., Coomarasamy, A., & Khan, K. S. (2004). Instructions to authors for case reporting are limited: A review of a core journal list. *BMC Medical Education, 4*, 4. Retrieved from <http://www.biomedcentral.com/1472-6920/4/4>

Spinewine, A., Claeys, C., Foulon, V., & Chevalier, P. (2013). Approaches for improving continuity of care in medication management: A systematic review. *International Journal for Quality in Health Care, 25*(4), 403–417.

Staquet, M., Berzon, R., Osoba, D., & Machin, D. (1996). Guidelines for reporting results of quality of life assessments in clinical trials. *Quality of Life Research, 5*(5), 496-502.

Sterne, J. A., White, I. R., Carlin, J. B., Spratt, M., Royston, P., Kenward, M. G., . . . Carpenter, J. R. (2009). Multiple imputation for missing data in epidemiological and clinical research: Potential and pitfalls. *BMJ, 338*. doi:10.1136/bmj.b2393

Steuten, L. M. G., Vrijhoef, H. J. M., van Merode, G. G., Severens, J. L., & Spreeuwenberg, C. (2004). The Health Technology Assessment-Disease Management instrument reliably measured methodologic quality of health technology assessments of disease management. *Journal of Clinical Epidemiology, 57*, 881–888.

Stevenson, M., Gomersall, T., Lloyd Jones, M., Rawdin, A., Hernández, M., Dias, S., . . . Rees, A. (2014). Percutaneous vertebroplasty and percutaneous balloon kyphoplasty for the treatment of osteoporotic vertebral fractures: a systematic review and cost-effectiveness analysis. *Health Technology Assessment, 18*(17), 1-289.

Stiles, C. R., Biondo, P. D., Cummings, G., & Hagen, N. A. (2010). Clinical trials focusing on cancer pain educational interventions: Core components to include during planning and reporting. *Journal of Pain and Symptom Management, 40*(2), 301-308.

Stock-Schroer, B., Albrecht, H., Betti, L., Endler, P. C., Linde, K., Ludtke, R., . . . Baumgartner, S. (2009). Reporting Experiments in Homeopathic Basic Research (REHBaR) - a detailed guideline for authors. *Homeopathy, 98*(4), 287-298.

Stone, A. A., & Shiffman, S. (2002). Capturing momentary, self-report data: a proposal for reporting guidelines. *Annals of Behavioral Medicine, 24*(3), 236-243.

Stone, S. P., Cooper, B. S., Kibbler, C. C., Cookson, B. D., Roberts, J. A., Medley, G. F., . . . Davey, P. G. (2007). The ORION statement: guidelines for transparent reporting of Outbreak Reports and Intervention studies Of Nosocomial infection. *Journal of Antimicrobial Chemotherapy, 59*(5), 833-840.

Stout, N. K., Knudsen, A. B., Kong, C. Y., McMahon, P. M., & Gazelle, G. S. (2009). Calibration methods used in cancer simulation models and suggested reporting guidelines. *PharmacoEconomics, 27*(7), 533-545.

Strom, J. B., Wimmer, N. J., Wasfy, J. H., Kennedy, K., & Yeh, R. W. (2014). Association between operator procedure volume and patient outcomes in percutaneous coronary intervention. A systematic review and meta-analysis. *Circulation: Cardiovascular Quality and Outcomes, 7*, 560-566.

Stroup, D. F., Berlin, J. A., Morton, S. C., Olkin, I., Williamson, G. D., Rennie, D., . . . Thacker, S. B. (2000). Meta-analysis of observational studies in epidemiology: A proposal for reporting. *JAMA, 283*, 2008-2012.

Subramanian, J., & Simon, R. (2010). Gene expression-based prognostic signatures in lung cancer: Ready for clinical use? *Journal of the National Cancer Institute, 102*(7), 464-474.

Sutton, A. J., Abrams, K. R., Jones, D. R., Sheldon, T. A., & Sing, F. (2000). Study quality. In A. J. Sutton, K. R. Abrams, D. R. Jones, T. A. Sheldon, & F. Sing (Eds.), *Methods for meta-analysis in medical research*(pp. 133-146). Chichester, NY: Wiley Series in Probability and Statistics.

Sweat, M. D., Denison, J., Kennedy, C., Tedrow, V., & O’Reilly, K. (2012). Effects of condom social marketing on condom use in developing countries: A systematic review and meta-analysis, 1990–2010. *Bulletin of the World Health Organization, 90*, 613–622.

Taji, Y., Kuwahara, T., Shikata, S., & Morimoto, T. (2006). Meta-analysis of antiplatelet therapy for IgA nephropathy. *Clinical and Experimental Nephrology, 10*(4), 268-273.

Talmon, J., Ammenwerth, E., Brender, J., de Keizer, N., Nykanen, P., & Rigby, M. (2009). STARE-HI - STAtement on Reporting of Evaluation studies in Health Informatics. *International Journal of Medical Informatics, 78*(1), 1-9.

Tam, K. W., Wei, P. L., Kuo, L. J., & Wu, C. H. (2010). Systematic review of the use of a mesh to prevent parastomal hernia. *World Journal of Surgery, 34*(11), 2723-2729.

Tang, B. M., Huang, S. J., & McLean, A. S. (2010). Genome-wide transcription profiling of human sepsis: A systematic review. *Critical Care, 14*(6). doi:10.1186/cc9392

Ter Riet, G., Kleijnen, J., & Knipschild, P. (1990). Acupuncture and chronic pain: A criteria based meta­analysis. *Journal of Clinical Epidemiology, 43*, 1191­1199.

Terwee, C. B., Mokkink, L. B., Knol, D. L., Ostelo, R. W. J. G., Bouter, L. M., & de Vet, H. C. W. (2012). Rating the methodological quality in systematic reviews of studies on measurement properties: A scoring system for the COSMIN checklist. *Quality of Life Research, 21*(4), 651-657.

The Asilomar Working Group on Recommendations for Reporting of Clinical Trials in the Biomedical Literature (1996). Checklist of information for inclusion in reports of clinical trials. *Annals of Internal Medicine, 124*, 741-743.

The Joanna Briggs Institute (2004). *System for the Unified MAnagement of the Review and Assessment of Information (SUMARI)*. Retrieved from http://joannabriggs.org/sumari.html

Thomas, B. H., Ciliska, D., Dobbins, M., & Micucci, S. (2004). A process for systematically reviewing the literature: Providing the research evidence for public health nursing interventions. *Worldviews on Evidence-Based Nursing, 1*(3), 176-184.

Timmer, A., Sutherland, L. R., & Hilsden, R. J. (2003). Development and evaluation of a quality score for abstracts. *BMC Medical Research Methodology, 3*, 2. Retrieved from <http://www.biomedcentral.com/1471-2288/3/2>

Tompa, E., Dolinschi, R., de Oliveira, C., Amick III, B. C., & Irvin, E. (2010). A systematic review of workplace ergonomic interventions with economic analyses. *Journal of Occupational Rehabilitation, 20*, 220–234.

Tong, A., Flemming, K., McInnes, E., Oliver, S., & Craig, J. (2012). ENhancing Transparency in REporting the synthesis of Qualitative research: ENTREQ. *BMC Medical Research Methodology, 12*(1), 181. Retrieved from <http://www.biomedcentral.com/1471-2288/12/181>

Tong, A., Sainsbury, P., & Craig, J. (2007). COnsolidated criteria for REporting Qualitative research (COREQ): a 32-item checklist for interviews and focus groups. *Journal for Quality in Health Care, 19*(6), 349-357.

Tong, Y.-S., Wu, C.-C., Bai, C.-H., Lee, H.-C., Liang, H.-H., Kuo, L.-J., . . . Tam, K.-W. (2014). Effect of extraperitoneal bupivacaine analgesia in laparoscopic inguinal hernia repair: A meta-analysis of randomized controlled trials. *Hernia, 18*, 177–183.

Tooth, L., Ware, R., Bain, C., Purdie, D. M., & Dobson, A. (2005). Quality of reporting of observational longitudinal research. *American Journal of Epidemiology, 161*, 280-288.

Tran, D. Q., Duong, S., & Finlayson, R. J. (2010). Lumbar spinal stenosis: A brief review of the nonsurgical management. *Canadian Journal of Anesthesia, 57*, 694–703.

Tritchler, D. (1999). Modelling study quality in meta-analysis. *Statistics in Medicine, 18*(16), 2135-2145.

Tullar, J. M., Brewer, S., Amick III, B. C., Irvin, E., Mahood, Q., Pompeii, L. A., . . . Evanoff, B. (2010). Occupational safety and health interventions to reduce musculoskeletal symptoms in the health care sector. *Journal of Occupational Rehabilitation, 20*, 199–219.

Turina, M. I., Shennib, H., Dunning, J., Cheng, D., Martin, J., Muneretto, C., . . . Sergeant, P. T. (2009). EACTS/ESCVS committee. EACTS/ESCVS best practice guidelines for reporting treatment results in the thoracic aorta. *European Journal of Cardio-thoracic Surgery, 35*(6), 927-930.

Turlik, M. A., & Kushner, D. (2000). Levels of evidence of articles in podiatric medical journals. *Journal of the American Podiatric Medical Association, 90*(6), 300-302.

Turner, E. L., Dobson, J. E., & Pocock, S. J. (2010). Categorisation of continuous risk factors in epidemiological publications: A survey of current practice. *Epidemiologic Perspectives & Innovations, 7*. doi:10.1186/1742-5573-7-9

Vale, L., Thomas, R., MacLennan, G., & Grimshaw, J. (2007). Systematic review of economic evaluations and cost analyses of guideline implementation strategies. *The European Journal of Health Economics, 8*(2), 111-121.

Valentine, J. C., & Cooper, H. (2008). A systematic and transparent approach for assessing the methodological quality of intervention effectiveness research: The study Design and Implementation Assessment Device (study DIAD). *Psychological Methods, 13*(2), 130-149.

Valentine, J. C., & McHugh, C. M. (2007). The effects of attrition on baseline comparability in randomized experiments in education: A meta-analysis. *Psychological Methods, 12*(3), 268-282.

van Abbema, R., Lakke, S. E., Reneman, M. F., van der Schans, C. P., van Haastert, C. J. M., Geertzen, J. H. B., & Wittink, H. (2011). Factors associated with functional capacity test results in patients with non-specific chronic low back pain: A systematic review. *Journal of Occupational Rehabilitation, 21*, 455–473.

van der Heijden, G. J., van der Windt, D. A., Kleijnen, J., Koes, B. W., & Bouter, L. M. (1996). Steroid injections for shoulder disorders: A systematic review of randomized clinical trials. *British Journal of General Practice, 46*, 309-316.

van Tulder, M. W., Koes, B. W., & Bouter, L. M. (1997). Conservative treatment of acute and chronic nonspecific low back pain. A systematic review of randomized controlled trials of the most common interventions. *Spine, 22*, 2128-2156.

Verhagen, A. P., de Vet, H. C. W., de Bie, R. A., Alphons G. H., Kessels, A. G. H., Boers, M., Bouter, L. M., & Knipschild, P. G. (1998). The Delphi list: A criteria list for quality assessment of randomized clinical trials for conducting systematic reviews developed by Delphi consensus. *Journal of Clinical Epidemiology,* 51, 1235-41.

Vest, J. R., Gamm, L. D., Oxford, B. A., & Gonzalez, M. I. (2010). Determinants of preventable readmissions in the United States: A systematic review. *Implementation Science, 5*, 88. Retrieved from <http://www.implementationscience.com/content/5/1/88>

Vickers, A. J., Ballen, V., & Scher, H. I. (2007). Setting the bar in phase II trials: The use of historical data for determining “go/no go” decision for definitive phase III testing. *Clinical Cancer Research, 13*(3), 972-976.

Vintzileos, A. M., & Beazoglou, T. (2004). Design, execution, interpretation, and reporting of economic evaluation studies in obstetrics. *American Journal of Obstetrics and Gynecology, 191*(4), 1070-1076.

Virués-Ortega, J., & Moreno-Rodríguez, R. (2008). Guidelines for clinical case reports in behavioral clinical psychology. *International Journal of Clinical and Health Psychology, 8*(3), 765-777.

Viswanathan, M., Ansari, M. T., Berkman, N. D., Chang, S., Hartling, L., McPheeters, M., . . . Treadwell, J. R. (2012). *Assessing the risk of bias of individual studies in systematic reviews of health care interventions. Methods guide for effectiveness and comparative effectiveness reviews [Internet]*. Rockville, MD: Agency for Healthcare Research and Quality. Retrieved from http://effectivehealthcare.ahrq.gov/ehc/products/322/998/MethodsGuideforCERs_Viswanathan_IndividualStudies.pdf

Vitek, J. L., Lyons, K. E., Bakay, R., Benabid, A. L., Deuschl, G., Hallett, M., . . . Lang, A. E. (2010). Standard guidelines for publication of deep brain stimulation studies in Parkinson's disease (Guide4DBS-PD). *Movement Disorders, 25*(11), 1530-1537.

Vlaanderen, J., Vermeulen, R., Heederik, D., & Kromhout, H. (2008). Guidelines to evaluate human observation studies for quantitative risk assessment. *Environmental Health Perspectives, 116*, 1700–1705.

Von Elm, E., Altman, D. G., Egger, M., Pocock, S. J., Gøtzsche, P. C., & Vandenbroucke, J. P. (2007). The Strengthening the Reporting of Observational Studies in Epidemiology (STROBE) statement. Guidelines for reporting observational studies. *Epidemiology, 18*(6), 800-804.

Wang, R., Lagakos, S. W., Ware, J. H., Hunter, D. J., & Drazen, J. M. (2007). Statistics in medicine-reporting of subgroup analyses in clinical trials. *New England Journal of Medicine, 357*(21), 2189-2194.

Wardman, M. (2012). Review and meta-analysis of U.K. time elasticities of travel demand. *Transportation, 39*, 465–490.

Watt, E., Arnold, C., & Sayre, J. (2010). Evaluation. In A. A. T. Bui, & R. K. Taira (Eds.), *Medical Imaging Informatics* (pp. 403-438). New York. NY: Springer Science.

Webster, J. D., Dennis, M. M., Dervisis, N., Heller, J., Bacon, N. J., Bergman, P. J., . . . Kiupel, M. (2011). Recommended guidelines for the conduct and evaluation of prognostic studies in veterinary oncology. *Veterinary Pathology, 48*(1), 7-18.

Weijenberg, A., Offringa, M., Brouwer, O. F., & Callenbach, P. M. C. (2010). RCTs with new antiepileptic drugs in children: A systematic review of monotherapy studies and their methodology. *Epilepsy Research, 91*, 1-9.

Weisz, J. R., Hawley, K. M., Pilkonis, P. A., Woody, S. R., & Follette, W. C. (2000). Stressing the (other) three Rs in the search for empirically supported treatments: Review procedures, research quality, relevance to practice and the public interest. *Clinical Psychology: Science and Practice, 7*(3), 243-258.

Welch, R. W., Antoine, J. M., Berta, J. L., Bub, A., de Vries, J., Guarner, F., . . . Woodside, J. V. (2011). Guidelines for the design, conduct and reporting of human intervention studies to evaluate the health benefits of foods. *The British Journal of Nutrition, 106*(Suppl. 2), 3-15.

Welch, V., Petticrew, M., Tugwell, P., Moher, D., O'Neill, J., Waters, E.,… the PRISMA-Equity Bellagio group (2012). PRISMA-Equity 2012 extension: Reporting guidelines for systematic reviews with a focus on health equity. *PLoS Medicine, 9*(10). doi:10.1371/journal.pmed.1001333

Wells, G., Shea, B., O’Connell, D., Robertson, J., Peterson, J., Welch, V., . . . Tugwell, P. (2009). *The Newcastle-Ottawa Scale (NOS) for assessing the quality of nonrandomized studies in meta-analysis*. Retrieved from http://www.ohri.ca/programs/clinical_epidemiology/oxford.htm

West, S., King, V., Carey, T. S., Lohr, K. N., McKoy, N., Sutton, S. F., & Lux, L. (2002). *Systems to rate the strength of scientific evidence*. Rockville, MD: Agency for Healthcare Research and Quality.

White, A. (2005). Conducting and reporting case series and audits-author guidelines for acupuncture in medicine. *Acupuncture in Medicine, 23*(4), 181-187.

Whiting, P. F., Rutjes, A. W. S., Reitsma, J. B., Bossuyt, P. M. M., & Kleijnen, J. (2003). The development of QUADAS: A tool for the quality assessment of studies of diagnostic accuracy included in systematic reviews. *BMC Medical Research Methodology, 3*, 25.

Whiting, P. F., Rutjes, A. W. S., Westwood, M. E., Mallett, S., Deeks, J. D., Reitsma, J. B., . . . the QUADAS-2 Group (2011). QUADAS-2: A revised tool for the QUality Assessment of Diagnostic Accuracy Studies. *Annals of Internal Medicine, 155*, 529-536.

Widmann, G., Stoffner, R., Sieb, M., & Bale, R. (2009). Target registration and target positioning errors in computer-assisted neurosurgery: Proposal for a standardized reporting of error assessment. *International Journal of Medical Robotics and Computer Assisted Surgery,* *5*(4), 355-365.

Wilkinson, C. P. (1999). Evidence-based medicine regarding the prevention of retinal detachment. *Transactions of the American Ophthalmological Society, 97*, 397-406.

Wilson, D. B. (2009). Missing a critical piece of the pie: simple document search strategies inadequate for systematic reviews. *Journal of Experimental Criminology, 5*(4), 429-440.

Wilson, M. C., Hayward, R. S., Tunis, S. R., Bass, E. B., & Guyatt, G. (1995). Users' guides to the Medical Literature. VIII. How to use clinical practice guidelines. B. what are the recommendations and will they help you in caring for your patients? *JAMA, 274*(20), 1630-1632.

Wolfe, F., Lassere, M., van der Heijde. D., Stucki, G., Suarez-Almazor, M., Pincus, T., . . . Boers, M. (1999). Preliminary core set of domains and reporting requirements for longitudinal observational studies in rheumatology. *Journal of Rheumatology, 26*(2), 484-489.

Wong, G., Greenhalgh, T., Westhorp, G., Buckingham, J., & Pawson, R. (2013a). RAMESES publication standards: Meta-narrative reviews. *BMC Medicine, 11*, 20. Retrieved from <http://www.biomedcentral.com/1741-7015/11/20>

Wong, G., Greenhalgh, T., Westhorp, G., Buckingham, J., & Pawson, R. (2013b). RAMESES publication standards: Realist syntheses. *BMC Medicine, 11*, 21. Retrieved from <http://www.biomedcentral.com/1741-7015/11/21>

Wortman, P. M. (1994). Judging research quality. In H. Cooper, & L. V. Hedges (Eds.), *The handbook of research synthesis*(pp. 97-109). New York, NY: Russell Sage Foundation.

Wu, M.-Y., Hsiang, H.-F., Wong, C. S., Yao, M. S., Li, Y. W., Hsiang, C. Y., . . . Tam, K. W. (2013). The effectiveness of N-acetylcysteine in preventing contrast-induced nephropathy in patients undergoing contrast-enhanced computed tomography: A meta-analysis of randomized controlled trials. *International Urology and Nephrology, 45*, 1309–1318.

Wu, T., Shang, H., Bian, Z., Zhang, J., Li, T., Li, Y., & Zhang, B. (2010). Recommendations for reporting adverse drug reactions and adverse events of traditional Chinese medicine. *Journal of Evidence-Based Medicine, 3*(1), 11-17.

Xu, Y. (2008). Methodological issues and challenges in data collection and analysis of qualitative meta-synthesis. *Asian Nursing Research, 2*(3), 173–183.

Yajun, W., Yue, Z., Xiuxin, H., & Cui, C. (2010). A meta-analysis of artificial total disc replacement versus fusion for lumbar degenerative disc disease. *European Spine Journal, 19*, 1250–1261.

Yeaton, W. H., Langenbrunner, J. C., Smyth, J. M., & Wortman, P. M. (1995). Exploratory research synthesis. Methodological considerations for addressing limitations in data quality. *Evaluation & the Health Professions, 18*(3), 283-303.

Zakrzewska, J. M., & Lopez, B. C. (2003). Quality of reporting in evaluations of surgical treatment of trigeminal neuralgia: Recommendations for future reports. *Neurosurgery, 53*(1), 110-120.

Zaritsky, A., Nadkarni, V., Hazinski, M. F., Foltin, G., Quan, L., Wright, J., . . . Chameides, L. (1995). Recommended guidelines for uniform reporting of pediatric advanced life support: The pediatric Utstein style. *Annals of Emergency Medicine, 26*(4), 487-503.

Zaza, S., Wright-De Agüero, L. K., Briss, P. A., Truman, B. I., Hopkins, D. P., Hennessy, M. H., . . . Task Force on Community Preventive Services (2000). Data collection instrument and procedure for systematic reviews in the Guide to Community Preventive Services. *American Journal of Preventive Medicine, 18*, 44-74.

Zhang, H., Lu, Y., Liu, M., Zou, Z., Wang, L., Xu, F. Y., & Shi, X. Y. (2013). Strategies for prevention of postoperative delirium: A systematic review and meta-analysis of randomized trials. *Critical Care, 17*. Retrieved from <http://ccforum.com/content/17/2/R47>

Zhang, L. N., Sun, J. P., Xue, X. Y., & Wang, J. X. (2013). Exogenous pulmonary surfactant for acute respiratory distress syndrome in adults: A systematic review and meta-analysis. *Experimental and Therapeutic Medicine, 5*(1), 237-242.

Zhang, Y., Zhang, J., Tian, C., Xiao, Y., He, C., Li, X., . . . Fan, H. (2011). The −308 G/A polymorphism in TNF-α gene is associated with asthma risk: An update by meta-analysis. *Journal of Clinical Immunology, 31*(2), 174-185.

Zhao, L., & Bracken, M. B. (2011). Association of *CD14 -260* (-159) C>T and asthma: A systematic review and meta-analysis. *BMC Medical Genetics, 10*(11), 1524–1529.

Zwarenstein, M., Treweek, S., Gagnier, J. J., Altman, D. G., Tunis, S., Haynes, B., . . . Pragmatic Trials in Healthcare (Practihc) group (2008). Improving the reporting of pragmatic trials: An extension of the CONSORT statement. *BMJ, 337*. doi:10.1136/bmj.a2390
