# Supplementary material for: The Methodological Quality Scale (MQS) for intervention programs: validity evidence
Source: Front Psychol. 2023 Jul 6;14:1217661. doi: 10.3389/fpsyg.2023.1217661 (PMC10358327; doi:10.3389/fpsyg.2023.1217661)
Supplement: Supplementary file 2 [file Data_Sheet_2.DOCX]

Supplementary Material

The Methodological Quality Scale (MQS) for Intervention Programs: Validity Evidence

**Salvador Chacón-Moscoso*, Susana Sanduvete-Chaves, José Antonio Lozano-Lozano, Francisco Pablo Holgado-Tello**

*** Correspondence:** Salvador Chacón Moscoso: schacon@us.es

# Supplementary Data S2

**CODING MANUAL** (adapted from Chacón-Moscoso, S., Sanduvete-Chaves, S., & Sánchez-Martín, M. (2016). The development of a checklist to enhance methodological quality in intervention programs. *Frontiers in Psychology,* *7*:1811. https://doi.org/10.3389/fpsyg.2016.01811)

1. **Inclusion and exclusion criteria for the units provided**: explicit reasons provided as to why certain people were able to participate in the study and others were not:
2. **No:** no explicit selection criteria for units AND with exceptions in their application; information unavailable.

**0.5. Intermediate:** explicit selection criteria for units OR applied to all potential participants.

**1. Yes (replicable):** explicit selection criteria for units AND applied to all potential participants.

1. **Methodology or design:** something an experimenter could manipulate or control in an experiment to help address a threat to validity (Shadish et al., 2002, p. 507):
2. **Pre-experimental/others** (questionnaires/observational/naturalistic) (Olivares et al., 2000; Sánchez-Meca, 1997): a study with only one group and a maximum of two measurement occasions for the same dependent variable (e.g., pre-post design); or when there are two groups and only one measure (e.g., control-experimental design).
   1. **Quasi-experimental** (two groups without randomized assignment) non-equivalent control groups with pre-test and post-test; or one group with three or more measures of the same dependent variable (even without pretest): an experiment (exploration of the effects of manipulating a variable; Shadish et al., 2002, p. 507) in which units are not randomly assigned to conditions (Shadish et al., 2002, p. 511).
   2. **Experimental; randomized:** an experiment (exploration of the effects of manipulating a variable; Shadish et al., 2002, p. 507) in which units are randomly assigned to conditions (Shadish et al., 2002, p. 511).
3. **Attrition:** loss of units. In randomized experiments, this refers to loss that occurred after the random assignment (Shadish et al., 2002, p. 505); i.e., the number of participants from the initial sample that did not conclude the study (e.g., N pre minus N post).
4. **Unspecified:** information is not available and cannot be calculated AND reasons for loss of units are not specified.

**0.5. Intermediate:** number of units lost is specified or can be calculated OR reasons for loss of units are specified.

**1. Specified:** no units are lost, or number of units lost is specified or can be calculated AND reasons for loss of units are specified.

1. **Attrition between groups:** this item evaluated the differences in attrition between two groups.

**0. Unspecified:** information is not available and cannot be calculated AND reasons for attrition between groups are not specified.

**0.5. Intermediate:** number of lost units is specified or can be calculated OR reasons for attrition between groups are specified.

**1. Specified:** no units were lost, or number of lost units is specified or can be calculated AND reason/s for the attrition between groups is/are specified.

**9.** **Not applicable:** no cross-group comparison.

1. **Exclusions after assignment (specify percentage):** this is also called post-assignment attrition and includes cases in which, after a participant assignment to a condition, an experimenter deliberately dropped that participant from the data (Shadish et al., 2002, p. 323).
2. **Unjustified exclusion and risk of bias:** participants were excluded deliberately even though the intervention given could have influenced the ultimate decision regarding exclusion, as may occur with excessively broad eligibility criteria (Fergusson et al., 2002).
   1. **Justified exclusion and risk of bias:** The post-assignment attrition can be attributed to errors made by personnel in assessing eligibility, or participants never received the intervention, BUT there was not a proper evaluation of the potential bias introduced.
3. **Justified or non-justified exclusion, no risk of bias:** There was no post-assignment attrition, or it was due to errors made by personnel in assessing eligibility, or participants never received the intervention, AND an independent, blinded adjudication committee evaluated all randomized patients and concluded that no bias was introduced (or potential bias was evaluated appropriately some other way)– (Fergusson et al., 2002).
4. **Follow-up period**: the amount of time between the first post-intervention measurements and any additional measurements. When the study presented more than one follow-up period, the longest was considered (Fleischman et al., 2010).
5. No follow-up or less than two months.

**0.5.** Between two and six months (both included).

- 1. More than six months.

1. **Measurement occasions for each dependent variable** (Olivares et al., 2000): this item specified when the measurements were taken.
2. **Post-intervention only:** all measurements were taken after the intervention.

**0.5. Pre- and post-intervention:** some measurements were taken before and immediately after the intervention.

1. **Pre-, post-intervention and follow-up period:** some measurements were taken before, immediately after the intervention, and again at a later date.
2. **Measures in pre-test appear in post-test** (Olivares et al., 2000): this considers the number of measurements of the dependent variable taken before the intervention and for the remaining measurement occasions.
3. **None:** none of the measurements taken in pretest was repeated on another measurement occasion.

**0.5. Some:** at least one of the measurements taken in pretest was taken at all measurement occasions.

1. **All** (Olivares et al., 2000): all measurements taken in pretest were taken at all the measurement occasions.

**9. Not applicable:** no pre and posttest measurements.

1. **Standardization of the dependent variables:** level of normalization of the tool to measure the variable that varied in response to the independent variable (also called effect or outcome) (Shadish et al., 2002, p. 507).
2. **Low standardization (self-reports and post hoc records)** (Olivares et al., 2000): all measurements were taken using ad hoc tools, developed in a specific situation, and without any study of their psychometric properties.

**0.5. Medium standardization** (Anguera et al., 2008): at least one measurement was taken using structured tools with ONE study of their psychometric properties (reliability or one form of validity evidence).

1. **High standardization** (Olivares et al., 2000): at least one measurement was taken using structured tools. At least TWO studies of their psychometric properties (reliability, validity, construction of scaling) were carried out.
2. **Control techniques** (Olivares et al., 2000):
3. **None:** no control technique is specified or described.
   1. **Masking OR other/s:** masking, also known as blinding, refers to a procedure that prevented participants and/or experimenters from knowing the hypotheses (Shadish et al., 2002, p. 78) OR any other control technique was used (e.g., matching, stratifying, counterbalancing, constant, participant as own experimental control -longitudinal-).
   2. **Masking AND other:** masking AND at least one other control technique.
4. **Construct definition of outcome**: explanation of the concept, model, or schematic idea measured as a dependent variable (Shadish et al., 2002, p. 506):
5. **No definition:** no concept treated as a dependent variable was defined in a conceptual or empirical way.
   1. **Vague definition:** at least one concept treated as a dependent variable was defined in a conceptual and/or empirical way.
6. **Replicable by reader in own setting:** all concepts treated as dependent variables were defined in a conceptual and empirical way.
7. **Statistical methods for imputing missing data**: to estimate what the study would have yielded had there been no attrition (Shadish et al., 2002, p. 337):
8. **High risk:** it is not clear if there was attrition, or there was attrition and calculations to estimate effects were carried out without imputing missing data.

**0.5. Medium risk:** values for the missing data points were imputed so they could be included in the analyses. The method used was specified, i.e., sample mean substitution, last value forward method for longitudinal data sets, hot deck imputation, single imputation (e.g., imputation, regression imputation), or multiple imputation (e.g., likelihood ratio test after multiple imputation). The reasons for choosing the specific method were not specified.

1. **Low risk:** there was no attrition or values for the missing data points were imputed so they could be included in the analyses; and the specific method used AND the reasons for choosing the specific method were specified.

**References**

Anguera, M. T., Chacón-Moscoso, S., Holgado, F. P., & Pérez, J. A. (2008). Instrumentos en evaluación de programas [Instruments in program evaluation]. In M. T. Anguera, S. Chacón-Moscoso, & A. Blanco (Eds.), *Evaluación de programas sociales y sanitarios: un abordaje metodológico* (pp. 127–152). Síntesis.

Fergusson, D., Shawn, D. A., Gordon, G., & Hébert, P. (2002). Post-randomisation exclusions: The intention to treat principle and excluding patients from analysis. *BMJ*, *325*(7365), 652–654. https://doi.org/10.1136/bmj.325.7365.652

Fleischman, R. J., Adams, A. L., Hedges, J. R., Ma, O. J., Mullins, R. J., & Newgard, C. D. (2010). The optimum follow-up period for assessing mortality outcomes in injured older adults. *Journal of the American Geriatrics Society*, *58*(10), 1843–1849. https://doi.org/10.1111/j.1532-5415.2010.03065.x

Olivares, J., Rosa, A. I., & Sánchez-Meca, J. (2000). Meta-análisis de la eficacia de las habilidades de afrontamiento en problemas clínicos y de salud en España [Meta-analysis of the effectiveness of coping skills in clinical and health problems in Spain]. *Anuario de Psicología*, *31*, 43–61.

Sánchez-Meca, J. (1997). Methodological issues in the meta-evaluation of correctional treatment. In S. Redondo, V. Garrido, J. Pérez, & R. Barberet (Eds.), *Advances in Psychology and Law: International Contributions* (pp. 486–498). Walter de Gruyter.

Shadish, W. R., Cook, T. D., & Campbell, D. T. (2002). *Experimental and quasi-experimental designs for generalized causal inference*. Houghton Mifflin.
