# Supplementary material for: The Methodological Quality Scale (MQS) for intervention programs: validity evidence
Source: Front Psychol. 2023 Jul 6;14:1217661. doi: 10.3389/fpsyg.2023.1217661 (PMC10358327; doi:10.3389/fpsyg.2023.1217661)
Supplement: Supplementary file 3 [file Data_Sheet_3.DOCX]

Supplementary Material

The Methodological Quality Scale (MQS) for Intervention Programs: Validity Evidence

**Salvador Chacón-Moscoso*, Susana Sanduvete-Chaves, José Antonio Lozano-Lozano, Francisco Pablo Holgado-Tello**

*** Correspondence:** Salvador Chacón Moscoso: schacon@us.es

# Supplementary Data S3

**References of the 299 studies coded:**

Acharya, B., Tenpa, J., Basnet, M., Hirachan, S., Rimal, P., Choudhury, N., Thapa, P., Citrin, D., Halliday, S., Swar, S. B., van Dyke, C., Gauchan, B., Sharma, B., Hung, E., & Ekstrand, M. (2017). Developing a scalable training model in global mental health: Pilot study of a video-assisted training program for generalist clinicians in rural Nepal. *Global Mental Health, 4*, e8. <https://doi.org/10.1017/gmh.2017.4>

Aguirre-Muñoz, Z., Park, J.-E., Amabisca, A., & Boscardin, C. K. (2009). Developing teacher capacity for serving ELLs’ writing instructional needs: A case for systemic functional linguistics. *Bilingual Research Journal*, *31*(1), 295-322. <https://doi.org/10.1080/15235880802640755>

Alford, D. P., Bridden, C., Jackson, A. H., Saitz, R., Amodeo, M., Barnes, H. N., & Samet, J. H. (2009). Promoting substance use education among generalist physicians: An evaluation of the Chief Resident Immersion Training (CRIT) program. *Journal of General Internal Medicine*, *24*(1), 40–47. <https://doi.org/10.1007/s11606-008-0819-2>

Allan, C. K., Thiagarajan, R. R., Beke, D., Imprescia, A., Kappus, L. J., Garden, A., Hayes, G., Laussen, P. C., Bacha, E., & Weinstock, P. H. (2010). Simulation-based training delivered directly to the pediatric cardiac intensive care unit engenders preparedness, comfort, and decreased anxiety among multidisciplinary resuscitation teams. *Journal of Thoracic and Cardiovascular Surgery*, *140*(3), 646–652. <http://doi.org/10.1016/j.jtcvs.2010.04.027>

Allen, J., & Cohen, D. (2010). Attitudes to work and health in doctors in training. *Occupational Medicine*, *60*(8), 640–644. <http://doi.org/10.1093/occmed/kqq147>

Almog, D. M., Tsimidis, K., Moss, M. E., Gottlieb, R. H., & Carter, L. C. (2000). Evaluation of a training program for detection of carotid artery calcifications on panoramic radiographs. *Oral Surgery, Oral Medicine, Oral Pathology, Oral Radiology, and Endodontics, 90*(1), 111–117. <https://doi.org/10.1067/moe.2000.107056>

Alshomrani, A. T., & AlHadi, A. N. (2017). Learning environment of the Saudi psychiatry board training program. *Saudi Medical Journal, 38*(6), 629–635. <https://doi.org/10.15537/smj.2017.6.18164>

Anderson, C. (2006). Training efforts to reduce reports of workplace violence in a community health care facility. *Journal of Professional Nursing, 22*(5), 289–295. <https://doi.org/10.1016/j.profnurs.2006.07.007>

Armstrong, G., Kermode, M., Raja, S., Suja, S., Chandra, P., & Jorm, A. F. (2011). A mental health training program for community health workers in India: Impact on knowledge and attitudes. *International Journal of Mental Health Systems, 5*(1), 17. <https://doi.org/10.1186/1752-4458-5-17>

Arnetz, B. B. (1996). Techno-stress: A prospective psychophysiological study of the impact of a controlled stress-reduction program in advanced telecommunications systems design work. *Journal of Occupational & Environmental Medicine, 38*(1), 53–65. <https://doi.org/10.1097/00043764-199601000-00017>

Arranz, P., Ulla, S. M., Ramos, J. L., Del Rincón, C., & López-Fando, T. (2005). Evaluation of a counseling training program for nursing staff. *Patient Education and Counseling, 56*(2), 233–239. <https://doi.org/10.1016/j.pec.2004.02.017>

Arslan, R., Kuş, A., Mumcu, H., & Uzaslan, N. T. (2008). A model of cooperative education-“group leader training program” for industry employees. *Turkish Online Journal of Educational Technology*, *7*(4), 29–35. Retrieved from <https://www.scopus.com/inward/record.uri?eid=2-s2.0-77956235432&partnerID=40&md5=81aa9fc793a100e61a4fbb4901831cf9>

Ashurst, E. J., Jones, R. B., Williamson, G. R., Emmens, T., & Perry, J. (2012). Collaborative learning about e-health for mental health professionals and service users in a structured anonymous online short course: Pilot study. *BMC Medical Education*, *12*(1), 37. <https://doi.org/10.1186/1472-6920-12-37>

Atkinson, L. Z., Forrest, A., Marriner, L., Geddes, J., & Cipriani, A. (2017). Implementing tools to support evidence-based practice: A survey and brief intervention study of the National Elf Service across Oxford Health NHS Foundation Trust. *Evidence Based Mental Health*, *20*(2), 41-45. <https://doi.org/10.1136/eb-2017-102665>

Baer, J. S., Rosengren, D. B., Dunn, C. W., Wells, E. A., Ogle, R. L., & Hartzler, B. (2004). An evaluation of workshop training in motivational interviewing for addiction and mental health clinicians. *Drug and alcohol dependence, 73*(1), 99–106. <https://doi.org/10.1016/j.drugalcdep.2003.10.001>

Baker-Ericzén, M. J., Jenkins, M. M., Park, S., & Garland, A. F. (2015). Clinical decision-making in community children’s mental health: Using innovative methods to compare clinicians with and without training in evidence-based treatment. *Child & Youth Care Forum*, *44*(1), 133-157. <https://doi.org/10.1007/s10566-014-9274-x>

Baker-Henningham, H., & Walker, S. (2009). A qualitative study of teacher’s perceptions of an intervention to prevent conduct problems in Jamaican pre-schools. *Child: Care, Health and Development*, *35*(5), 632–642. <http://doi.org/10.1111/j.1365-2214.2009.00996.x>

Baker-Henningham, H., Scott, Y., Bowers, M., & Francis, T. (2019). Evaluation of a violence-prevention programme with jamaican primary school teachers: A cluster randomised trial. *International Journal of Environmental Research and Public Health, 16*(15): 2797. <https://doi.org/10.3390/ijerph16152797>

Banilower, E. R., Fulp, S. L., & Warren, C. L. (2010). *Science: It’s elementary. Year four evaluation report*. Horizon Research, 326 Cloister Court, Chapel Hill, NC 27514. Retrieved from <https://search.proquest.com/docview/870288618?accountid=14744>

Bannon, E. (2010). The effectiveness of an acceptance and commitment therapy intervention for work stress on innovation (Publication No. bgsu1288749862) [Doctoral dissertation, Bowling Green State University]. OhioLINK.

Barak, M., & Shlomo, W. (1997) An Israeli study of longitudinal in-service training of mathematics, science and technology teachers. *Journal of Education for Teaching, 23*(2), 179-190. <http://doi.org/10.1080/02607479720114>

Baynes, J., & Gregorio, N. (2008). Nursery training for smallholders: An evaluation of two extension programs in the Philippines. *Small-Scale Forestry, 7*(3–4), 387–401. <https://doi.org/10.1007/s11842-008-9061-3>

Beaumont, E., Irons, C., Rayner, G., & Dagnall, N. (2016). Does compassion-focused therapy training for health care educators and providers increase self-compassion and reduce self-persecution and self-criticism? *Journal of Continuing Education in the Health Professions*, *36*(1), 4–10. <https://doi.org/10.1097/CEH.0000000000000023>

Beidas, R. S., Edmunds, J. M., Marcus, S. C., & Kendall, P. C. (2012). Training and consultation to promote implementation of an empirically supported treatment: A randomized trial. *Psychiatric Services*, *63*(7), 660-665. <https://doi.org/10.1176/appi.ps.201100401>

Bell, A., & Morris, G. (2009). Engaging Professional Learning in Online Environments. *Australasian Journal of Educational Technology*, *25*(5), 700–713. <https://doi.org/10.14742/ajet.1116>

Bell, J. L., & Grushecky, S. T. (2006). Evaluating the effectiveness of a logger safety training program*. Journal of Safety Research, 37*(1), 53–61. <https://doi.org/10.1016/j.jsr.2005.10.019>

Bena, A., Berchialla, P., Coffano, E., Debernardi, M., Icardi, L., & Dettoni, L. (2009). Effectiveness of a training programme in reducing occupational injuries: The Turin-Novara high-speed rail- way line experience. *Medicina del Lavoro*, *100*(4), 295–298. Retrieved from: <https://pubmed.ncbi.nlm.nih.gov/19764188/>

Björkdahl, A., Hansebo, G., & Palmstierna, T. (2013). The influence of staff training on the violence prevention and management climate in psychiatric inpatient units: Violence prevention and management staff training. *Journal of Psychiatric and Mental Health Nursing*, *20*(5), 396-404. <https://doi.org/10.1111/j.1365-2850.2012.01930.x>

Black, L.-A., & Roberts, P. (2009). People with a learning disability as trainers: Evaluation of a values based pilot training programme. *British Journal of Learning Disabilities*, *37*(2), 129–137. <http://doi.org/10.1111/j.1468-3156.2008.00533.x>

Boehnlein, J. K., Leung, P. K., & Kinzie, J. D. (2008). Cross-Cultural psychiatric residency training: The Oregon experience. *Academic Psychiatry*, *32*(4), 299-305. <https://doi.org/10.1176/appi.ap.32.4.299>

Boone, B. N., King, M. L., Gresham, L. S., Wahl, P., & Suh, E. (2008). Conflict management training and nurse-physician collaborative behaviors. *Journal for Nurses in Staff Development*, *24*(4), 168–175. <http://doi.org/10.1097/01.NND.0000320670.56415.91>

Bracker, A. L., Morse, T. F., & Simcox, N. J. (2009). Training health and safety committees to use control banding: Lessons learned and opportunities for the united states. *Journal of Occupational and Environmental Hygiene*, *6*(5), 307–314. <http://doi.org/10.1080/15459620902810083>

Brady, S., O’Connor, N., Burgermeister, D., & Hanson, P. (2012). The impact of mindfulness meditation in promoting a culture of safety on an acute psychiatric unit. *Perspectives in Psychiatric Care*, *48*(3), 129-137. <https://doi.org/10.1111/j.1744-6163.2011.00315.x>

Bray, J., & Howard, G. S. (1980). Methodological considerations in the evaluation of a teacher-training program. *Journal of Educational Psychology - Propósitos y Representaciones, 72*(1), 62–70. <https://doi.org/10.1037/0022-0663.72.1.62>

Bredimus, B. K. (2020). Mental health preparedness: A nurse leader’s role. *Nurse Leader, 18*(1), 48-53. <https://doi.org/10.1016/j.mnl.2019.11.008>

Brightling, P. B., Dyson, R. D., Hope, A. F., & Penry, J. (2009). A national programme for mastitis control in Australia: Countdown Downunder*. Irish Veterinary Journal, 62*(4), 52-58. <https://doi.org/10.1186/2046-0481-62-S4-S52>

Brodsky, B. S., Cabaniss, D. L., Arbuckle, M., Oquendo, M. A., & Stanley, B. (2017). Teaching dialectical behavior therapy to psychiatry residents: The Columbia Psychiatry Residency DBT curriculum. *Academic psychiatry: The journal of the American Association of Directors of Psychiatric Residency Training and the Association for Academic Psychiatry, 41*(1), 10–15. <https://doi.org/10.1007/s40596-016-0593-0>

Brooker, C., Tarrier, N., Barrowclough, C., Butterworth, A., & Goldberg, D. (1992). Training community psychiatric nurses for psychosocial intervention. *The British Journal of Psychiatry, 160*(6), 836–844. <https://doi.org/10.1192/bjp.160.6.836>

Brooker, C., Tarrier, N., Barrowclough, C., Butterworth, A., & Goldberg, D. (1994). The outcome of training community psychiatric nurses to deliver psychosocial intervention. *British Journal of Psychiatry, 165*, 222–230. <https://doi.org/10.1192/bjp.165.2.222>

Brookman-Frazee, L. I., Drahota, A., & Stadnick, N. (2012). Training community mental health therapists to deliver a package of evidence-based practice strategies for school-age children with autism spectrum disorders: a pilot study*. Journal of Autism and Developmental Disorders, 42*(8), 1651–1661. <https://doi.org/10.1007/s10803-011-1406-7>

Brooks, H., Lovell, K., Bee, P., Fraser, C., Molloy, C., & Rogers, A. (2019). Implementing an intervention designed to enhance service user involvement in mental health care planning: A qualitative process evaluation. *Social Psychiatry and Psychiatric Epidemiology, 54*(2), 221-233. <https://doi.org/10.1007/s00127-018-1603-1>

Brown, K., McCloskey, C., Galpin, D., Keen, S., & Immins, T. (2008). Evaluating the impact of post-qualifying social work education. *Social Work Education*, *27*(8), 853–867. <https://doi.org/http://dx.doi.org/10.1080/02615470701844217>

Brown, R. F., Bylund, C. L., Gueguen, J. A., Diamond, C., Eddington, J., & Kissane, D. (2010). Developing patient-centered communication skills training for oncologists: describing the content and efficacy of training. *Communication Education*, *59*(3), 235–248. Retrieved from <https://search.proquest.com/docview/742877519?accountid=14744>

Browne, J., & Ponce, A. (2020). Assessing food insecurity in individuals with serious mental illness: A pilot training for community mental health providers. *Community Mental Health Journal*, *56*(6), 1110-1114. <https://doi.org/10.1007/s10597-020-00593-9>

Brownlow, R. S., Maguire, S., O’Dell, A., Dias-da-Costa, C., Touyz, S., & Russell, J. (2015). Evaluation of an online training program in eating disorders for health professionals in Australia. *Journal of Eating Disorders*, *3*(1), 37. <https://doi.org/10.1186/s40337-015-0078-7>

Bryson, S. A., & Ostmeyer, K. F. (2014). Increasing the effectiveness of community mental health center social skills groups for children with autism spectrum disorder: A training and consultation example. *Administration and Policy in Mental Health and Mental Health Services Research, 41*(6), 808-821. <https://doi.org/10.1007/s10488-013-0533-1>

Busch, C., Staar, H., Åborg, C., Roscher, S., & Ducki, A. (2010). The neglected employees: Work-life balance and a stress management intervention program for low-qualified workers. In J. Houdmont & S. Leka (Eds.), *Contemporary occupational health psychology: Global perspectives on research and practice, Vol. 1 BT - Contemporary occupational health psychology: Global perspectives on research and practice, Vol. 1* (p. 98–123). Wiley-Blackwell. <https://doi.org/10.1002/9780470661550.ch6>

Calderón, C., Balagué, L., Iruin, Á., Retolaza, A., Belaunzaran, J., Basterrechea, J., & Mosquera, I. (2016). Primary care and mental health care collaboration in patients with depression: Evaluation of a pilot experience. *Atencion Primaria*, *48*(6), 356–365. <http://doi.org/10.1016/j.aprim.2015.06.013>

Cameron, J., Lee, N. K., & Harney, A. (2010). Changes in attitude to, and confidence in, working with comorbidity after training in screening and brief intervention. *Mental Health and Substance Use: Dual Diagnosis*, *3*(2), 124–130. <http://doi.org/10.1080/17523281003712674>

Cardoso, G., Papoila, A., Tomé, G., Killaspy, H., King, M., & Caldas-de-Almeida, J. M. (2017). A cluster randomised controlled trial of a staff-training intervention in residential units for people with long-term mental illness in Portugal: The PromQual trial. *Social Psychiatry and Psychiatric Epidemiology*, *52*(11), 1435-1445. <https://doi.org/10.1007/s00127-017-1416-7>

Carmel, A., Logvinenko, E., & Valenti, E. S. (2019). Evaluation of a dialectical behavior therapy psychiatry residency training program. *Academic Psychiatry*, *43*(1), 37-40. <https://doi.org/10.1007/s40596-018-0993-4>

Carpenter, J., Milne, D., Lombardo, C., & Dickinson, C. (2007). Process and outcomes of training in psychosocial interventions in mental health: A stepwise approach to evaluation. *Journal of Mental Health*, *16*(4), 505-520. <https://doi.org/10.1080/09638230701482329>

Chandy, H., Steinholt, M., & Husum, H. (2007). Delivery life support: A preliminary report on the chain of survival for complicated deliveries in rural Cambodia. *Nursing and Health Sciences, 9*(4), 263-269. <https://doi.org/10.1111/j.1442-2018.2007.00321.x>

Chapman, L., & Blackman, J. (2010). Using supported learning to ensure nurse recruits are skilled to care for acutely ill patients. *Nursing Times*, *106*(11), 10–11. Retrieved from <https://www.scopus.com/inward/record.uri?eid=2-s2.0-77951627582&partnerID=40&md5=c885c6a4ca3b87f175a5c98cb7a52cca>

Chau, P. Y. K., & Hu, P. J. (2002). Examining a model of information technology acceptance by individual professionals: An exploratory study. *Journal of Management Information Systems, 18*(4), 191–229. <https://doi.org/10.1080/07421222.2002.11045699>

Cheron, D. M., Chiu, A., Stanick, C. F., Stern, H. G., Donaldson, A. R., Daleiden, E. L., & Chorpita, B. F. (2019). Implementing evidence based practices for children's mental health: A case study in implementing modular treatments in community mental health. *Administration and Policy in Mental Health, 46*(3), 391–410. <https://doi.org/10.1007/s10488-019-00922-5>

Christie, G., Black, S., Dunbar, L., Pulford, J., & Wheeler, A. (2013). Attitudes, skills and knowledge change in child and adolescent mental health workers following AOD screening and brief intervention training*. International Journal of Mental Health and Addiction, 11*(2), 232-246. <https://doi.org/10.1007/s11469-012-9414-1>

Clark, P. G. (2002). Evaluating an interdisciplinary team training institute in geriatrics: Implications for teaching teamwork theory and practice. *Educational Gerontology, 28*(6), 511-528,<https://doi.org/10.1080/03601270290081425>

Clark, P. G., Leinhaas, M. M., & Filinson, R. (2002). Developing and evaluating an interdisciplinary clinical team training program: Lessons taught and lessons learned. *Educational Gerontology, 28*(6), 491–510. <https://doi.org/10.1080/03601270290081416>

Colarossi, L., Billowitz, M., & Breitbart, V. (2010). Emergency contraception education for health and human service professionals: An evaluation of knowledge and attitudes. *Health Education Journal*, *69*(2), 175–182. <http://doi.org/10.1177/0017896910364886>

Coleman, M. T., Roberts, K., Wulff, D., Van Zyl, R., & Newton, K. (2008). Interprofessional ambulatory primary care practice-based educational program. *Journal of Interprofessional Care, 22*(1), 69–84. <http://doi.org/10.1080/13561820701714763>

Collins, M. E., Hill, N., & Miranda, C. (2008). Establishing positive youth development approaches in group home settings: Training implementation and evaluation. *Child and Adolescent Social Work Journal*, *25*(1), 43–54. <http://doi.org/10.1007/s10560-008-0111-7>

Collins, P. Y., Mestry, K., Wainberg, M. L., Nzama, T., & Lindegger, G. (2006). Training South African mental health care providers to talk about sex in the era of AIDS. *Psychiatric Services, 57*(11), 1644-1647. <https://doi.org/10.1176/ps.2006.57.11.1644>

Corcoran, T. B. (2008). *The Inquiry Based Science and Technology Education Program (IN-STEP): The evaluation of the first year*. Consortium for Policy Research in Education. University of Pennsylvania, Philadelphia. Retrieved from <https://search.proquest.com/docview/61832922?accountid=14744>

Cotter, J. J., Coogle, C. L., Parham, I. A., Head, C., Fulton, L. Q., Watson, K., & Curtis, A. (2004). Designing a multi-disciplinary geriatrics health professional mentoring program. *Educational Gerontology, 30*(2), 107-117. <https://doi.org/10.1080/03601270490266275>

Coyne, P., Paice, J. A., Ferrell, B. R., Malloy, P., Virani, R., & Fennimore, L. A. (2007). Oncology end-of-life nursing education consortium training program: Improving palliative care in cancer. *Oncology Nursing Forum, 34*(4), 801-807. <https://doi.org/10.1188/07.ONF.801-807>

De Beurs, D. P., Bosmans, J. E., deGroot, M. H., de Keijser, J., van Duijn, E., de Winter, R. F. P. & Kerkhof, A. J. F. M. (2015a). Training mental health professionals in suicide practice guideline adherence: Cost-effectiveness analysis alongside a randomized controlled trial. *Journal of Affective Disorders, 186,* 203-210. <http://doi.org/10.1016/j.jad.2015.07.028>

de Beurs, D. P., de Groot, M. H., de Keijser, J., Mokkenstorm, J., van Duijn, E., de Winter, R. F. P., & Kerkhof, A. J. F. M. (2015b). The effect of an e-learning supported Train-the-Trainer programme on implementation of suicide guidelines in mental health care. *Journal of Affective Disorders*, *175*, 446-453. <https://doi.org/10.1016/j.jad.2015.01.046>

Deane, F. P., Goff, R. O., Pullman, J., Sommer, J., & Lim, P. (2019). Changes in mental health providers’ recovery attitudes and strengths model implementation following training and supervision. *International Journal of Mental Health and Addiction, 17*(6), 1417-1431. <https://doi.org/10.1007/s11469-018-9885-9>

De Oliveira Andrade, P. M., De Oliveira Ferreira, F., & Haase, V. G. (2011). Multidisciplinary perspective for cerebral palsy assessment after an international, classification of functioning, disability and health training. *Developmental Neurorehabilitation*, *14*(4), 199–207. <http://doi.org/10.3109/17518423.2011.584781>

Dilley, J. A., Reuer, J. R., Colman, V., & Norman, R. K. (2009). Steps to a Healthier Washington. *Health Promotion Practice, 10(*2), 138-145. <https://doi.org/10.1177/1524839909332601>

Dimeff, L. A., Harned, M. S., Woodcock, E. A., Skutch, J. M., Koerner, K., & Linehan, M. M. (2015). Investigating bang for your training buck: A randomized controlled trial comparing three methods of training clinicians in two core strategies of dialectical behavior therapy. *Behavior Therapy, 46*(3), 283-295. <https://doi.org/10.1016/j.beth.2015.01.001>

Dimeff, L. A., Koerner, K., Woodcock, E. A., Beadnell, B., Brown, M. Z., Skutch, J. M., Paves, A. P., Bazinet, A., & Harned, M. S. (2009). Which training method works best? A randomized controlled trial comparing three methods of training clinicians in dialectical behavior therapy skills. *Behaviour Research and Therapy*, *47*(11), 921-930. <https://doi.org/10.1016/j.brat.2009.07.011>

Ditton-Phare, P., Sandhu, H., Kelly, B., Kissane, D., & Loughland, C. (2016). Pilot evaluation of a communication skills training program for psychiatry residents using standardized patient assessment. *Academic Psychiatry*, *40*(5), 768–775. <https://doi.org/10.1007/s40596-016-0560-9>

Dobie, A., Tucker, A., Ferrari, M., & Rogers, J. M. (2016). Preliminary evaluation of a brief mindfulness-based stress reduction intervention for mental health professionals. *Australasian Psychiatry*, *24*(1), 42-45. <https://doi.org/10.1177/1039856215618524>

Dondanville, K. A., Fina, B. A., Straud, C. L., Finley, E. P., Tyler, H., Jacoby, V., Blount, T. H., Moring, J. C., Pruiksma, K. E., Blankenship, A. E., Evans, W. R., & Zaturenskaya, M. (2020). Launching a competency-based training program in evidence-based treatments for PTSD: Supporting veteran-serving mental health providers in Texas. *Community Mental Health Journal*. <https://doi.org/10.1007/s10597-020-00676-7>

Dörries, A., Simon, A., Neitzke, G., & Vollmann, J. (2010). Implementing clinical ethics in German hospitals: Content, didactics and evaluation of a nationwide postgraduate training programme. *Journal of Medical Ethics*, *36*(12), 721–726. <http://doi.org/10.1136/jme.2010.036137>

Drahota, A., Stadnick, N., & Brookman-Frazee, L. (2014). Therapist perspectives on training in a package of evidence-based practice strategies for children with autism spectrum disorders served in community mental health clinics. *Administration and Policy in Mental Health and Mental Health Services Research, 41*(1), 114-125. <https://doi.org/10.1007/s10488-012-0441-9>

Dugmore, P., & Cocker, C. (2008). Legal, social and attitudinal changes: An exploration of lesbian and gay issues in a training programme for social workers in fostering and adoption. *Social Work Education*, *27*(2), 159–168. <http://doi.org/10.1080/02615470701709600>

Durand, M., Labarère, J., Brunet, E., & Pons, J.-C. (2003). Evaluation of a training program for healthcare professionals about breast-feeding*. European Journal of Obstetrics, Gynecology, and Reproductive Biology, 106*(2), 134–8. <http://doi.org/10.1016/s0301-2115(02)00225-7>.

Duthie, G. S., Drew, P. J., Hughes, M. A. P., Farouk, R., Hodson, R., Wedgwood, K. R., & Monson, J. R. T. (1998). A UK training programme for nurse practitioner flexible sigmoidoscopy and a prospective evaluation of the practice of the first UK trained nurse flexible sigmoidoscopist. *Gut., 43*(5), 711-714. <https://doi.org/10.1136/gut.43.5.711>

Dyck, K. G., Cornock, B. L., Gibson, G., & Carlson, A. A. (2008). Training clinical psychologists for rural and northern practice: Transforming challenge into opportunity. *Australian Psychologist, 43*(4), 239-248. <https://doi.org/10.1080/00050060802438096>

Egener, B. (2008). Addressing physicians’ impaired communication skills. *Journal of General Internal Medicine*, *23*(11), 1890–1895. <https://doi.org/http://dx.doi.org/10.1007/s11606-008-0778-7>

Eriksson, T., Germundsjö, L., Åström, E., & Rönnlund, M. (2018). Mindful self-compassion training reduces stress and burnout symptoms among practicing psychologists: A Randomized controlled trial of a brief web-based intervention. *Frontiers in Psychology, 9*: 2340. <https://doi.org/10.3389/fpsyg.2018.02340>

Eson-Brizo, J. (2010). Analysis of a mentoring program to change attitudes related to turnover of special needs teachers (Publication No. ED508645) [Doctoral dissertation, Nova Southeastern University]. ERIC.

Espinoza, G. R., Danilla, E. S., Valdes, G. F., San Francisco, R. I., & Llanos, L. O. (2009). Resident evaluation of general surgery training programs. *Revista Médica de Chile*, *137*(7), 940–945. <http://doi.org/10.4067/S0034-98872009000700013>

Fadden, G. (1997). Implementation of family interventions in routine clinical practice following staff training programs: A major cause for concern. *Journal of Mental Health*, *6*(6), 599-612. <https://doi.org/10.1080/09638239718464>

Fairburn, C. G., Allen, E., Bailey-Straebler, S., O’Connor, M. E., & Cooper, Z. (2017). Scaling up psychological treatments: A countrywide test of the online training of therapists. *Journal of Medical Internet Research, 19(*6), e214. <https://doi.org/10.2196/jmir.7864>

Fatima, J., & Naseer Ud Din, M. (2010). Evaluative study of M.A. education programmes of teacher education at higher education level in Pakistan. *Contemporary Issues in Education Research*, *3*(12), 29–34. <http://doi.org/10.19030/cier.v3i12.921>

Fernandez, G. L., Lee, P. C., Page, D. W., D’Amour, E. M., Wait, R. B., & Seymour, N. E. (2010). Implementation of full patient simulation training in surgical residency. *Journal of Surgical Education*, *67*(6), 393–399. <http://doi.org/10.1016/j.jsurg.2010.07.005>

Fields, R. C., Bowman, M. C., Freeman, B. D., & Klingensmith, M. E. (2009). Implementation of an “after hours” resident educational program in a general surgery residency: A paradigm for increasing formal didactic training outside of the hospital setting in the era of the 80-hour workweek. *Journal of Surgical Education*, *66*(6), 340–343. <http://doi.org/10.1016/j.jsurg.2009.09.008>

Fitzgerald, M. A., Chromy, B., Philbrick, C. A., Sanders, G. F., Muske, K. L., & Bratteli, M. (2009). The North Dakota mental health and aging education project: Curriculum design and training outcomes for a train-the-trainer model. *Gerontology & Geriatrics Education*, *30*(2), 114–129. <http://doi.org/10.1080/02701960902911257>

Fletcher, S. (2009). A critical examination of fidelity, adaptability, and maintenance in a national training program for staff in long-term care [Doctoral dissertation, University of North Carolina]*.* Carolina Digital Repository <https://doi.org/10.17615/z4r8-ze07>

Fogarty, C. T., Winters, P., & Farah, S. (2016). Improving patient-centered communication while using an electronic health record: Report from a curriculum evaluation. *International Journal of Psychiatry in Medicine*, *51*(4), 379–389. <http://doi.org/10.1177/0091217416659274>

Forsyth, E., Joseph, M., & Perry, L. (2009). Learning about Learning 2.0: Evaluating the “New South Wales Public Library Learning 2.0” Program. *Australian Academic & Research Libraries*, *40*(3), 172–191. <http://doi.org/10.1080/00048623.2009.10721403>

Franke, T., Bagdasaryan, S., & Furman, W. (2009). A multivariate analysis of training, education, and readiness for public child welfare practice. *Children and Youth Services Review*, *31*(12), 1330–1336. <http://doi.org/10.1016/j.childyouth.2009.06.004>

Fraser, C., Grundy, A., Meade, O., Callaghan, P., & Lovell, K. (2017). EQUIP training the trainers: An evaluation of a training programme for service users and carers involved in training mental health professionals in user-involved care planning. *Journal of Psychiatric and Mental Health Nursing, 24(*6), 367-376. <https://doi.org/10.1111/jpm.12361>

Fritzsche, K., Scheib, P., Ko, N., Wirsching, M., Kuhnert, A., Hick, J., Schüßler, G., Wu, W., Yuan, S., Cat, N. H., Vongphrachanh, S., Linh, N. T., Viet, N. K., & and the ASIA-LINK Workgroup. (2012). Results of a psychosomatic training program in China, Vietnam and Laos: Successful cross-cultural transfer of a postgraduate training program for medical doctors. *BioPsychoSocial Medicine*, *6*(1), 17. <https://doi.org/10.1186/1751-0759-6-17>

Gabor, K., Tulkán, I., Helembai, K., Csanádi, J., Szogi, S., & Kntor, K. (2008). At the European union’s door: A Hungarian team’s experiences of participating in the Project Leonardo for better nursing care. *International Journal of Nursing Practice, 14*(4), 329-334. <https://doi.org/10.1111/j.1440-172X.2008.00704.x>

Gage, M. A., Fredericks, D. H., Johnson-Dorn, N., & Lindley-Southard, B. (2009). In-service training for staffs of group homes and work activity centers serving developmentally disabled adults. *Research and Practice for Persons with Severe Disabilities (RPSD)*, *34*(2), 49–58. <https://doi.org/10.1016/j.ridd.2013.11.009>

Gagnon, M. (2005). Ergonomic identification and biomechanical evaluation of workers’ strategies and their validation in a training situation: *Summary of research. Clinical Biomechanics, 20*(6), 569–580. <https://doi.org/10.1016/j.clinbiomech.2005.03.007>

García, P. J., Cotrina, A., Gotuzzo, E., Gonzalez, E., & Buffardi, A. L. (2010). Research training needs in Peruvian national TB/HIV programs. *BMC Medical Education*, *10*(1): 63. <https://doi.org/10.1186/1472-6920-10-63>

Gentry, W. D. (1974). Technicians' views of training and function. *Professional Psychology, 5*(2), 219–221. https://doi.org/10.1037/h0037555

Geoffrion, S., Goncalves, J., Giguère, C.-É., & Guay, S. (2018). Impact of a program for the management of aggressive behaviors on seclusion and restraint use in two high-risk units of a mental health institute*. Psychiatric Quarterly, 89*(1), 95-102. <https://doi.org/10.1007/s11126-017-9519-6>

Gifford, V., Niles, B., Rivkin, I., Koverola, C., & Polaha, J. (2012). Continuing education training focused on the development of behavioral telehealth competencies in behavioral healthcare providers. *Rural and Remote Health*, *12*(4). Retrived from www.rrh.org.au/journal/article/2108

Gliksman, L., McKenzie, D., Single, E., Douglas, D., Brunet, S., & Moffatt, K. (1993). The role of alcohol providers in prevention: An evaluation of a server intervention programme. *Addiction*, *88*(9), 1195-203. <https://doi.org/10.1111/j.1360-0443.1993.tb02142.x>

Gopalan, P., Glance, J., Valpey, R., Joseph, H., & Shenai, N. (2018). Development of a women’s mental health curriculum and evolution to a women’s mental health area of concentration in a psychiatry residency program. *Archives of Women’s Mental Health, 21*(1), 113-116. <https://doi.org/10.1007/s00737-017-0788-8>

Goss, D. L., Christopher, G. E., Faulk, R. T., & Moore, J. (2009). Functional training program bridges rehabilitation and return to duty. *Journal of Special Operations Medicine : A Peer Reviewed Journal for SOF Medical Professionals*, *9*(2), 29–48. Retrieved from https://pubmed.ncbi.nlm.nih.gov/19813517/

Gould, M., Greenberg, N., & Hetherton, J. (2007). Stigma and the military: Evaluation of a PTSD psychoeducational program. *Journal of Traumatic Stress, 20*(4), 505–515. <https://doi.org/10.1002/jts>.

Grabovac, A., Clark, N., & McKenna, M. (2008). Pilot study and evaluation of postgraduate course on “the interface between spirituality, religion and psychiatry.” *Academic Psychiatry*, *32*(4), 332–337. <https://doi.org/10.1176/appi.ap.32.4.332>

Graham, A. L., Julian, J., & Meadows, G. (2010). Improving responses to depression and related disorders: Evaluation of an innovative, general, mental health care workers training program. *International Journal of Mental Health Systems*, *4*(1), 25. <https://doi.org/10.1186/1752-4458-4-25>

Grainger, M. N., Hegarty, S., Schofield, P., White, V., & Jefford, M. (2010). Discussing the transition to palliative care: Evaluation of a brief communication skills training program for oncology clinicians. *Palliative and Supportive Care*, *8*(4), 441–447. <http://doi.org/10.1017/S1478951510000313>

Graydon, M. M., Corno, C. M., Schacht, R. L., Knoblach, D. J., Wiprovnick, A. E., Thrash, S. T., Petersen, A. A., & DiClemente, C. C. (2018). A statewide initiative to train behavioral health providers in smoking cessation. *Translational Behavioral Medicine, 8*(6), 855-866. <https://doi.org/10.1093/tbm/iby086>

Grundy, A. C., Walker, L., Meade, O., Fraser, C., Cree, L., Bee, P., Lovell, K., & Callaghan, P. (2017). Evaluation of a co-delivered training package for community mental health professionals on service user- and carer-involved care planning. *Journal of Psychiatric and Mental Health Nursing*, *24*(6), 358-366. <https://doi.org/10.1111/jpm.12378>

Guay, S., Goncalves, J., & Boyer, R. (2016). Evaluation of an education and training program to prevent and manage patients’ violence in a mental health setting: A pretest-posttest intervention study. *Healthcare, 4(*3): 49. <https://doi.org/10.3390/healthcare4030049>

Hahn, S., Needham, I., Abderhalden, C., Duxbury, J. A. D., & Halfens, R. J. G. (2006). The effect of a training course on mental health nurses’ attitudes on the reasons of patient aggression and its management. *Journal of Psychiatric and Mental Health Nursing*, *13*(2), 197-204. <https://doi.org/10.1111/j.1365-2850.2006.00941.x>

Haller, G., Garnerin, P., Morales, M.-A., Pfister, R., Berner, M., Irion, O., Clergue, F., & Kern, C. (2008). Effect of crew resource management training in a multidisciplinary obstetrical setting. *International Journal for Quality in Health Care*, *20*(4), 254–263. <http://doi.org/10.1093/intqhc/mzn018>

Hallman, I. S., O’Connor, N., Hasenau, S., & Brady, S. (2014). Improving the culture of safety on a high-acuity inpatient child/adolescent psychiatric unit by mindfulness-based stress reduction training of staff: Improving the culture of safety. *Journal of Child and Adolescent Psychiatric Nursing, 27*(4), 183-189. <https://doi.org/10.1111/jcap.12091>

Hardin, B. J., Lower, J. K., Smallwood, G. R., Chakravarthi, S., Li, L., & Jordan, C. (2010). Teachers, families, and communities supporting english language learners in inclusive pre-kindergartens: An Evaluation of a professional development model. *Journal of Early Childhood Teacher Education*, *31*(1), 20–36. <https://doi.org/10.1080/10901020903539580>

Hardman, F., Abd-Kadir, J., Agg, C., Migwi, J., Ndambuku, J., & Smith, F. (2009). Changing pedagogical practice in Kenyan primary schools: The impact of school-based training. *Comparative Education*, *45*(1), 65–86. <https://doi.org/10.1080/03050060802661402>

Harrington, D., Materna, B., Vannoy, J., & Scholz, P. (2009). Conducting effective tailgate trainings. *Health Promotion Practice*, *10*(3), 359–69. <https://doi.org/10.1177/1524839907307885>

Harris, J. I., Leskela, J., Lakhan, S., Usset, T., DeVries, M., Mittal, D., & Boyd, J. (2019). Developing organizational interventions to address stigma among mental health providers: A pilot study. *Community Mental Health Journal*, *55*(6), 924-931. <https://doi.org/10.1007/s10597-019-00393-w>

Hawley, J., & Barnard, J. (2005). Work environment characteristics and implications for training transfer: A case study of the nuclear power industry. *Human Resource Development International, 8*(1), 65-80. <https://doi.org/10.1080/1367886042000338308>

Head, B. A., Lajoie, S., Augustine-Smith, L., Cantrell, M., Hofmann, D., Keeney, C., & Pfeifer, M. (2010). Palliative care case management: Increasing access to community-based palliative care for medicaid recipients. *Professional Case Management*, *15*(4), 206–217. <http://doi.org/10.1097/NCM.0b013e3181d18a9e>

Heinrichs, W. L., Youngblood, P., Harter, P. M., & Dev, P. (2008). Simulation for team training and assessment: Case studies of online training with virtual worlds. *World Journal of Surgery*, *32*(2), 161–170. <http://doi.org/10.1007/s00268-007-9354-2>

Herman, R., Kaplan, M., Satriano, J., Cournos, F., & McKinnon, K. (1994). HIV prevention with persons with serious mental illness: Staff training and institutional attitudes. *Psychosocial Rehabilitation Journal, 17*, 97–104. http://doi.org/[10.1037/h0095553](https://www.researchgate.net/deref/http%3A%2F%2Fdx.doi.org%2F10.1037%2Fh0095553?_sg%5B0%5D=prYY7s_te3Jr6-jOmkqKBqCi-zdkNHROYJd3p2dIZgVXayraT3X2u3RcL7XCvI9ZBqtMcPdb3jjae-XqGynB1MaIqQ.fYNITJFHKDiVDXH_i5rN_IOX4o6gk7Zq9QKCgfOrvMAKfEw8ZbyXP1I_Ny8jWgVoxhiqbiU_Bm2SF4ftnRSWqQ)

Hinchey, K. T., Iwata, I., Picchioni, M., & McArdle, P. J. (2009). “I can do patient care on my own”: Autonomy and the manager role. *Academic Medicine*, *84*(11), 1516–1521. <http://doi.org/10.1097/ACM.0b013e3181bb208c>

Hollenbeck, K., & Timmeney, B. (2009). *Lessons learned from a state-funded workplace literacy program. Upjohn Institute Staff Working Paper No. 09-146*. W. E. Upjohn Institute for Employment Research. Retrieved from <https://search.proquest.com/docview/742874792?accountid=14744>

Houston, T. K., Richman, J. S., Ray, M. N., Allison, J. J., Gilbert, G. H., Shewchuk, R. M., Kohler, C. L., Kiefe, C. I., & DPBRN Collaborative Group. (2008). Internet delivered support for tobacco control in dental practice: Randomized controlled trial. *Journal of Medical Internet Research*, *10*(5), e38. <https://doi.org/10.2196/jmir.1095>

Huang, C. C., Blake, A., Edwards, R. L., Liu, C. W., Nolan, R. B., Rusen, B., & Thompson, D. (2010). Professional knowledge of child support staff: Evidence from the New Jersey child support training program. *Evaluation Review*, *34*(1), 3–18. <http://doi.org/10.1177/0193841X09353302>

Hunter, S. B., Watkins, K. E., Wenzel, S., Gilmore, J., Sheehe, J., & Griffin, B. (2005). Training substance abuse treatment staff to care for co-occurring disorders. *Journal of Substance Abuse Treatment*, *28*(3), 239-245. <https://doi.org/10.1016/j.jsat.2005.01.009>

Iliffe, S., Eden, A., Downs, M., & Rae, C. (1999). The diagnosis and management of dementia in primary care: Development, implementation and evaluation of a national training programme. *Aging and Mental Health, 3*(2), 129–135. <https://doi.org/10.1080/13607869956280>

Irvine, A. B., Ary, D. V., & Bourgeois, M. S. (2003). An interactive multimedia program to train professional caregivers. *Journal of Applied Gerontology, 22*(2), 269–288. <https://doi.org/10.1177/0733464803022002006>

Johnson, H. A., & Roman, M. (2003). Geriatric continuing education in the workplace: Utilizing interactive television (ITV) technology in theory and in practice. *Educational Gerontology, 29*(7), 597–616. <https://doi.org/10.1080/713844416>

Jones, J. (2010). Leadership lessons from the fast track programme for teachers in England. *Educational Management Administration & Leadership*, *38*(2), 149–163. <https://doi.org/10.1177/1741143209356358>

Jonikas, J. A., Cook, J. A., Rosen, C., Laris, A., & Kim, J.-B. (2004). Brief reports: A program to reduce use of physical restraint in psychiatric inpatient facilities. *Psychiatric Services*, *55*(7), 818-820. <https://doi.org/10.1176/appi.ps.55.7.818>

Judkins, S., Reid, B., & Furlow, L. (2006). Hardiness training among nurse managers: Building a healthy workplace. *Journal of Continuing Education in Nursing, 37*(5), 202-207. <https://doi.org/10.3928/00220124-20060901-03>

Jullien, H., Bisch, C., Largouët, N., Manouvrier, C., Carling, C. J., & Amiard, V. (2008). Does a short period of lower limb strength training improve performance in field-based tests of running and agility in young professional soccer players? *Journal of Strength and Conditioning Research*, *22*(2), 404–11. <https://doi.org/10.1519/JSC.0b013e31816601e5>

Kaiser, T. L., & Kuechler, C. F. (2008). Training supervisors of practitioners: Analysis of efficacy. *Clinical Supervisor, 27*(1), 76–96. <https://doi.org/10.1080/07325220802221538>

Kalev, A. (2009). Cracking the glass cages? Restructuring and ascriptive inequality at work. *American Journal of Sociology*, *114*(6), 1591–1643. <https://doi.org/10.1086/597175>

Kalkat, R. K., & Khan, K. S. (2010). Meeting advanced learning needs of senior postgraduate trainees through practice-based reflective medical education: Evaluation of a formal structured training programme in obstetrics and gynaecology. *Journal of Obstetrics and Gynaecology*, *30*(2), 115–118. <http://doi.org/10.3109/01443610903477564>

Kamiru, H. N., Ross, M. W., Bartholomew, L. K., McCurdy, S. A., & Kline, M. W. (2009). Effectiveness of a training program to increase the capacity of health care providers to provide HIV/AIDS care and treatment in Swaziland. *Aids Care,* *21*(11), 1463–1470. <http://doi.org/10.1080/09540120902883093>

Karam-Hage, L. N., & Kirk, M. (2001). Modifying residents’ professional attitudes about substance abuse treatment and training. *American Journal on Addictions*, *10*(1), 40-47. <https://doi.org/10.1080/105504901750160466>

Keville, S., Siddaway, A. P., Rhodes, L., Horley, N., Brown, R., Dove, L., & White, L. (2013). Learning on the front line: Can personal development during problem-based learning facilitate professional development in trainee clinical psychologists? *Reflective Practice*, *14*(6), 717–728. <http://doi.org/10.1080/14623943.2013.815610>

Khan, H. (2002). Effectiveness of a strategic management development program. *Applied H.R.M. Research, 7*(1–2), 49–52. Retrieved from <https://psycnet.apa.org/record/2003-06799-006>

Khanna, S. K., Cheyney, M., & Engle, M. (2009). Cultural competency in health care: Evaluating the outcomes of a cultural competency training among health care professionals. *Journal of the National Medical Association*, *101*(9), 886–892. <http://doi.org/10.1016/s0027-9684(15)31035-x>

Kinn, S., Khuder, S. A., Bisesi, M. S., & Woolley, S. (2000). Evaluation of safety orientation and training programs for reducing injuries in the plumbing and pipefitting industry. *Journal of Occupational and Environmental Medicine, 42*(12), 1142–1147. <https://doi.org/10.1097/00043764-200012000-00004>

Kiriazova, T. K., Neduzhko, O. O., Dufour, M. K., Culyba, R. J., & Myers, J. J. (2014). Evaluation of the effectiveness of HIV voluntary counseling and testing trainings for clinicians in the Odessa region of Ukraine. *Aids and Behavior*, *18*(1), S89–S95. article. <http://doi.org/10.1007/s10461-013-0545-6>

Klieger, A., Ben-Hur, Y., & Bar-Yossef, N. (2010). Integrating laptop computers into classroom: Attitudes, needs, and professional development of science teachers-A case study. *Journal of Science Education and Technology*, *19*(2), 187–198. <https://doi.org/10.1007/s10956-009-9191-1>

Kobak, K. A., Craske, M. G., Rose, R.D., & Wolitsky-Taylor, K. (2013). Web-based therapist training on cognitive behavior therapy for anxiety disorders: A pilot study. *Psychotherapy, 50*(2), 235–247. <https://doi.org/10.1037/a0030568>

Kobak, K. A., Lipsitz, J. D., Markowitz, J. C., & Bleiberg, K. L. (2017). Web-based therapist training in interpersonal psychotherapy for depression: Pilot study. *Journal of Medical Internet Research, 19(*7): e257. <https://doi.org/10.2196/jmir.7966>

Kobak, K., Wolitzky-Taylor, K., Craske, M. G., & Rose R. D (2016). Therapist training on cognitive behavior therapy for anxiety disorders using internet-based technologies. *Cognitive Therapy and Research, 41*(2), 252–265. <http://doi.org/10.1007/s10608-016-9819-4>

Kossman, S., Casper, G. R., Severtson, D. J., Grenier, A.-S., Or, C., Carayon, P., & Brennan, P. F. (2006). Designing study nurses’ training to enhance research integrity: A macroergonomic approach. In AMIA - Annual Symposium proceedings (pp. 439–43). Retrieved from <https://pubmed.ncbi.nlm.nih.gov/17238379/>

Kuhne-Eversmann, L., Eversmann, T., & Fischer, M. R. (2008). Team- and case-based learning to activate participants and enhance knowledge: An evaluation of seminars in Germany. *Journal of Continuing Education in the Health Professions*, *28*(3), 165–171. <http://doi.org/10.1002/chp.175>

Kupfer, D. J., Schatzberg, A. F., Dunn, L. O., Schneider, A. K., Moore, T. L., & DeRosier, M. (2016). Career development institute with enhanced mentoring: A revisit. *Academic Psychiatry*, *40*(3), 424–428. <https://doi.org/10.1007/s40596-015-0362-5>

La Guardia, A. C., Cramer, R. J., Brubaker, M., & Long, M. M. (2019). Community mental health provider responses to a competency-based training in suicide risk assessment and prevention. *Community Mental Health Journal, 55*(2), 257-266. <https://doi.org/10.1007/s10597-018-0314-0>

Lagerkvist, B., Mehic-Basara, N., Ceric, I., & Jacobsson, L. (2013). The Swedish support to Bosnia Herzegovina: Rebuilding mental health services after the war. *Intervention: International Journal of Mental Health, Psychosocial Work & Counselling in Areas of Armed Conflict*, *11*(3), 249–260. <https://doi.org/http://dx.doi.org/10.1097/WTF.0000000000000008>

Lalande, L., King, R., Bambling, M., & Schweitzer, R. D. (2016). Guided respiration mindfulness therapy: Development and evaluation of a brief therapist training program. *Journal of Contemporary Psychotherapy*, *46*(2), 107-116. <https://doi.org/10.1007/s10879-015-9320-5>

Lalonde, L., Normandeau, M., Lamarre, D., Lord, A., Berbiche, D., Corneille, L., Prud’homme, L., & Laliberté, M.-C. (2008). Evaluation of a training and communication-network nephrology program for community pharmacists. *Pharmacy World & Science*, *30*(6), 924–933. <https://doi.org/10.1007/s11096-008-9253-0>

Lancashire, S., Haddock, G., Tarrier, N., Baguley, I., Butterworth, C., Brooker, C., (1997). Effects of training in psychosocial interventions for community psychiatric nurses in England. *Psychiatric Services*, *48*(1), 39-41. <https://doi.org/10.1176/ps.48.1.39>

Laube, R. E., & Higson, F. M (2000). Staff training in cognitive-behavioral family intervention in mental illness using the multiple-family group approach: A pilot study. *Community Mental Health Journal, 36*, 477-490. <https://doi.org/10.1023/A:1001959613430>

Le Scanff, C., & Taugis, J. (2002). Stress management for police special forces. *Journal of Applied Sport Psychology, 14*(4), 330-343. <https://doi.org/10.1080/10413200290103590>

Leandros, E., Gomatos, I. P., Konstadoulakis, M. M., Menenakos, E., Alexakis, N., Alevizos, L., [Albanopoulos](https://pubmed.ncbi.nlm.nih.gov/?term=Albanopoulos+K&cauthor_id=20174940), K., [Karagiannakos](https://pubmed.ncbi.nlm.nih.gov/?term=Karagiannakos+P&cauthor_id=20174940), P., & Fingerhut, A. (2010). Prospective appraisal of a 2-day training course on laparoscopic sleeve gastrectomy: The ELTC experience. *Surgical Endoscopy and Other Interventional Techniques*, *24*(9), 2140–2144. <http://doi.org/10.1007/s00464-010-0912-6>

Leif, T. R. (2008). *A case study of the physics enhancement project for two year colleges, its effects and outcomes on the teaching of undergraduate physics at two year colleges* (Publication No. AAI3339319). [Doctoral dissertation, Kansas State University]*.* Astrophysics Data System.

Lelutiu-Weinberger, C., & Pachankis, J. E. (2017). Acceptability and preliminary efficacy of a lesbian, gay, bisexual, and transgender-affirmative mental health practice training in a highly stigmatizing national context. *LGBT Health*, *4*(5), 360-370. <https://doi.org/10.1089/lgbt.2016.0194>

168. Lemon, S. C., Zapka, J., Li, W., Estabrook, B., Rosal, M., Magner, R., Andersen, V., Borg, A., & Hale, J. (2010). Step ahead. A worksite obesity prevention trial among hospital employees. *American Journal of Preventive Medicine, 38*(1), 27–38. <https://doi.org/10.1016/j.amepre.2009.08.028>

Leung, G. M., Chan, S. S. C., Johnston, J. M., Chan, S. K. K., Woo, P. P. S., Chi, I., & Lam, T. H. (2007). Effectiveness of an elderly smoking cessation counseling training program for social workers: a longitudinal study. *Chest, 131*(4), 1157–1165. <https://doi.org/10.1378/chest.06-1975>

Li, J., Li, J., Huang, Y., & Thornicroft, G. (2014). Mental health training program for community mental health staff in Guangzhou, China: Effects on knowledge of mental illness and stigma. *International Journal of Mental Health Systems, 8*(1), 49. <https://doi.org/10.1186/1752-4458-8-49>

Li, J., Li, J., Thornicroft, G., Yang, H., Chen, W., & Huang, Y. (2015). Training community mental health staff in Guangzhou, China: Evaluation of the effect of a new training model. *BMC Psychiatry, 15*:263. https://doi.org/10.1186/s12888-015-0660-1

Lindheim, M. Ø., & Helgeland, H. (2017). Hypnosis training and education: Experiences with a Norwegian one-year education course in clinical hypnosis for children and adolescents. *American Journal of Clinical Hypnosis*, *59*(3), 282–291. <http://doi.org/10.1080/00029157.2016.1230728>

Lintzeris, N., Ritter, A., & Dunlop, A. (2002). Training primary health care professionals to provide buprenophine and LAAM treatment. *Substance Abuse, 23*(4), 245-54. <http://doi.org/10.1080/08897070209511497>

Lippin, T. M., Eckman, A., Calkin, K. R., & McQuiston, T. H. (2000). Empowerment-based health and safety training: Evidence of workplace change from four industrial sectors. *American Journal of Industrial Medicine,* 38, 697-706*.* [https://doi.org/10.1002/1097-0274(200012)38:6<697::AID-AJIM9>3.0.CO;2-T](https://doi.org/10.1002/1097-0274(200012)38:6%3c697::AID-AJIM9%3e3.0.CO;2-T)

Loughland, C., Kelly, B., Ditton-Phare, P., Sandhu, H., Vamos, M., Outram, S., & Levin, T. (2015). Improving clinician competency in communication about schizophrenia: A pilot educational program for psychiatry trainees. *Academic Psychiatry, 39*(2), 160-164. <https://doi.org/10.1007/s40596-014-0195-7>

Lovell, K., Bee, P., Brooks, H., Cahoon, P., Callaghan, P., Carter, L.-A., Cree, L., Davies, L., Drake, R., Fraser, C., Gibbons, C., Grundy, A., Hinsliff-Smith, K., Meade, O., Roberts, C., Rogers, A., Rushton, K., Sanders, C., Shields, G., Walker, L., & Bower, P. (2018). Embedding shared decision-making in the care of patients with severe and enduring mental health problems: The EQUIP pragmatic cluster randomised trial. *PLOS ONE, 13*(8), e0201533. <https://doi.org/10.1371/journal.pone.0201533>

MacKenzie, J., & MacCallam, J. (2009). Preparing staff to provide bereavement support. *Paediatric Nursing*, *21*(3), 22–24. <https://doi.org/10.7748/paed2009.04.21.3.22.c7031>

Malik, N. M., Silverman, J., Wang, K., & Janczewski, C. (2008a). Domestic violence and dependency courts: The “Greenbook” demonstration experience. *Journal of Interpersonal Violence*, *23*(7), 956–980. <https://doi.org/10.1177/0886260508315122>

Malik, N. M., Ward, K., & Janczewski, C. (2008b). Coordinated community response to family violence: The role of domestic violence service organizations. *Journal of Interpersonal Violence, 23*(7), 933–955. <https://doi.org/10.1177/0886260508315121>

Malt, U. F., Huyse, F. J., Herzog, T., Lobo, A., Rijssenbeek, A. J. M. M., & The ECLW. (1996). The ECLW collaborative study: III. Training and reliability of ICD-10 psychiatric diagnoses in the general hospital setting—An investigation of 220 consultants from 14 European countries. *Journal of Psychosomatic Research*, *41*(5), 451-463. <https://doi.org/10.1016/S0022-3999(96)00213-9>

Manassis, K., Ickowicz, A., Picard, E., Antle, B., McNeill, T., Chahauver, A., Mendlowitz, S., Monga, S., & Adler-Nevo, G. (2009). An innovative child CBT training model for community mental health practitioners in Ontario. *Academic Psychiatry, 33*(5), 394-399. <https://doi.org/10.1176/appi.ap.33.5.394>

Manber, R., Carney, C., Edinger, J., Epstein, D., Friedman, L., Haynes, P. L., [Bradley E Karlin](https://pubmed.ncbi.nlm.nih.gov/?term=Karlin+BE&cauthor_id=22505869), B. E., [Pigeon](https://pubmed.ncbi.nlm.nih.gov/?term=Pigeon+W&cauthor_id=22505869), W.,  [Siebern](https://pubmed.ncbi.nlm.nih.gov/?term=Siebern+AT&cauthor_id=22505869), A. T., & Trockel, M. (2012). Dissemination of CBTI to the non-sleep specialist: Protocol development and training issues. *Journal of Clinical Sleep Medicine*, *8*(2), 209–218. <http://doi.org/10.5664/jcsm.1786>

Markström, U. (2014). Staying the course? Challenges in implementing evidence-based programs in community mental health services. *International Journal of Environmental Research and Public Health, 11*(10), 10752-10769. <https://doi.org/10.3390/ijerph111010752>

Marquardt, N., Robelski, S., & Hoeger, R. (2010). Crew resource management training within the automotive industry: Does it work? *Human Factors*, *52*(2), 308–315. <http://doi.org/10.1177/0018720810366258>

Marsh, J. A., McCombs, J. S., Lockwood, J. R., Martorell, F., Gershwin, D., Naftel, S., Le, V. N., Shea, M., Barney, H., & Crego, A. (2008). *Florida’s middle school reading coaches: What do they do? Are they effective?* Research Brief. RAND Corporation. <https://doi.org/10.7249/RB9374>

Masny, A., Ropka, M. E., Peterson, C., Fetzer, D., & Daly, M. B. (2008). Mentoring nurses in familial cancer risk assessment and counseling: Lessons learned from a formative evaluation. *Journal of Genetic Counseling*, *17*(2), 196–207. <https://doi.org/http://dx.doi.org/10.1007/s10897-007-9140-1>

Matthieu, M. M., Chen, Y., Schohn, M., Lantinga, L. J., & Knox, K. L. (2009). Educational preferences and outcomes from suicide prevention training in the veterans health administration: One-year follow-up with healthcare employees in upstate New York. *Military Medicine, 174*(11), 1123–1131. <https://doi.org/10.7205/MILMED-D-00-1109>

Matthieu, M. M., Cross, W., Batres, A. R., Flora, C. M., & Knox, K. L. (2008). Evaluation of gatekeeper training for suicide prevention in veterans. *Archives of Suicide Research*, *12*(2), 148–154. <http://doi.org/10.1080/13811110701857491>

Maurer, M. S., Costley, A. W., Miller, P. A., McCabe, S., Dubin, S., Cheng, H., [Varela-Burstein](https://pubmed.ncbi.nlm.nih.gov/?term=Varela-Burstein+E&cauthor_id=16551323), E., [Lam](https://pubmed.ncbi.nlm.nih.gov/?term=Lam+B&cauthor_id=16551323), B., [Irvine](https://pubmed.ncbi.nlm.nih.gov/?term=Irvine+C&cauthor_id=16551323), C.  [Page](https://pubmed.ncbi.nlm.nih.gov/?term=Page+KP&cauthor_id=16551323), K. P., [Ridge](https://pubmed.ncbi.nlm.nih.gov/?term=Ridge+G&cauthor_id=16551323), G., & Gurland, B. (2006). The Columbia cooperative aging program: An interdisciplinary and interdepartmental approach to geriatric education for medical interns. *Journal of the American Geriatrics Society, 54*(3), 520–526. <https://doi.org/10.1111/j.1532-5415.2005.00616.x>

McAiney, C. A., Stolee, P., Hillier, L. M., Harris, D., Hamilton, P., Kessler, L., Madsen, V., & Le Clair, J. K. (2007). Evaluation of the sustained implementation of a mental health learning initiative in long-term care. *International Psychogeriatrics, 19*(5), 842–858. <https://doi.org/10.1017/S1041610206004443>

McAllan, W., & MacRae, R. (2010). Learning to lead: Evaluation of a leadership development programme in a local authority social work service. *Social Work and Social Sciences Review*, *14*(2), 55–72. <https://doi.org/http://dx.doi.org/10.1921/095352210X557619>

McColgan, M. D., Cruz, M., McKee, J., Dempsey, S. H., Davis, M. B., Barry, P., Yoder, L, & Giardino, A. P. (2010). Results of a multifaceted intimate partner violence training program for pediatric residents. *Child Abuse & Neglect: The International Journal*, *34*(4), 275–283. <https://doi.org/10.1016/j.chiabu.2009.07.008>

McDonald, R. M. (1991). Assessment of organizational context: A missing component in evaluations of training programs. *Evaluation and Program Planning, 14*(4), 273-279. <https://doi.org/10.1016/0149-7189(91)90009-6>

McDougle, L., Ukockis, G., & Adamshick, L. (2010). Evaluation of a new cultural competency training program: CARE Columbus. *Journal of The National Medical Association, 102,* 756-760. [https://doi.org/10.1016*/*S0027*-*9684*(*15*)*30671-4](https://doi.org/10.1016/S0027-9684(15)30671-4)

McIlwraith, R. D., Dyck, K. G., Holms, V. L., Carlson, T. E., & Prober, N. G. (2005). Manitoba’s rural and northern community-based training program for psychology interns and residents. *Professional Psychology: Research and Practice, 36*(2), 164-172. <https://doi.org/10.1037/0735-7028.36.2.164>

McKnight, T. J., & Kearney, C. A. (2001). Staff training regarding choice availability for persons with mental retardation: A preliminary analysis. *Journal of Developmental and Physical Disabilities, 11.* https://doi.org/10.1023/A:1026532631438

McMillen, J. C., Hawley, K. M., & Proctor, E. K. (2016). Mental health clinicians’ participation in web-based training for an evidence supported intervention: Signs of encouragement and trouble ahead. *Administration and Policy in Mental Health and Mental Health Services Research, 43(*4), 592-603. <https://doi.org/10.1007/s10488-015-0645-x>

McNiel, D. E., Fordwood, S. R., Weaver, C. M., Chamberlain, J. R., Hall, S. E., & Binder, R. L. (2008). Effects of training on suicide risk assessment. *Psychiatric Services, 59*(12), 1462-1465. <https://doi.org/10.1176/ps.2008.59.12.1462>

Mellor, D., Kiehne, M., McCabe, M. P., Davison, T. E., Karantzas, G., & George, K. (2010). An evaluation of the beyondblue Depression Training Program for aged care workers. *International Psychogeriatrics, 22*(6), 927-937. <https://doi.org/10.1017/S1041610210000153>

Metalios, E. E., Asgary, R. G., Cooperman, N., Smith, C. L., Du, E., Modali, L., & Sacajiu, G. (2008). Teaching residents to work with torture survivors: Experiences from the Bronx human rights clinic. *Journal of General Internal Medicine*, *23*(7), 1038–1042. <https://doi.org/http://dx.doi.org/10.1007/s11606-008-0592-2>

Miller, P. (2003). Workplace learning by action learning: a practical example. *Journal of Workplace Learning, 15*(1), 14–23. <https://doi.org/10.1108/13665620310458785>

Mirick, R., McCauley, J., Bridger, J., & Berkowitz, L. (2016). continuing education on suicide assessment and crisis intervention: What can we learn about the needs of mental health professionals in community practice? *Community Mental Health Journal*, *52*(5), 501-510. <https://doi.org/10.1007/s10597-015-9884-2>

Mohammadi, S. M., Mohammadi, S. F., Hedges, J. R., Zohrabi, M., & Ameli, O. (2007). Introduction of a quality improvement program in a children’s hospital in Tehran: design, implementation, evaluation and lessons learned. *International Journal for Quality in Health Care, 19*(4), 237–43. <https://doi.org/10.1093/intqhc/mzm021>

Moll, S. E., Patten, S., Stuart, H., MacDermid, J. C., & Kirsh, B. (2018). Beyond silence: A randomized, parallel-group trial exploring the impact of workplace mental health literacy training with healthcare employees. *The Canadian Journal of Psychiatry, 63*(12), 826-833. <https://doi.org/10.1177/0706743718766051>

Monsen, J. J., Brown, E., Akthar, Z., & Khan, S. Y. (2009). An evaluation of a pre-training assistant educational psychologist programme. *Educational Psychology in Practice*, *25*(4), 369–383. <https://doi.org/10.1080/02667360903315180>

Moreno-Andrés, M. V., Quesada-Pallares, C., & Pineda-Herrero, P. (2010). The “working-group” as an innovator method of teacher training to enhancing transfer of learning. *Revista Española de Pedagogía*, *68*(246), 281–295. Retrieved from: <https://revistadepedagogia.org/lxviii/no-246/el-grupo-de-trabajo-como-metodo-innovador-de-formacion-del-profesorado-para-potenciar-la-transferencia-del-aprendizaje/101400010144/>

Morrell, C. J., Ricketts, T., Tudor, K., Williams, C., Curran, J., & Barkham, M. (2011). Training health visitors in cognitive behavioural and person-centred approaches for depression in postnatal women as part of a cluster randomised trial and economic evaluation in primary care: the PoNDER trial. *Primary Health Care Research & Development*, *12*(1), 11–20. <https://doi.org/10.1017/S1463423610000344>

Moyers, T. B., Manuel, J. K., Wilson, P. G., Hendrickson, S. M. L., Talcott, W., & Durand, P. (2008). A randomized trial investigating training in motivational interviewing for behavioral health providers. *Behavioural and Cognitive Psychotherapy*, *36*(2), 149-162. <https://doi.org/10.1017/S1352465807004055>

Murchan, D., Loxley, A., & Johnston, K. (2009). Teacher learning and policy intention: Selected findings from an evaluation of a large-scale programme of professional development in the Republic of Ireland. *European Journal of Teacher Education*, *32*(4), 455–471. <https://doi.org/10.1080/02619760903247292>

Nadeem, E., Weiss, D., Olin, S. S., Hoagwood, K. E., & Horwitz, S. M. (2016). Using a theory-guided learning collaborative model to improve implementation of EBPs in a state children’s mental health system: A pilot study. *Administration and Policy in Mental Health and Mental Health Services Research, 43*(6), 978-990. <https://doi.org/10.1007/s10488-016-0735-4>

Nakamura, B. J., Selbo-Bruns, A., Okamura, K., Chang, J., Slavin, L., & Shimabukuro, S. (2014). Developing a systematic evaluation approach for training programs within a train-the-trainer model for youth cognitive behavior therapy. *Behaviour Research and Therapy*, *53*, 10–19. <http://doi.org/10.1016/j.brat.2013.12.001>

Needham, I., Abderhalden, C., Halfens, R. J. G., Dassen, T., Haug, H. J., & Fischer, J. E. (2005). The effect of a training course in aggression management on mental health nurses’ perceptions of aggression: A cluster randomised controlled trial. *International Journal of Nursing Studies*, *42*(6), 649-655. <https://doi.org/10.1016/j.ijnurstu.2004.10.003>

Neitzel, R., Meischke, H., Daniell, W. E., Trabeau, M., Somers, S., & Seixas, N. S. (2008). Development and pilot test of hearing conservation training for construction workers. *American Journal of Industrial Medicine*, *51*(2), 120–129. <http://doi.org/10.1002/ajim.20531>

Nestel, D., Kneebone, R., Barnet, A., Tierney, T., & Darzi, A. (2010). Evaluation of a clinical communication programme for perioperative and surgical care practitioners. *Quality and Safety in Health Care*, *19*(5). <http://doi.org/10.1136/qshc.2007.026229>

Newes-Adeyi, G., Helitzer, D. L., Roter, D., & Caulfield, L. E. (2004). Improving client-provider communication: Evaluation of a training program for women, infants and children (WIC) professionals in New York state. *Patient Education and Counseling, 55*(2), 210-217. <https://doi.org/10.1016/j.pec.2003.05.001>

Nieto-Montenegro, S., Brown, J. L., & LaBorde, L. F. (2008). Development and assessment of pilot food safety educational materials and training strategies for Hispanic workers in the mushroom industry using the Health Action Model. *Food Control*, *19*(6), 616–633. <http://doi.org/10.1016/j.foodcont.2007.07.005>

Noone, S. J., & Hastings, R. P. (2009). Building psychological resilience in support staff caring for people with intellectual disabilities: pilot evaluation of an acceptance-based intervention. *Journal of Intellectual Disabilities: JOID*, *13*(1), 43–53. <https://doi.org/10.1177/1744629509103519>

O’Donnell, J. M. (2009). Development of an optimal patient transfer task set and simulation-based intervention to reduce musculoskeletal injury in healthcare workers. (Publication No. 9858) [Doctoral Dissertation, University of Pittsburgh]. D-Scholarship@Pitt.

Oettgen, B., Harahsheh, A., Suresh, S., & Kamat, D. (2008). Evaluation of a global health training program for pediatric residents. *Clinical Pediatrics*, *47*(8), 784–790. <http://doi.org/10.1177/0009922808317238>

Oregon, E. (1998). Workplace training project. *ERIC,* 424-443 Retrieved from <http://files.eric.ed.gov/fulltext/ED424443.pdf>

Orton, S., Umble, K. E., Rosen, B., McIver, J., & Menkens, A. J. (2006). Management academy for public health: Program design and critical success factors. *Journal of Public Health Management and Practice, 12*(5), 409–418. <http://doi.org/10.1097/00124784-200609000-00002>.

Palfrey, N., Reay, R. E., Aplin, V., Cubis, J. C., McAndrew, V., Riordan, D. M., & Raphael, B. (2019). Achieving service change through the implementation of a trauma-informed care training program within a mental health service. *Community Mental Health Journal, 55*(3), 467-475. <https://doi.org/10.1007/s10597-018-0272-6>

Panter, K. (1994). Greenhouse Pesticide Application Safety Training. *HortTechnology, 4*(3), 299–301. <https://doi.org/10.21273/HORTTECH.4.3.299>

Papadatou, D. (1997). Training health professionals in caring for dying children and grieving families. *Death Studies 21*(6) 575–600. <https://doi.org/10.1080/074811897201787>

Pepping, C. A., Lyons, A., & Morris, E. M. J. (2018). Affirmative LGBT psychotherapy: Outcomes of a therapist training protocol. *Psychotherapy*, *55*(1), 52-62. <https://doi.org/10.1037/pst0000149>

Petridou, E., & Glaveli, N. (2008). Rural women entrepreneurship within co-operatives: Training support. *Gender in Management*, *23*(4), 262–277. <http://doi.org/10.1108/17542410810878077>

Philotheou, G. M. (2004). Distance assisted training in sub-Saharan Africa: A program evaluation. *Journal of Nuclear Medicine Technology, 32*(3), 166–170. Retrieved from: <https://tech.snmjournals.org/content/32/3/166>

Pisani, A. R., Cross, W. F., Watts, A., & Conner, K. (2012). Evaluation of the Commitment to Living (CTL) curriculum: A 3-hour training for mental health professionals to address suicide risk. *Crisis*, *33*(1), 30-38. <https://doi.org/10.1027/0227-5910/a000099>

Possis, E., Skroch, B., Mallen, M., Henry, J., Hintz, S., Billig, J., & Olson, D. (2016). Brief immersion training in Primary Care–Mental Health Integration: Program description and initial findings. *Training and Education in Professional Psychology*, *10*(1), 24-28. <https://doi.org/10.1037/tep0000103>

Probst, T. M., Gold, D., & Caborn, J. (2008). A preliminary evaluation of SOLVE: Addressing psychosocial problems at work. *Journal of Occupational Health Psychology*, *13*(1), 32–42. <http://doi.org/10.1037/1076-8998.13.1.32>

Prochaska, J. J., Fromont, S. C., Leek, D., Suchanek Hudmon, K., Louie, A. K., Jacobs, M. H., & Hall, S. M. (2008). Evaluation of an evidence-based tobacco treatment curriculum for psychiatry residency training programs. *Academic Psychiatry*, *32*(6), 484-492. <https://doi.org/10.1176/appi.ap.32.6.484>

Quinet, K., Nunn, S., & Kincaid, N. (2003). Training police: A case study of differential impacts of problem-oriented policing training. *Police Practice and Research, 4*(3), 263-283. <http://doi.org/10.1080/1561426032000113889>

Rask, M. T., Jensen, M. L., Andersen, J., & Zachariae, R. (2009). Effects of an intervention aimed at improving nurse-patient communication in an oncology outpatient clinic. *Cancer Nursing*, *32*(1), 1–11. <http://doi.org/10.1097/01.NCC.0000343365.13871.12>

Reed, A. B., Crafton, C., Giglia, J. S., & Hutto, J. D. (2009). Back to basics: Use of fresh cadavers in vascular surgery training. *Surgery, 146*(4), 757–763. <http://doi.org/10.1016/j.surg.2009.06.048>

Reed, E., Crane, D., Svendsen, D., Herman, L., Evans, B., Niedermier, J., Resch, W., Ronis, R., Varley, J., & Welton, R. (2016). behavioral health and primary care integration in Ohio’s psychiatry residency training. *Academic Psychiatry*, *40*(6), 880-886. <https://doi.org/10.1007/s40596-016-0623-y>

Reisman, A. B., Hansen, H., & Rastegar, A. (2006). The craft of writing: A physician-writer’s workshop for resident physicians. *Journal of General Internal Medicine, 21*(10), 1109–1111. <https://doi.org/10.1111/j.1525-1497.2006.00550.x>

Reyes Ortega, M. A., Kuczynski, A. M., Kanter, J. W., de Montis, I. A., & Santos, M. M. (2019). A preliminary test of a social connectedness burnout intervention for Mexican mental health professionals. *The Psychological Record*, *69*(2), 267-276. <https://doi.org/10.1007/s40732-019-00338-5>

Rezvyy, G., Parniakov, A., Fedulova, E., & Olstad, R. (2008). Correcting biases in psychiatric diagnostic practice in Northwest Russia: Comparing the impact of a general educational program and a specific diagnostic training program. *BMC Medical Education, 8*(1), 15. <https://doi.org/10.1186/1472-6920-8-15>

Rice, M. E., Helzel, M. F., Varney, G. W., & Quinsey, V. L. (1985). Crisis prevention and intervention training for psychiatric hospital staff. *American Journal of Community Psychology, 13*(3), 289-304. https://doi.org/[10.1007/BF00914934](https://doi.org/10.1007/bf00914934)

Ridde, V., Fournier, P., Banza, B., Tourigny, C., & Ouédraogo, D. (2009). Programme evaluation training for health professionals in francophone Africa: Process, competence acquisition and use. *Human Resources for Health*, *7*(1) <http://doi.org/10.1186/1478-4491-7-3>

Robinson, T., Hills, D., & Kelly, B. (2011). The evaluation of an online orientation to rural mental health practice in Australia. *Journal of Psychiatric and Mental Health Nursing*, *18*(7), 629–636. <http://doi.org/10.1111/j.1365-2850.2011.01712.x>

Ross, S., Barton, J., & Read, J. (2009). Staff in-service training on post-stroke psychological and communication issues. *International Journal of Therapy and Rehabilitation*, *16*(6), 342–348. <http://doi.org/10.12968/ijtr.2009.16.6.42437>

Rounsaville, B. J., Chevron, E. S., Weissman, M. M., Prusoff, B. A., & Frank, E. (1986). Training therapists to perform interpersonal psychotherapy in clinical trials. *Comprehensive Psychiatry*, *27*(4), 364-371. <https://doi.org/10.1016/0010-440X(86)90012-X>

Ruzek, J. I., Eftekhari, A., Rosen, C. S., Crowley, J. J., Kuhn, E., Foa, E. B., Hembree, E. A., & Karlin, B. E. (2016). Effects of a comprehensive training program on clinician beliefs about and intention to use prolonged exposure therapy for PTSD. Psychological Trauma: Theory, Research, Practice, and Policy, 8(3), 348–355. <https://doi.org/10.1037/tra0000004>

Sale, E. (2018). Counseling on Access to Lethal Means (CALM): An evaluation of a suicide prevention means restriction training program for mental health providers. *Community Mental Health Journal, 54*(1). https://doi.org/10.1007/s10597-017-0190-z

Salyers, M. P., Hudson, C., Morse, G., Rollins, A. L., Monroe-DeVita, M., Wilson, C., & Freeland, L. (2011). BREATHE: A pilot study of a one-day retreat to reduce burnout among mental health professionals. *Psychiatric Services*, *62*(2), 214-217. <https://doi.org/10.1176/ps.62.2.pss6202_0214>

Saunders, D., Holter, M., Pahl, L., & Tolman, R. (2006). Welfare workers’ responses to domestic violence cases: The effects of training and worker characteristics. *Families in Society: The Journal of Contemporary Social Services, 87*(3), 329–338. https://doi.org/10.1606/1044-3894.3537

Scardovi, A., Rucci, P., Gask, L., Berardi, D., Leggieri, G., Berti Ceroni, G., & Ferrari, G. (2003). Improving psychiatric interview skills of established GPs: Evaluation of a group training course in Italy. *Family Practice, 20*(4), 363–369, <https://doi.org/10.1093/fampra/cmg404>

Schoener, E. P., Madeja, C. L., Henderson, M. J., Ondersma, S. J., & Janisse, J. J. (2006). Effects of motivational interviewing training on mental health therapist behavior. *Drug and Alcohol Dependence*, *82*(3), 269-275. <https://doi.org/10.1016/j.drugalcdep.2005.10.003>

Sears, K. E., Cohen, J. E., & Drope, J. (2008). Comprehensive Evaluation of an Online Tobacco Control Continuing Education Course in Canada. *Journal of Continuing Education in the Health Professions*, *28*(4), 235–240. <http://doi.org/10.1002/chp.190>

Seman, K., Yaacob, H., Hamid, A. M. H., Ismail, A. R., & Yusoff, A. (2008). Evaluation of a training programme for non-health professionals as oral health educators. *Malaysian Journal of Medical Sciences*, *15*(2), 33–36. Retrieved from https://pubmed.ncbi.nlm.nih.gov/22589623/

Shafer, M. S., Rhode, R., & Chong, J. (2004). Using distance education to promote the transfer of motivational interviewing skills among behavioral health professionals. *Journal of Substance Abuse Treatment, 26*, 141–148 https://doi.org/10.1016/S0740-5472(03)00167-3

Shamrock, J. (2009). Evaluation of a pilot community based rehabilitation training programme in east timor. *Asia Pacific Disability Rehabilitation Journal*, *20*(2), 17–31. Retrieved from https://dev.asksource.info/resources/evaluation-a-pilot-community-based-rehabilitation-training-programme-east-timor

Sholomskas, D. E., Syracuse-Siewert, G., Rounsaville, B. J., Ball, S. A., Nuro, K. F., & Carroll, K. M. (2005). We don't train in vain: A dissemination trial of three strategies of training clinicians in cognitive-behavioral therapy. *Journal of consulting and clinical psychology*, *73*(1), 106–115. <https://doi.org/10.1037/0022-006X.73.1.106>

Silva, L. C., Teixeira, M. C. T. V., Ribeiro, E. L., & Paula, C. S. (2017). Impact of a provider training program on the treatment of children with autism spectrum disorder at psychosocial care units in Brazil. *Revista Brasileira de Psiquiatria, 40*(3), 296-305. <https://doi.org/10.1590/1516-4446-2016-2090>

Simons. (2000). An Evaluation of an oral health training programme for carers of the elderly in residential homes. *British Dental Journal, 188*(4), 206–210. <https://doi.org/10.1038/sj.bdj.4800432>

Sinclair, R. C., Smith, R., Colligan, M., Prince, M., Nguyen, T., & Stayner, L. (2003). Evaluation of a safety training program in three food service companies. *Journal of Safety Research, 34*(5), 547–558. <https://doi.org/10.1016/j.jsr.2003.03.003>

Sørensen, J. L., Løkkegaard, E., Johansen, M., Ringsted, C., Kreiner, S., & McAleer, S. (2009). The implementation and evaluation of a mandatory multi-professional obstetric skills training program. *Acta Obstetricia et Gynecologica Scandinavica*, *88*(10), 1107–1117. <http://doi.org/10.1080/00016340903176834>

Spagnolo, J., Champagne, F., Leduc, N., Melki, W., Piat, M., Laporta, M., Bram, N., Guesmi, I., & Charfi, F. (2018). “We find what we look for, and we look for what we know”: Factors interacting with a mental health training program to influence its expected outcomes in Tunisia. *BMC Public Health, 18*(1), 1398. <https://doi.org/10.1186/s12889-018-6261-4>

Spielberger, J., Baker, S., Winje, C., & Mayers, L. (2009). Getting ready for school: The early childhood cluster initiative of Palm Beach County, Florida. Program implementation and early outcomes: Year 3 report. University of Chicago. Retrieved from <https://search.proquest.com/docview/742875745?accountid=14744>

Sporrong, S., Arnetz, B., Hansson, M., Westerholm, P., & Höglund, A. (2007). Developing ethical competence in health care organizations. *Nursing Ethics, 14*(6), 825–837. <https://doi.org/110.1177/0969733007082142>.

Sprague, S. (2019). A qualitative evaluation of the implementation of an intimate partner violence education program in fracture clinics. *Journal of Family Violence, 34*, 621–630. <https://doi.org/10.1007/s10896-019-00052-4>

Stanhope, V., Ross, A., Choy-Brown, M., & Jessell, L. (2019). A mixed methods study of organizational readiness for change and leadership during a training initiative within community mental health clinics. *Administration and Policy in Mental Health, 46*(5), 678–687. <https://doi.org/10.1007/s10488-019-00946-x>

Stein, J., Lewin, S., Fairall, L., Mayers, P., English, R., Bheekie, A., Bateman, E., & Zwarenstein, M. (2008). Building capacity for antiretroviral delivery in South Africa: A qualitative evaluation of the PALSA PLUS nurse training programme. *BMC Health Services Research, 8*(1):240. <https://doi.org/10.1186/1472-6963-8-240>

Steinert, Y., Nasmith, L., Daigle, N., & Franco, E. (2001). Improving teachers' skills in working with 'problem' residents: a workshop description and evaluation. *Medical Teacher, 23*(3), 284-288. <https://doi.org/10.1080/01421590120048139>.

Stergiou, N., Georgoulakis, G., Margari, N., Aninos, D., Stamataki, M., Stergiou, E., Pouliakis, A., & Karakitsos, P. (2009). Using a web-based system for the continuous distance education in cytopathology. *International Journal of Medical Informatics*, *78*(12), 827–838. <http://doi.org/10.1016/j.ijmedinf.2009.08.007>

Stock, M. L., Gerrard, M., Gibbons, F. X., Dykstra, J. L., Mahler, H. I. M., Walsh, L. A., & Kulik, J. A. (2009). Sun protection intervention for highway workers: Long-term efficacy of UV photography and skin cancer information on men’s protective cognitions and behavior. *Annals of Behavioral Medicine*, *38*(3), 225-236. <https://doi.org/10.1007/s12160-009-9151-2>

Straus, S. E., Graham, I. D., Taylor, M., & Lockyer, J. (2008). Development of a mentorship strategy: A knowledge translation case study. *Journal of Continuing Education in the Health Professions*, *28*(3), 117–122. Retrieved from <https://search.proquest.com/docview/61970912?accountid=14744>

Stuart, S., Schultz, J., & Ashen, C. (2018). A new community-based model for training in evidence-based psychotherapy practice. *Community Mental Health Journal, 54*(7), 912-920. <https://doi.org/10.1007/s10597-017-0220-x>

Stuhlmann, J., Daniel, C., Dellinger, A., Denny, R. K., & Powers, T. (1999). A generalizability study of the effects of training on teachers’ abilities to rate children’s writing using a rubric Introduction and Purpose. *Reading Psychology,* *20*(2), 107-127, <http://doi.org/10.1080/027027199278439>

Swierczek, F. W., & Carmichael, L. (1985). The quantity and quality of evaluating training. *Training & Development Journal, 39*(1), 95–99. Retrived from <https://psycnet.apa.org/record/1985-16041-001>

Teleki, S. S., Damberg, C. L., Sorbero, M. E. S., Shaw, R. N., Bradley, L. A., Quigley, D. D., Fremont, A. M., & Farley, D. O. (2009). Training a patient safety work force: The patient safety improvement corps. *Health Services Research*, *44*(2P2), 701–716. <http://doi.org/10.1111/j.1475-6773.2008.00927.x>

Tharp-Taylor, S., Nelson, C. A., Hamilton, L. S., & Yuan, K. (2009). Pittsburgh public schools’ excellence for all: Year 2 evaluation. Documented Briefing. DB-575-PPS. RAND Corporation. Retrieved from <https://search.proquest.com/docview/61882158?accountid=14744>

Thomas, C. W., Guy, S. M., & Ogilvie, L. P. (1999). An evaluation of a practitioner training program designed to assist families of people with severe psychiatric disorders. *Psychiatric Rehabilitation Journal, 23(*1), 34-41. <https://doi.org/10.1037/h0095197>

Thomas, L. S., Jina, R., Tint, K. S., & Fonn, S. (2007). Making systems work: The hard part of improving maternal health services in South Africa. *Reproductive Health Matters, 15*(30), 38–49. <https://doi.org/10.1016/S0968-8080(07)30314-5>

Thompson, A. R., Donnison, J., Warnock-Parkes, E., Turpin, G., Turner, J., & Kerr, I. B. (2008). Multidisciplinary community mental health team staff’s experience of a “skills level” training course in cognitive analytic therapy. *International Journal of Mental Health Nursing*, *17*(2), 131–137. <http://doi.org/10.1111/j.1447-0349.2008.00521.x>

Thompson, D., Brooks, K., & Lizárraga, E. (2003). Perceived transfer of learning: From the distance education classroom to the workplace. *Assessment & Evaluation in Higher Education, 28*(5), 539-547, <https://doi.org/10.1080/02602930301680>

Thurston, A., Christie, D., Howe, J. C., Tolmie, A., & Topping, J. K. (2008). Effects of continuing professional development on group work practices in Scottish primary schools. *Journal of In-Service Education*, *34*(3), 263–282. <https://doi.org/10.1080/13674580802264803>

Timmons-Mitchell, J., Albright, G., McMillan, J., Shockley, K, & Cho, S. (2019). Virtual role-play: Middle school educators addressing student mental health. *Health Behavior and Policy Review, 6*(6), 546-557. <https://doi.org/10.14485/HBPR.6.6.1ç>

Tompkins, T. L., Witt, J., & Abraibesh, N. (2009). Does a gatekeeper suicide prevention program work in a school setting? Evaluating training outcome and moderators of effectiveness. *Suicide and Life-Threatening Behavior*, *39*(6), 671–681. <http://doi.org/10.1521/suli.2009.39.6.671>

Triplett, N. S., Sedlar, G., Berliner, L., Jungbluth, N., Boyd, M., & Dorsey, S. (2019). Evaluating a train-the-trainer approach for increasing EBP training capacity in community mental health. *The Journal of Behavioral Health Services & Research, 47*(2), 189-200. <https://doi.org/10.1007/s11414-019-09676-2>

Turcotte, D., Lamonde, G., & Beaudoin, A. (2009). Evaluation of an in-service training program for child welfare practitioners. *Research on Social Work Practice*, *19*(1), 31–41. <http://doi.org/10.1177/1049731507313978>

Umble, K. E., Brooks, J., Lowman, A., Malison, M., Huong, N. T., Iademarco, M., & Laserson, K. (2009). Management training in Vietnam's National Tuberculosis Program: an impact evaluation. *The International Journal of Tuberculosis and Lung Disease, 13*(2), 238–246. Retrieved from <https://pubmed.ncbi.nlm.nih.gov/19146754/>

Van Doesum, K., Maia, T., Pereira, C., Loureiro, M., Marau, J., Toscano, L., Lauritzen, C., & Reedtz, C. (2019). The impact of the “Semente” program on the family-focused practice of mental health professionals in Portugal. *Frontiers in Psychiatry*, *10*, 305. <https://doi.org/10.3389/fpsyt.2019.00305>

Van Landschoot, R., Portzky, G., & van Heeringen, K. (2017). Knowledge, self-confidence and attitudes towards suicidal patients at emergency and psychiatric departments: A randomised controlled trial of the effects of an educational poster campaign. *International Journal of Environmental Research and Public Health, 14*(3), 304. <https://doi.org/10.3390/ijerph14030304>

Van Oordt, M.S.; Jobes, D.A.; Fonseca, V.P.; Schmidt, S.M. (2009). Training mental health professionals to assess and manage suicidal behavior: Can provider confidence and practice behaviors be altered? *Suicide Life-Threat. Behavior, 39*(1), 21–32. <https://doi.org/10.1521/suli.2009.39.1.21>

Vingård, E., Blomkvist, V., Rosenblad, A., Lindberg, P., Voss, M., Alfredsson, L., & Josephson, M. (2009). A physical fitness programme during paid working hours - Impact on health and work ability among women working in the social service sector: A three year follow up study. *Work*, *34*(3), 339–344. <http://doi.org/10.3233/WOR-2009-0932>

Wakefield, A., Carlisle, C., Hall, A., & Attree, M. (2009). Patient safety investigations: The need for interprofessional learning. *Learning in Health and Social Care*, *8*(1), 22–32. <https://doi.org/http://dx.doi.org/10.1111/j.1473-6861.2008.00192.x>

Wallace, L. M., Spurgeon, P., Adams, S., Earll, L., & Bayley, J. (2009). Survey evaluation of the National Patient Safety Agency’s Root Cause Analysis training programme in England and Wales: Knowledge, beliefs and reported practices. *Quality and Safety in Health Care*, *18*(4), 288–291. <http://doi.org/10.1136/qshc.2008.027896>

Wang, C., Wei, S., Xiang, H., Wu, J., Xu, Y., Liu, L., & Nie, S. (2008). Development and evaluation of a leadership training program for public health emergency response: results from a Chinese study. *BMC Public Health*, *8*(1), 377. <https://doi.org/10.1186/1471-2458-8-377>

Wang, D., Operario, D., Hong, Q., Zhang, H., & Coates, T. J. (2009). Intervention to train physicians in rural China on HIV/STI knowledge and risk reduction counseling: Preliminary findings. *AIDS Care - Psychological and Socio-Medical Aspects of AIDS/HIV*, *21*(4), 468–472. <http://doi.org/10.1080/09540120802290357>

Weist, M., Lever, N., Stephan, S., Youngstrom, E., Moore, E., Harrison, B., Anthony, L., Rogers, K., Hoagwood, K., Ghunney, A., Lewis, K., & Stiegler, K. (2009). Formative evaluation of a framework for high quality, evidence-based services in school mental health. *School Mental Health*, *1*(4), 196-211. <https://doi.org/10.1007/s12310-009-9018-5>

Whitmore, S. C., Grefsheim, S. F., & Rankin, J. A. (2008) Informationist programme in support of biomedical research: A programme description and preliminary findings of an evaluation. *Health Information and Libraries Journal*, *25*(2), 135-41. <https://doi.org/10.1111/j.1471-1842.2007.00756.x>

Wilkinson, S. M., Leliopoulou, C., Gambles, M., & Roberts, A. (2003). Can intensive three-day programmes improve nurses' communication skills in cancer care? *Psychooncology, 12*(8), 747-59. <https://doi.org/10.1002/pon.698>.

Williams, V. P., Brenner, S. L., Helms, M. J., & Williams, R. B. (2009). Coping skills training to reduce psychosocial risk factors for medical disorders: A field trial evaluating effectiveness in multiple worksites. *Journal of Occupational Health*, *51*(5), 437–442. <http://doi.org/10.1539/joh.O8016>

Wills, J., Reynolds, J., & Swanwick, T. (2009). “Just a lovely luxury?” What can public health attachments add to postgraduate general practice training? *Education for Primary Care*, *20*(4), 278–284. http://doi.org/0.1080/14739879.2009.11493799

Wilson, J. D. (2000). Training Bangladeshi professors to be teacher trainers. *Asia-Pacific Journal of Teacher Education,* *28*(1), 39-51. <https://doi.org/10.1080/135986600109435>

Wiltsey Stirman, S., Pontoski, K., Creed, T., Xhezo, R., Evans, A. C., Beck, A. T., & Crits-Christoph, P. (2017). A non-randomized comparison of strategies for consultation in a community-academic training program to implement an evidence-based psychotherapy. *Administration and Policy in Mental Health and Mental Health Services Research,* *44*, 55–66. DOI 10.1007/s10488-015-0700-7

Wortman, P. M. (1977). Evaluation research: New training programs for new careers. *Professional Psychology, 8*(3), 361–367. [https://doi.org/10.1037/0735-7028.8.3.361](%20https://doi.org/10.1037/0735-7028.8.3.361)

Wright, J. A., Stackhouse, J., & Wood, J. (2008). Promoting language and literacy skills in the early years: Lessons from interdisciplinary teaching and learning. *Child Language Teaching and Therapy*, *24*(2), 155–171. <http://doi.org/10.1177/0265659007090292>

Zipple, A., Spaniol, L., & Rogers, E. S. (1990). Training mental health practitioners to assist families of persons who have a psychiatric disability. Rehabilitation Psychology, 35(2), 121–129. <https://doi.org/10.1037/h0079055>
