# Supplementary material for: The Methodological Quality Scale (MQS) for intervention programs: validity evidence
Source: Front Psychol. 2023 Jul 6;14:1217661. doi: 10.3389/fpsyg.2023.1217661 (PMC10358327; doi:10.3389/fpsyg.2023.1217661)
Supplement: Supplementary file 5 [file Data_Sheet_5.DOCX]

Supplementary Material

The Methodological Quality Scale (MQS) for Intervention Programs: Validity Evidence

**Salvador Chacón-Moscoso*, Susana Sanduvete-Chaves, José Antonio Lozano-Lozano, Francisco Pablo Holgado-Tello**

*** Correspondence:** Salvador Chacón Moscoso: schacon@us.es

# Supplementary Data S5

**Methodological Quality Scale (MQS)**

**External Validity**

1. **Inclusion and exclusion criteria for the units provided**: explicit reasons provided as to why certain people were able to participate in the study and others were not:
2. **No:** no explicit selection criteria for units AND with exceptions in their application; information unavailable.

**0.5. Intermediate:** explicit selection criteria for units OR applied to all potential participants.

**1. Yes (replicable):** explicit selection criteria for units AND applied to all potential participants.

1. **Attrition:** loss of units. In randomized experiments, this refers to loss that occurred after the random assignment, i.e., the number of participants from the initial sample that did not conclude the study (e.g., N pre minus N post).
   1. **Unspecified:** information is not available and cannot be calculated AND reasons for loss of units are not specified.

**0.5. Intermediate:** number of units lost is specified or can be calculated OR reasons for loss of units are specified.

**1. Specified:** no units are lost, or number of units lost is specified or can be calculated AND reasons for loss of units are specified.

1. **Attrition between groups:** this item evaluated the differences in attrition between two groups.
   1. **Unspecified:** information is not available and cannot be calculated AND reasons for attrition between groups are not specified.

**0.5. Intermediate:** number of lost units is specified or can be calculated OR reasons for attrition between groups are specified.

**1. Specified:** no units were lost, or number of lost units is specified or can be calculated AND reason/s for the attrition between groups is/are specified.

**9.** **Not applicable:** no cross-group comparison.

1. **Statistical methods for imputing missing data**: to estimate what the study would have yielded had there been no attrition:
   1. **High risk:** it is not clear if there was attrition, or there was attrition and calculations to estimate effects were carried out without imputing missing data.

**0.5. Medium risk:** values for the missing data points were imputed so they could be included in the analyses. The method used was specified, i.e., sample mean substitution, last value forward method for longitudinal data sets, hot deck imputation, single imputation (e.g., imputation, regression imputation), or multiple imputation (e.g., likelihood ratio test after multiple imputation). The reasons for choosing the specific method were not specified.

- 1. **Low risk:** there was no attrition or values for the missing data points were imputed so they could be included in the analyses; and the specific method used AND the reasons for choosing the specific method were specified.

**External validity score:**

Add the scores obtained in items 1 – 4 and divide by the number of items. If item 3 is not applicable, do not add a score for that item and divide the summation of items 1, 2 and 4 by 3.

*****************************

**Internal validity**

1. **Methodology or design:** something an experimenter could manipulate or control in an experiment to help address a threat to validity:
   1. **Pre-experimental/others** (questionnaires/observational/naturalistic): a study with only one group and a maximum of two measurement occasions for the same dependent variable (e.g., pre-post design); or when there are two groups and only one measure (e.g., control-experimental design).
   2. **Quasi-experimental** (two groups without randomized assignment) non-equivalent control groups with pre-test and post-test; or one group with three or more measures of the same dependent variable (even without pretest): an experiment (exploration of the effects of manipulating a variable) in which units are not randomly assigned to conditions.
   3. **Experimental; randomized:** an experiment (exploration of the effects of manipulating a variable in which units are randomly assigned to conditions.
2. **Follow-up period**: the amount of time between the first post-intervention measurements and any additional measurements. When the study presented more than one follow-up period, the longest was considered.
   1. No follow-up or less than two months.
   2. Between two and six months (both included).
   3. More than six months.
3. **Measurement occasions for each dependent variable**: this item specified when the measurements were taken.
   1. **Post-intervention only:** all measurements were taken after the intervention.
   2. **Pre- and post-intervention:** some measurements were taken before and immediately after the intervention.
4. **Pre-, post-intervention and follow-up period:** some measurements were taken before, immediately after the intervention, and again at a later date.
5. **Control techniques**:
   1. **None:** no control technique is specified or described.
   2. **Masking OR other/s:** masking, also known as double-blinding, refers to a procedure that prevented participants and/or experimenters from knowing the hypotheses; OR any other control technique was used (e.g., matching, stratifying, counterbalancing, constant, participant as own experimental control -longitudinal-).
   3. **Masking AND other:** masking AND at least one other control technique.

**Internal validity score:**

Add the scores obtained in items 5 – 8 and divide by the number of items (4).

*****************************

**Construct validity**

1. **Standardization of the dependent variables:** level of normalization of the tool to measure the variable that varied in response to the independent variable (also called effect or outcome).
   1. **Low standardization (self-reports and post hoc records)**: all measurements were taken using ad hoc tools, developed in a specific situation, and without any study of their psychometric properties.

**0.5. Medium standardization**: at least one measurement was taken using structured tools with ONE study of their psychometric properties (reliability or one form of validity evidence).

- 1. **High standardization**: at least one measurement was taken using structured tools. At least TWO studies of their psychometric properties (reliability, validity, construction of scaling) were carried out.

1. **Construct definition of outcome**: explanation of the concept, model, or schematic idea measured as a dependent variable:
   1. **No definition:** no concept treated as a dependent variable was measured in a conceptual or empirical way.
   2. **Vague definition:** at least one concept treated as a dependent variable was defined in a conceptual and/or empirical way.
   3. **Replicable by reader in own setting:** all concepts treated as dependent variables were defined in a conceptual and empirical way.

**Construct validity score:**

Add the scores obtained in items 9 and 10 and divide by the number of items (2).

*****************************

**INTERPRETATION for each type of validity:**

| **Score** | **Interpretation** |
| --- | --- |
| < 0.5 | Low |
| [0.5 – 0.75] | Medium |
| > 0.75 | High |
